# A computational model of skeletal muscle metabolism linking cellular adaptations induced by altered loading states to metabolic responses during exercise

**DOI:** 10.1186/1475-925X-6-14

**Published:** 2007-04-20

**Authors:** Ranjan K Dash, John A DiBella, Marco E Cabrera

**Affiliations:** 1 Department of Biomedical Engineering, Case Western Reserve University, Cleveland, OH, USA; 2 Department of Physiology and Biophysics, Case Western Reserve University, Cleveland, OH, USA; 3 Department of Pediatrics, Case Western Reserve University, Cleveland, OH, USA; 4 Center for Modeling Integrated Metabolic Systems, Case Western Reserve University, Cleveland, OH, USA; 5 Biotechnology and Bioengineering Center, Department of Physiology, Medical College of Wisconsin, Milwaukee, WI 53226, USA

## Abstract

**Background:**

The alterations in skeletal muscle structure and function after prolonged periods of unloading are initiated by the chronic lack of mechanical stimulus of sufficient intensity, which is the result of a series of biochemical and metabolic interactions spanning from cellular to tissue/organ level. Reduced activation of skeletal muscle alters the gene expression of myosin heavy chain isoforms to meet the functional demands of reduced mechanical load, which results in muscle atrophy and reduced capacity to process fatty acids. In contrast, chronic loading results in the opposite pattern of adaptations.

**Methods:**

To quantify interactions among cellular and skeletal muscle metabolic adaptations, and to predict metabolic responses to exercise after periods of altered loading states, we develop a computational model of skeletal muscle metabolism. The governing model equations – with parameters characterizing chronic loading/unloading states- were solved numerically to simulate metabolic responses to moderate intensity exercise (WR ≤ 40% VO_2 max_).

**Results:**

Model simulations showed that carbohydrate oxidation was 8.5% greater in chronically unloaded muscle compared with the loaded muscle (0.69 vs. 0.63 mmol/min), while fat oxidation was 7% higher in chronically loaded muscle (0.14 vs. 0.13 mmol/min), during exercise. Muscle oxygen uptake (VO_2_) and blood flow (Q) response times were 29% and 44% shorter in chronically loaded muscle (0.4 vs. 0.56 min for VO_2 _and 0.25 vs. 0.45 min for Q).

**Conclusion:**

The present model can be applied to test complex hypotheses during exercise involving the integration and control of metabolic processes at various organizational levels (cellular to tissue) in individuals who have undergone periods of chronic loading or unloading.

## Background

Living organisms have the inherent capacity to adapt to their environment by altering the structural and functional properties of their tissues and/or organ systems. The adaptive process starts by having specific genes and their products undergo altered expression in order to meet the demands imposed by the environmental conditions. In particular, skeletal muscle adapts by altering the contractile protein myosin heavy chain (MHC) in quantity and type of isoform in response to the chronic mechanical perturbation (loading/unloading) imposed on it [1-5]. Thus, skeletal muscle is capable of adapting to interventions involving a chronic mechanical stress which is either increased (e.g., endurance or resistance exercise) or reduced (e.g., chronic inactivity, limb immobilization, or microgravity exposure) by regulating the expression of key enzymes and transport proteins.

Chronically increased physical activity (loading) at levels requiring 70–80% of maximal aerobic capacity, typical of endurance training, has shown to increase muscle capillarization and mitochondrial density and to upregulate the mitochondrial enzyme systems of the Krebs cycle and the electron transport chain [6-11]. Endurance training or chronic loading also lowers glucose uptake and the rate of glycogen breakdown leading to reduced rates of glycolytically-derived pyruvate, pyruvate oxidation, and lactate production [12-14]. In addition, chronic loading increases the expression of the enzymes involved in fatty acid activation, translocation, and oxidation leading to an increased rate of fatty acid oxidation [15-18]. As a consequence, chronically-loaded individuals rely more on fats as the main source of fuel for ATP synthesis, which is offset by a reduction in the relative contribution of intramuscular glycogen [16,19,20].

In contrast, when skeletal muscle is continuously unloaded – as it occurs during microgravity exposure or bed rest, or during chronically reduced physical activity (e.g., limb immobilization or detraining) – the opposite patterns of transformation in muscle metabolic characteristics occur. Chronic unloading induces small reductions in muscle capillarization and mitochondrial density [8,21], as well as a 20–50% reduction in the maximal activities of oxidative enzymes [22,23]. Unloading also leads to an increased contribution from intra-muscular carbohydrates for ATP production, resulting in a greater rate of lactate formation during exercise [22,24]. Associated with this increased reliance in glycogen breakdown is the reduced production of acetyl-CoA from fatty acid oxidation, which is accentuated during periods of increased physical activity [18,22,24]. In addition to these metabolic alterations, chronic unloading results in a significant reduction in fibers cross sectional area in the quadriceps muscle, which leads to a decline in whole muscle volume [8,21], as well as reduced muscle strength and endurance [21,23,25]. Prolonged chronic unloading enhances these alterations leading to further deterioration in muscle function until complete adaptation to physical and energetic demands of the new environment is established [21,23,25].

Skeletal muscle adaptations to chronic loading and unloading also result in different dynamic metabolic responses to acute exercise. Even though the amount of oxygen taken up by muscle cells during the steady-state response (VO_2 ss_) of a sub-maximal constant work rate exercise bout of moderate intensity (WR ≤ 40% VO_2 max_) remains the same regardless of conditioning status of the muscle [26,27], the transient response is different in individuals who have undergone chronic unloading vs. loading. While chronic unloading increases the time needed to reach VO_2 ss _when compared to controls, endurance training or chronic loading decreases this response time [27]. The dynamic response of phosphocreatine (PCr) breakdown at exercise onset shows similar behavior, with the loaded muscle buffer ATP changes and deplete PCr stores faster [6,11].

The changes in the time profiles of step-responses of various metabolic variables elicited by periods of chronic loading or unloading illustrate the significance of (i) collecting and analyzing dynamic information in the evaluation and characterization of physiological responses to exercise under conditions representing altered loading states, and (ii) linking cellular alterations to the metabolic responses observed in contracting skeletal muscle. Unfortunately, limited dynamic physiological data have been collected under chronic unloading (e.g., space travel, bed rest, limb immobilization) and loading (e.g., endurance or resistance exercise) conditions at the cellular and tissue levels. Moreover, it is not clear how the chain of events involved in the adaptive process is linked to manifestations of altered protein expressions in response to altered loading states in skeletal muscle.

To predict dynamic information on the effects of chronic unloading/loading states on skeletal muscle cellular metabolic responses to constant work rate exercise, a validated physiologically-based computational model of skeletal muscle bioenergetics is required that accounts for the instantaneous increase in ATP turnover rate (acute response) and tight coupling between energy demand-energy supply systems and integrates alterations induced by chronic loading/unloading states (chronic adaptation) from the cellular to the tissue level. The objectives of the present study are therefore (i) to extend a previously developed mathematical model of skeletal muscle metabolism under normal conditions [28,29] by adding necessary biochemical elements which have been documented to be altered after periods of loading or unloading, and (ii) to predict the metabolic responses to constant work rate exercise of moderate intensity in both chronic loaded and unloaded states. Specifically, by simulating these responses under loaded, unloaded, and control states, we investigated whether feedback activation by ADP, Pi and NADH is sufficient to match ATP supply to demand at the onset of exercise or a parallel activation mechanism is required for such matching. In addition, we investigated whether specific changes in skeletal muscle enzyme content/activity induced by a chronic increase or decrease in mechanical loading can lead to alterations in the patterns of fuel oxidation during acute exercise.

## Method

### Model development

**Figure 1:**
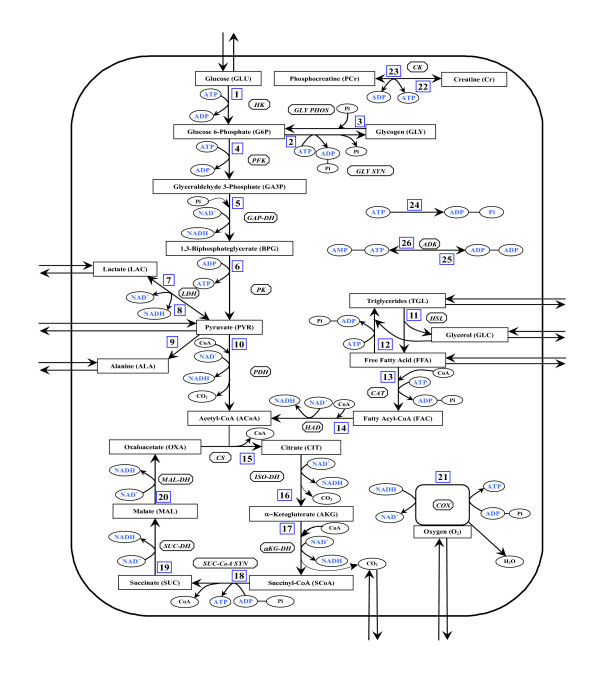
Schematic diagram of biochemical pathways depicting various chemical reactions and species involved in the cellular metabolism of skeletal muscle. The pathways involve 26 lumped reactions among 30 species out of which 9 species undergo the blood-tissue exchange.

The mathematical model of skeletal muscle metabolism is developed here by extending a previously published model [28,29], which was primarily developed in the context of whole-body metabolism. In the current model, we identified and included many more key intermediate metabolites and regulatory enzymes along the biochemical pathways (Fig 1) and redefined the metabolic reaction flux expressions following Michaelis-Menten formalism [30] to better mimic the reality of saturable enzyme kinetics. In addition, we incorporated into the model information on enzyme kinetics, which is altered after periods of chronic loading or unloading. Changes in these parameter values – from their control values representing enzyme activity/affinity in normal sedentary muscle – alter the flux expressions and mass balance equations resulting in changes in the dynamics of specific metabolites and flux rates in response to an input. A simplified map of the biochemical pathways in skeletal muscle is schematized in Figure 1. The lumped reactions in the pathways were generated by stoichiometrically coupling many elementary reactions. These include the energy controller pairs ATP-ADP and NADH-NAD^+ ^(co-substrate co-product pairs), the ratios of which modulate the associated reaction fluxes [31,32]. Many lumped reactions were considered irreversible as the corresponding regulatory enzymes *in vivo *usually have large equilibrium constants in favor of the product formation [33].

By linking changes in the work rate on a cycle ergometer to the rate of ATP turnover and by inducing parallel activating changes in several key variables (e.g., blood flow, active muscle volume, enzyme activities), we anticipate to successfully simulate exercise responses with our phenomenological model of skeletal muscle metabolism. This empirical parallel activation mechanism has also been described in other phenomenological models in the literature [29,34-37] for simulating acute exercise responses and matching ATP supply to ATP demand during exercise. Though the mechanism of parallel activation has not been proven or disproved experimentally, it is attributed to the stimulation of the activities of key regulatory enzymes by the levels of free calcium [Ca^2+^] and/or hormones (catecholamine, epinephrine) [29,34-37], which are control by neural stimulation [38-40]. In this model, the active muscle volume is expressed as a function of work rate in order to accurately simulate the extent of muscle recruitment with increased work rate [41,42]. In addition, we assume that, during exercise at work rates ranging from 30–45% VO_2 max_, the arterial substrate concentrations remain unaffected by the venous effluent due to the reestablishment of homeostasis by other organ systems such as heart, liver, or non-active muscle [29,43-45].

**Figure 2:**
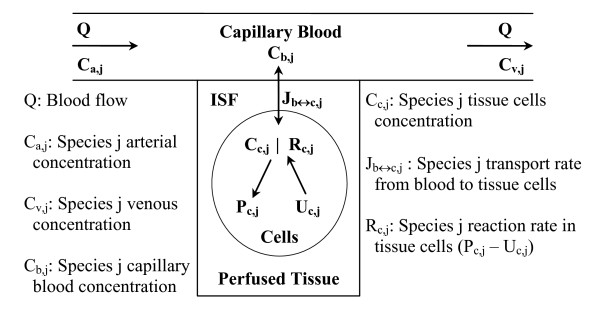
Schematic diagram of the structure of the model for blood-tissue exchange and cellular metabolism in skeletal muscle. The model accounts for 30 chemical species in tissue cells. A total of 9 species (glucose, lactate, pyruvate, alanine, triglycerides, glycerol, free fatty acid, carbon dioxide, and oxygen) undergo blood-tissue exchange.

The governing model equations are developed here by assuming a multi-compartmental model structure for skeletal muscle as schematized in Figure 2. It consists of a spatially-lumped capillary blood domain which exchanges micro-nutrients and metabolic waste products with a spatially-lumped domain of tissue cells (cytosol and mitochondria); these two domains are separated by a spatially-lumped interstitial fluid (ISF) domain. Due to lack of information on metabolite concentrations and enzyme activities at sub-cellular level, we do not differentiate between the cytosolic and mitochondrial species in this model, that is, we assume that the metabolites and enzymes are uniformly distributed throughout the cellular domain.

It is noteworthy to mention here that the capillary blood and tissue ISF volumes (V_b _and V_isf_) are very small compared to the tissue cells volume (V_c_); typically, V_b _and V_isf _are only about 7% and 13% of V_tot_, whereas V_c _is about 80% of V_tot_, where V_tot _denote the total muscle volume (e.g., see Ref. [46] and the related references therein). Furthermore, the rate of species transport across the capillary and tissue cell membranes are usually very fast (i.e., the inter-domain transport fluxes J_b↔isf,j _and J_isf↔c,j _are very large), so that the three parallel compartments can be assumed to be in equilibrium almost instantaneously. These lead to the following approximations:

*V*_*b*_(*dC*_*b*_, _*j*_/*dt*) ≪ 1   and   *V*_*isf *_(*dC*_*isf*, *j*_/*dt*) ≪ 1

*Q*(*C*_*a*, *j *_- *C*_*v*, *j*_) ≈ *J*_*b*↔*isf*, *j *_≈ *J*_*isf*↔*c*, *j *_≈ *J*_*b*↔*c*, *j*_

*C*_*b*, *j *_= *C*_*v*, *j *_= σ_*cap*, *j*_*C*_*isf*, *j *_    and     *C*_*isf*, *j *_= σ_*c*, *j*_*C*_*c*, *j*_

where C_b,j_, C_isf,j _and C_c,j _are the species concentrations in capillary blood, ISF and tissue cells; C_a,j _and C_v,j _are the input arterial and output venous species concentrations; σ_cap,j _and σ_c,j _are the partition coefficients across the capillary and tissue cell membranes; Q is the blood flow.

The dynamic mass balance equation for the chemical species j in the well-mixed tissue cells domain [28,29] can then be written as

VcdCc,jdt=∑pβj,pφp−∑uβj,uφu+Q(Ca,j−σjCc,j),j=1,2,...,NS    
 MathType@MTEF@5@5@+=feaafiart1ev1aqatCvAUfKttLearuWrP9MDH5MBPbIqV92AaeXatLxBI9gBaebbnrfifHhDYfgasaacH8akY=wiFfYdH8Gipec8Eeeu0xXdbba9frFj0=OqFfea0dXdd9vqai=hGuQ8kuc9pgc9s8qqaq=dirpe0xb9q8qiLsFr0=vr0=vr0dc8meaabaqaciaacaGaaeqabaqabeGadaaakeaafaqabeqacaaabaGaemOvay1aaSbaaSqaaiabdogaJbqabaGcdaWcaaqaaiabdsgaKjabdoeadnaaBaaaleaacqWGJbWycqGGSaalcqWGQbGAaeqaaaGcbaGaemizaqMaemiDaqhaaiabg2da9maaqafabaacciGae8NSdi2aaSbaaSqaaiabdQgaQjabcYcaSiabdchaWbqabaGccqWFgpGzdaWgaaWcbaGaemiCaahabeaakiabgkHiTaWcbaGaemiCaahabeqdcqGHris5aOWaaabuaeaacqWFYoGydaWgaaWcbaGaemOAaOMaeiilaWIaemyDauhabeaakiab=z8aMnaaBaaaleaacqWG1bqDaeqaaaqaaiabdwha1bqab0GaeyyeIuoakiabgUcaRiabdgfarnaabmaabaGaem4qam0aaSbaaSqaaiabdggaHjabcYcaSiabdQgaQbqabaGccqGHsislcqWFdpWCdaWgaaWcbaGaemOAaOgabeaakiabdoeadnaaBaaaleaacqWGJbWycqGGSaalcqWGQbGAaeqaaaGccaGLOaGaayzkaaGaeiilaWcabaGaemOAaOMaeyypa0JaeGymaeJaeiilaWIaeGOmaiJaeiilaWIaeiOla4IaeiOla4IaeiOla4IaeiilaWIaemOta40aaSbaaSqaaiabdofatbqabaaaaOGaeeiiaaIaeeiiaaIaeeiiaaIaeeiiaacaaa@74BB@

where σ_j _= σ_cap,j_σ_c,j _is the effective partition coefficient across the blood and tissue cell exchange barrier; φ_p _and φ_u _are the metabolic reaction fluxes of the reactions processes that produce and utilize the species j, and β_j,p _and β_j,u _are the corresponding stoichiometric coefficients; N_S _= 30 is the total number of chemical species in the cellular domain. The first two terms in Eq. (2) define the net metabolic reaction rate of the chemical species j inside the tissue cells (R_j _= P_j _- U_j_, where P_j _is the production of species j and U_j _is the utilization of species j), whereas the term Q(C_a,j _- σ_j_C_c,j_) defines the net uptake or release of the chemical species j by the tissue cells. The dynamic mass balance equations of all the 30 chemical species are given in Appendix A.

The underlying viewpoint in modeling complex *in vivo *metabolic systems, such as skeletal muscle, is based on a top-down systems approach [28,29,32,47,48]. To define the metabolic reaction fluxes using such an approach, we consider a general irreversible bi-bi substrate to product enzymatic reaction coupled with energy controller pairs associated with the reaction as co-substrates and co-products:



where E_1 _and E_2 _are the ATP and ADP or vice-versa (PS±: phosphorylation state) or NADH and NAD^+ ^or vice versa (RS±: redox state). Any biochemical conversion process that is coupled to ATP ↔ ADP or NAD^+ ^↔ NADH interconversions, such as pyruvate oxidation or oxidative phosphorylation, can affect the dynamics of these control metabolites. So, even though E_1 _and E_2 _are co-substrates and co-products, their ratios have been proven to play major roles in the modulation of the associated reaction fluxes [28,29,32,47,48] (see the flux expressions: Eqs. 4 and 5). Though not all, many of the lumped reactions in the metabolic pathways in Figure 1 are special cases of this general reaction. As part of a general formalism, reversible reactions are decomposed into two irreversible reactions. Considering Michaelis-Menten formalism of a rapid equilibrium, random binding, bi-bi enzymatic irreversible reaction [30,49,50], and accounting for product inhibition [31], the reaction flux can be phenomenologically expressed as

φX+Y→V+W=VX+Y→V+Weffective[CXKXCYKY][1+CXKX+CYKY+CXKXCYKY+CVKV+CWKW+CVKVCWKW]−1
 MathType@MTEF@5@5@+=feaafiart1ev1aaatCvAUfKttLearuWrP9MDH5MBPbIqV92AaeXatLxBI9gBaebbnrfifHhDYfgasaacH8akY=wiFfYdH8Gipec8Eeeu0xXdbba9frFj0=OqFfea0dXdd9vqai=hGuQ8kuc9pgc9s8qqaq=dirpe0xb9q8qiLsFr0=vr0=vr0dc8meaabaqaciaacaGaaeqabaqabeGadaaakeaaiiGacqWFgpGzdaWgaaWcbaGaemiwaGLaey4kaSIaemywaKLaeyOKH4QaemOvayLaey4kaSIaem4vaCfabeaakiabg2da9iabdAfawnaaDaaaleaacqWGybawcqGHRaWkcqWGzbqwcqGHsgIRcqWGwbGvcqGHRaWkcqWGxbWvaeaacqqGLbqzcqqGMbGzcqqGMbGzcqqGLbqzcqqGJbWycqqG0baDcqqGPbqAcqqG2bGDcqqGLbqzaaGcdaWadaqaamaalaaabaGaem4qam0aaSbaaSqaaiabdIfaybqabaaakeaacqWGlbWsdaWgaaWcbaGaemiwaGfabeaaaaGcdaWcaaqaaiabdoeadnaaBaaaleaacqWGzbqwaeqaaaGcbaGaem4saS0aaSbaaSqaaiabdMfazbqabaaaaaGccaGLBbGaayzxaaWaamWaaeaacqaIXaqmcqGHRaWkdaWcaaqaaiabdoeadnaaBaaaleaacqWGybawaeqaaaGcbaGaem4saS0aaSbaaSqaaiabdIfaybqabaaaaOGaey4kaSYaaSaaaeaacqWGdbWqdaWgaaWcbaGaemywaKfabeaaaOqaaiabdUealnaaBaaaleaacqWGzbqwaeqaaaaakiabgUcaRmaalaaabaGaem4qam0aaSbaaSqaaiabdIfaybqabaaakeaacqWGlbWsdaWgaaWcbaGaemiwaGfabeaaaaGcdaWcaaqaaiabdoeadnaaBaaaleaacqWGzbqwaeqaaaGcbaGaem4saS0aaSbaaSqaaiabdMfazbqabaaaaOGaey4kaSYaaSaaaeaacqWGdbWqdaWgaaWcbaGaemOvayfabeaaaOqaaiabdUealnaaBaaaleaacqWGwbGvaeqaaaaakiabgUcaRmaalaaabaGaem4qam0aaSbaaSqaaiabdEfaxbqabaaakeaacqWGlbWsdaWgaaWcbaGaem4vaCfabeaaaaGccqGHRaWkdaWcaaqaaiabdoeadnaaBaaaleaacqWGwbGvaeqaaaGcbaGaem4saS0aaSbaaSqaaiabdAfawbqabaaaaOWaaSaaaeaacqWGdbWqdaWgaaWcbaGaem4vaCfabeaaaOqaaiabdUealnaaBaaaleaacqWGxbWvaeqaaaaaaOGaay5waiaaw2faamaaCaaaleqabaGaeyOeI0IaeGymaedaaaaa@8D8D@

where the effective reaction velocity due to the coupling of the energy controller pairs is given by a phenomenological expression of the form

VX+Y→V+Weffective=VX+Y→V+W[CE1/CE2][KE1/E2+CE1/CE2]−1
 MathType@MTEF@5@5@+=feaafiart1ev1aaatCvAUfKttLearuWrP9MDH5MBPbIqV92AaeXatLxBI9gBaebbnrfifHhDYfgasaacH8akY=wiFfYdH8Gipec8Eeeu0xXdbba9frFj0=OqFfea0dXdd9vqai=hGuQ8kuc9pgc9s8qqaq=dirpe0xb9q8qiLsFr0=vr0=vr0dc8meaabaqaciaacaGaaeqabaqabeGadaaakeaacqWGwbGvdaqhaaWcbaGaemiwaGLaey4kaSIaemywaKLaeyOKH4QaemOvayLaey4kaSIaem4vaCfabaGaeeyzauMaeeOzayMaeeOzayMaeeyzauMaee4yamMaeeiDaqNaeeyAaKMaeeODayNaeeyzaugaaOGaeyypa0JaemOvay1aaSbaaSqaaiabdIfayjabgUcaRiabdMfazjabgkziUkabdAfawjabgUcaRiabdEfaxbqabaGcdaWadaqaamaalyaabaGaem4qam0aaSbaaSqaaiabdweafnaaBaaameaacqaIXaqmaeqaaaWcbeaaaOqaaiabdoeadnaaBaaaleaacqWGfbqrdaWgaaadbaGaeGOmaidabeaaaSqabaaaaaGccaGLBbGaayzxaaWaamWaaeaacqWGlbWsdaWgaaWcbaWaaSGbaeaacqWGfbqrdaWgaaadbaGaeGymaedabeaaaSqaaiabdweafnaaBaaameaacqaIYaGmaeqaaaaaaSqabaGccqGHRaWkdaWcgaqaaiabdoeadnaaBaaaleaacqWGfbqrdaWgaaadbaGaeGymaedabeaaaSqabaaakeaacqWGdbWqdaWgaaWcbaGaemyrau0aaSbaaWqaaiabikdaYaqabaaaleqaaaaaaOGaay5waiaaw2faamaaCaaaleqabaGaeyOeI0IaeGymaedaaaaa@68AF@

V_X+Y→V+W _is the maximal velocity or flux of the reaction and K's are the phenomenological Michaelis-Menten constants for the associated reactants or the coupled energy controller ratios E_1_/E_2_: ATP/ADP, ADP/ATP, NADH/NAD^+^, or NAD^+^/NADH. So, the *in vivo *effective velocity of the reaction is controlled by the coupled energy controller ratios C_E1_/C_E2 _(if coupled). As in our previous models [28,29,47,48], we choose to express controllers in ratio form to increase the sensitivity of their regulatory properties on actual *in vivo *reaction velocities. While AMP is not directly involved in any reaction outside of the adenylate kinase system, we coupled the energy controller ratio C_AMP_/C_ATP _to the reaction flux describing glycogen breakdown, since it is well-known that AMP has important allosteric control over the enzymes catalyzing the above lumped reaction [49-51]. Even though the reaction flux expressions are "phenomenological", expressing the fluxes in the Michaelis-Menten form serves two purposes: (i) it best describes the kinetic behavior of enzymatic reactions seen experimentally, and (ii) it establishes upper and lower bounds for the fluxes [30].

It is to be noted here that Eqs. (4) and (5) serve as the general flux expression for the lumped metabolic reaction (3). If the energy controller pair E_1 _and E_2 _is not coupled to the reaction under consideration as co-substrate/co-product, then the reaction flux does not depend on the ratio C_E1_/C_E2_. Therefore, not all, but only those reactions stoichiometrically associated with changes in phosphorylation (C_ATP_/C_ADP _or C_ADP_/C_ATP_) and/or redox (C_NADH_/C_NAD+ _or C_NAD+_/C_NADH_) ratios have their flux rate modulated by these controllers. The phenomenological metabolic reaction flux expressions of all the 26 lumped reactions considered in this model are given in Appendix B.

As modeled, a biochemical conversion process coupled to NAD^+ ^↔ NADH interconversions affects the redox state C_NAD+_/C_NADH _and indirectly modulates the flux of any other reaction process coupled to NAD^+ ^↔ NADH interconversions. Similar interrelationships exist among all processes coupled to ATP ↔ ADP interconversions. In the control of energy metabolism, ATP acts as an inhibitor, while ADP acts as a stimulator. This approach of modeling the regulation of metabolic processes provides not only a feedback control of specific conversion processes, but also integrates various metabolic pathways for energy (ATP) generation. As described below, the feedback controls by the products ADP, Pi and NADH may not alone be sufficient to match ATP supply to ATP demand during exercise, in which case, a parallel activation of different metabolic pathways is required for such matching.

### Parallel activation scheme during exercise

The rate at which ATP is utilized is set by the increase in mechanical power needed to perform a particular physical task. During exercise at a constant work rate, a proportionate recruitment of muscle fibers occurs, and steady contraction can be maintained only if the ATP demand is matched. As the ATP turnover rate increases, flux through the energy-providing pathways of carbohydrates and fats rises to increase the rate of ATP production in order to match the rate of ATP utilization [8,52]. This increase in the ATP turnover rate is met through (i) increased delivery of arterial species by increased blood flow and (ii) feedback and/or feedforward activation of latent enzyme molecules associated with various reactions. The feedback control mechanisms (though not mechanistically detailed) are built-in into the flux expressions through phosphorylation (C_ATP_/C_ADP _or C_ADP_/C_ATP_) and/or redox (C_NADH_/C_NAD+ _or C_NAD+_/C_NADH_) ratios. So we discuss here and incorporate into the model the feedforward control mechanisms in an empirical/implicit fashion to simultaneously activate different metabolic pathways leading to ATP generation (glycolysis and glycogenolysis, pyruvate oxidation, beta oxidation, TCA cycle, and oxidative phosphorylation) through external effectors such as [Ca^2+^] and/or hormones.

Initiation of an exercise bout is implemented here by increasing the rate of ATP hydrolysis in a stepwise manner [29] to a value proportional to the effective external work rate (WR + WR_unload_) applied by the cycle ergometer:

φ_*ATP*→*ADP*_(*WR*) = φ_*ATP*→*ADP*, *rest *_+ γ_1_[*WR *+ *WR*_*unload*_]

where Φ_ATP→ADP,rest _is the resting muscle ATP turnover rate, γ_1 _is the conversion factor relating the effective external work rate to the units of ATP hydrolysis, and WR_unload _≈ 25W is the WR equivalent of unloaded cycling. Once the desired WR has been selected, ATP utilization rate increases and parallel changes occur in several physiological variables.

The ATP demand is primarily met by the recruitment of motor units. At rest, there is a minimal level of contraction occurring throughout various areas of skeletal muscle tissue [41,42]. Due to the fact that not all muscle is engaged simultaneously over the period of time, we assume that in the resting state, the volume of active muscle is only a fraction of the total muscle volume. During exercise, the amount of muscle mass actively participating in muscular contraction is linearly related to the work rate [41,42]. Therefore, in our model, the active muscle tissue cells volume V_c _is expressed in terms of the work rate (WR) as:

*V*_c_(*WR*) = *V*_*c*, *rest *_+ γ_2_[*WR *+ *WR*_*unload*_]

where γ_2 _is the slope ΔV_c_/Δ(WR+WR_unload_) and V_c,rest _is the active muscle tissue cells volume at rest; γ_2 _can be different for different loading conditions. In contrast, the skeletal muscle blood flow response to a step change in work rate (WR) is expressed as [53]:

*Q*(*t*, *WR*) = *Q*_*rest *_+ γ_3_[*WR *+ *WR*_*unload*_][1 - exp^-*t*/τ^]

where γ_3 _is the slope ΔQ(t = ∞)/Δ(WR+WR_unload_), τ is the time constant of response, and Q_rest _the resting skeletal muscle blood flow; τ can be different for different muscle conditions, while γ_3 _can be assumed constant, as the steady-state blood flow rate during exercise can be independent of the muscle condition.

To account for a tight coupling of energy demand-energy supply pathways that is known to occur in exercising muscle, the metabolic reaction fluxes were expressed in terms of the work rate (WR). Anticipating that the feedback controls by ADP, Pi and NADH (through cofactors C_ATP_/C_ADP _or C_ADP_/C_ATP _and C_NADH_/C_NAD+ _or C_NAD+_/C_NADH_) may not alone be enough to achieve the tight coupling of ATP supply-ATP demand systems during exercise, we chose to upregulate the effective enzyme activity for each reaction by multiplying each V_max _in the flux expressions by the relative metabolic rate (RMR):

RMR=MRrest+1η[WR+WRunload]MRrest
 MathType@MTEF@5@5@+=feaafiart1ev1aaatCvAUfKttLearuWrP9MDH5MBPbIqV92AaeXatLxBI9gBaebbnrfifHhDYfgasaacH8akY=wiFfYdH8Gipec8Eeeu0xXdbba9frFj0=OqFfea0dXdd9vqai=hGuQ8kuc9pgc9s8qqaq=dirpe0xb9q8qiLsFr0=vr0=vr0dc8meaabaqaciaacaGaaeqabaqabeGadaaakeaajaaycqWGsbGucqWGnbqtcqWGsbGucqGH9aqpkmaalaaajaaybaGaemyta0KaemOuaiLcdaWgaaqcbawaaiabdkhaYjabdwgaLjabdohaZjabdsha0bqabaqcaaMaey4kaSIcdaWcbaqcbawaaiabigdaXaqaaGGaciab=D7aObaakmaadmaajaaybaGaem4vaCLaemOuaiLaey4kaSIaem4vaCLaemOuaiLcdaWgaaqcbawaaiabdwha1jabd6gaUjabdYgaSjabd+gaVjabdggaHjabdsgaKbqabaaajaaycaGLBbGaayzxaaaabaGaemyta0KaemOuaiLcdaWgaaqcbawaaiabdkhaYjabdwgaLjabdohaZjabdsha0bqabaaaaaaa@58E1@

where η is the mechanical coupling efficiency [29,54] and MR_rest _the resting metabolic rate; η converts WR from the units of mechanical to metabolic watts. If no external work is performed, RMR = 1 and the V_max _values remain unchanged. To implement a feedforward control during exercise, the effective enzyme activities – characterized by the V_max _parameters- of the energy producing pathways are increased in proportion to the relative increase in metabolic rate [29,34,35,37]. This way, the metabolic reaction fluxes are implicitly expressed in terms of the ATP turnover rate through the variable WR. The model physiological parameters in Eqs. (6) – (9) are given in Table 1.

**Table 1:** The values of model physiological parameters characterizing the moderate intensity exercise conditions (30–45% VO_2 max_, WR = 65W) for different loading states (if available)

*Parameter*	*Value*	*Unit*	*Equation*	*Reference*
V_c,rest_, Vc,restLD MathType@MTEF@5@5@+=feaafiart1ev1aaatCvAUfKttLearuWrP9MDH5MBPbIqV92AaeXatLxBI9gBaebbnrfifHhDYfgasaacH8akY=wiFfYdH8Gipec8Eeeu0xXdbba9frFj0=OqFfea0dXdd9vqai=hGuQ8kuc9pgc9s8qqaq=dirpe0xb9q8qiLsFr0=vr0=vr0dc8meaabaqaciaacaGaaeqabaqabeGadaaakeaacqWGwbGvdaqhaaWcbaGaee4yamMaeeilaWIaeeOCaiNaeeyzauMaee4CamNaeeiDaqhabaGaeeitaWKaeeiraqeaaaaa@3800@	4.0	L	7	[42,78]
Vc,restUL MathType@MTEF@5@5@+=feaafiart1ev1aaatCvAUfKttLearuWrP9MDH5MBPbIqV92AaeXatLxBI9gBaebbnrfifHhDYfgasaacH8akY=wiFfYdH8Gipec8Eeeu0xXdbba9frFj0=OqFfea0dXdd9vqai=hGuQ8kuc9pgc9s8qqaq=dirpe0xb9q8qiLsFr0=vr0=vr0dc8meaabaqaciaacaGaaeqabaqabeGadaaakeaacqWGwbGvdaqhaaWcbaGaee4yamMaeeilaWIaeeOCaiNaeeyzauMaee4CamNaeeiDaqhabaGaeeyvauLaeeitaWeaaaaa@3822@	3.5	L	7	[21]
γ_1_, γ1LD MathType@MTEF@5@5@+=feaafiart1ev1aaatCvAUfKttLearuWrP9MDH5MBPbIqV92AaeXatLxBI9gBaebbnrfifHhDYfgasaacH8akY=wiFfYdH8Gipec8Eeeu0xXdbba9frFj0=OqFfea0dXdd9vqai=hGuQ8kuc9pgc9s8qqaq=dirpe0xb9q8qiLsFr0=vr0=vr0dc8meaabaqaciaacaGaaeqabaqabeGadaaakeaaiiGacqWFZoWzdaqhaaWcbaGaeGymaedabaGaeeitaWKaeeiraqeaaaaa@31A5@, γ1UL MathType@MTEF@5@5@+=feaafiart1ev1aaatCvAUfKttLearuWrP9MDH5MBPbIqV92AaeXatLxBI9gBaebbnrfifHhDYfgasaacH8akY=wiFfYdH8Gipec8Eeeu0xXdbba9frFj0=OqFfea0dXdd9vqai=hGuQ8kuc9pgc9s8qqaq=dirpe0xb9q8qiLsFr0=vr0=vr0dc8meaabaqaciaacaGaaeqabaqabeGadaaakeaaiiGacqWFZoWzdaqhaaWcbaGaeGymaedabaGaeeyvauLaeeitaWeaaaaa@31C7@	2.25	mmol/(min × W)	6	[29]
γ_2_, γ2LD MathType@MTEF@5@5@+=feaafiart1ev1aaatCvAUfKttLearuWrP9MDH5MBPbIqV92AaeXatLxBI9gBaebbnrfifHhDYfgasaacH8akY=wiFfYdH8Gipec8Eeeu0xXdbba9frFj0=OqFfea0dXdd9vqai=hGuQ8kuc9pgc9s8qqaq=dirpe0xb9q8qiLsFr0=vr0=vr0dc8meaabaqaciaacaGaaeqabaqabeGadaaakeaaiiGacqWFZoWzdaqhaaWcbaGaeGOmaidabaGaeeitaWKaeeiraqeaaaaa@31A7@	0.0375	L/W	7	[42,78]
γ2UL MathType@MTEF@5@5@+=feaafiart1ev1aaatCvAUfKttLearuWrP9MDH5MBPbIqV92AaeXatLxBI9gBaebbnrfifHhDYfgasaacH8akY=wiFfYdH8Gipec8Eeeu0xXdbba9frFj0=OqFfea0dXdd9vqai=hGuQ8kuc9pgc9s8qqaq=dirpe0xb9q8qiLsFr0=vr0=vr0dc8meaabaqaciaacaGaaeqabaqabeGadaaakeaaiiGacqWFZoWzdaqhaaWcbaGaeGOmaidabaGaeeyvauLaeeitaWeaaaaa@31C9@	0.043	L/W	7	[21]
γ_3_, γ3LD MathType@MTEF@5@5@+=feaafiart1ev1aaatCvAUfKttLearuWrP9MDH5MBPbIqV92AaeXatLxBI9gBaebbnrfifHhDYfgasaacH8akY=wiFfYdH8Gipec8Eeeu0xXdbba9frFj0=OqFfea0dXdd9vqai=hGuQ8kuc9pgc9s8qqaq=dirpe0xb9q8qiLsFr0=vr0=vr0dc8meaabaqaciaacaGaaeqabaqabeGadaaakeaaiiGacqWFZoWzdaqhaaWcbaGaeG4mamdabaGaeeitaWKaeeiraqeaaaaa@31A9@, γ3UL MathType@MTEF@5@5@+=feaafiart1ev1aaatCvAUfKttLearuWrP9MDH5MBPbIqV92AaeXatLxBI9gBaebbnrfifHhDYfgasaacH8akY=wiFfYdH8Gipec8Eeeu0xXdbba9frFj0=OqFfea0dXdd9vqai=hGuQ8kuc9pgc9s8qqaq=dirpe0xb9q8qiLsFr0=vr0=vr0dc8meaabaqaciaacaGaaeqabaqabeGadaaakeaaiiGacqWFZoWzdaqhaaWcbaGaeG4mamdabaGaeeyvauLaeeitaWeaaaaa@31CB@	0.0575	L/(min × W)	8	[29,70]
*Q*_rest_	0.9	L/min	8	[45]
τ_Q_	0.35	min	8	[53]
τQLD MathType@MTEF@5@5@+=feaafiart1ev1aaatCvAUfKttLearuWrP9MDH5MBPbIqV92AaeXatLxBI9gBaebbnrfifHhDYfgasaacH8akY=wiFfYdH8Gipec8Eeeu0xXdbba9frFj0=OqFfea0dXdd9vqai=hGuQ8kuc9pgc9s8qqaq=dirpe0xb9q8qiLsFr0=vr0=vr0dc8meaabaqaciaacaGaaeqabaqabeGadaaakeaaiiGacqWFepaDdaqhaaWcbaGaemyuaefabaGaeeitaWKaeeiraqeaaaaa@31FE@	0.25	min	8	[53]
τQUL MathType@MTEF@5@5@+=feaafiart1ev1aaatCvAUfKttLearuWrP9MDH5MBPbIqV92AaeXatLxBI9gBaebbnrfifHhDYfgasaacH8akY=wiFfYdH8Gipec8Eeeu0xXdbba9frFj0=OqFfea0dXdd9vqai=hGuQ8kuc9pgc9s8qqaq=dirpe0xb9q8qiLsFr0=vr0=vr0dc8meaabaqaciaacaGaaeqabaqabeGadaaakeaaiiGacqWFepaDdaqhaaWcbaGaemyuaefabaGaeeyvauLaeeitaWeaaaaa@3220@	0.45	min	8	[79]
η	0.5	unitless	9	[54,71]
*MR*_rest_	20.6	W	9	[71]
*WR*	65.0	W	6, 7, 8, 9	[29]
*WR*_unload_	25.0	W	6, 7, 8, 9	[29]
*RQ*	0.785	unitless	*V*_*co*2_/*V*_*O*2_	[67,68]

### Parameter estimation and model validation

The model kinetic parameters for a normal sedentary individual were obtained and the model was validated by comparing model simulation results with experimental data at (i) rest, (ii) during severe ischemia, and (iii) during moderate-intensity exercise. Most initial parameter estimates for the loaded and unloaded states were obtained from the literature. Parameter values were then tuned manually by comparing model simulated dynamic responses to moderate-intensity exercise with available experimental data. The model consists of a system of nonlinear ordinary differential and algebraic equations (Appendix A and B), which are solved numerically as an initial value problem with a well-developed FORTRAN implicit integrator DLSODES (Livermore Solver for Ordinary Differential Equations) [55].

Information on skeletal muscle metabolism in humans was gathered from various studies on sedentary healthy individuals [43,51,56-61]. Information corresponding to the chronically-loaded muscle was obtained from (i) intervention studies where physiological parameters were evaluated in sedentary subjects who took part in endurance-training programs [6,12,13,15,62] and (ii) related studies that compared sedentary and trained individuals [16,18,20]. In regards to the chronic unloading muscle, most available data was obtained from (i) bed rest studies ranging from 1–4 weeks [21,23,63] and (ii) detraining studies in athletes lasting 4–12 weeks [8,22,24,64]. Values for arterial and venous species concentrations and muscle blood flow were obtained from the femoral bed in human studies [12,43,65,66], while the values for intracellular species concentrations were obtained from muscle biopsies of the quadriceps [6,56,57]. Most substrate concentrations were the same at rest for all loading states with the exception of intramuscular triglyceride and glycogen concentrations and several metabolite concentrations in arterial blood. Tables 2 and 3 list arterial and intracellular concentrations, respectively, of all included species in each state. Blood-tissue partition coefficients were estimated from the blood-tissue exchange term (uptake-release rates). The coefficients for the nine species that are either taken up or released by skeletal muscle are displayed in Table 2.

**Table 2:** Arterial species concentrations (mM) and blood-tissue partition coefficients (unitless) estimated from resting conditions for three different loading states.

*Species*	*Arterial Species Concentrations*			*Blood-Tissue Partition Coefficients*		
	
	*Sedentary*	*Loaded*	*Unloaded*	*Sedentary*	*Loaded*	*Unloaded*
GLU	4.2	4.2	4.2	7.62	7.62	7.62
PYR	0.068	0.068	0.068	1.3	1.3	1.3
LAC	0.7	0.7	0.7	0.471	0.471	0.471
ALA	0.192	0.192	0.24	0.182	0.182	0.219
TGL	0.99	0.86	1.26	0.0667	0.0492	0.0679
GLC	0.06	0.06	0.04	1.02	1.02	0.7
FFA	0.59	0.59	0.43	0.867	0.867	0.587
CO2	21.7	21.7	21.7	1.02	1.02	1.02
O2	8.0	8.0	8.0	1.65	1.65	1.65

The species concentrations are obtained from a variety literature sources on skeletal muscle metabolism (for references, see the text in Method section). The partition coefficients σ is calculated from the formula C_b,j _= C_v,j _= σ_j _C_c,j_, where C_b,j_, C_v,j _and C_c,j _are the blood, venous and tissue cells species concentrations

**Table 3:** Muscle tissue cells species concentrations at rest for the three different loading states (concentrations are in the units of mM).

*Species*	*Sedentary*	*Loaded*	*Unloaded*
GLU	0.525	0.525	0.525
GLY	95.0	110.0	110.0
G6P	0.253	0.253	0.253
GA3P	0.08	0.08	0.08
BPG	0.08	0.08	0.08
PYR	0.0475	0.0475	0.0475
LAC	1.75	1.75	1.75
ALA	1.3	1.3	1.3
TGL	14.8	17.4	18.5
GLC	0.062	0.062	0.062
FFA	0.57	0.57	0.57
FAC	0.00348	0.00348	0.00348
ACoA	0.00223	0.00223	0.00223
CIT	0.103	0.103	0.103
AKG	0.0125	0.0125	0.0125
SCoA	0.123	0.123	0.123
SUC	0.095	0.095	0.095
MAL	0.0975	0.0975	0.0975
OXA	0.003	0.003	0.003
CO2	23.6	23.6	23.6
O2	3.0	3.0	3.0
PCr	20.0	20.0	20.0
Cr	10.0	10.0	10.0
Pi	2.7	2.7	2.7
CoA	0.0255	0.0255	0.0255
NADH	0.05	0.05	0.05
NAD+	0.45	0.45	0.45
ATP	6.3	6.3	6.3
ADP (free)	0.02	0.02	0.02
AMP (free)	6.0E-5	6.0E-5	6.0E-5

The species concentrations are obtained from a variety literature sources on skeletal muscle metabolism (for references, see the text in Method section)

Flux balance analysis is done to determine the reaction flux rates at resting steady states. In the first step, we estimated the relative contribution of carbohydrates and fats as fuels from the measurements of respiratory quotient (RQ) across the quadriceps muscle at rest [67,68]. Then, we determined the rate of CO_2 _production at rest from the known values of muscle VO_2 _at rest in combination with resting RQ value. Assuming carbohydrates (CHO, RQ_CHO _= 1.0) and fats (FFA, RQ_FFA _= 0.7) are the only fuels, we calculated the relative contributions from CHO and FFA towards the muscle CO_2 _production under resting, steady-state conditions. Then, we determined the reaction flux rates through the oxidative pathways of both CHO and FFA using appropriate stoichiometric relationships. The information derived from tracer studies was used to determine the flux rates of the forward- and reverse-contributions of reversible reactions such as the lactate dehydrogenase system and glycogen breakdown and synthesis systems [13]. The estimated reaction flux rates are displayed in Table 4.

**Table 4:** Metabolic reaction fluxes (mmol/min) estimated under resting steady-state conditions from flux balance analysis and reaction maximal velocities (mmol/min) estimated under resting steady-state conditions from reaction fluxes for three different loading states.

*Reaction Fluxes*	*Resting Flux Values*	*Maximal Velocities*	*Sedentary*	*Loading*	*Unloading*
φ_GLU→G6P_	0.182	*V*_GLY→G6P_	0.46	0.557	0.557
φ_G6P→GLY_	0.258	*V*_G6P→GLY_	2.58	3.42	2.58
φ_GLY→G6P_	0.258	*V*_GLY→G6P_	1.72	1.72	2.08
φ_G6P→GA3P_	0.182	*V*_G6P→GA3P_	1.63	1.45	1.91
φ_GA3P→BPG_	0.363	*V*_GA3P→BPG_	48.0	48.0	48.0
φ_BPG→PYR_	0.363	*V*_BPG→PYR_	25.4	25.4	25.4
φ_PYR→LAC_	0.294	*V*_PYR→LAC_	8.81	6.86	8.81
φ_LAC→PYR_	0.182	*V*_LAC→PYR_	1.09	0.85	1.09
φ_PYR→ALA_	0.04	*V*_PYR→ALA_	0.12	0.12	0.16
φ_PYR→ACoA_	0.217	*V*_PYR→ACoA_	15.2	15.2	15.2
φ_TGL→GLC_	0.13	*V*_TGL→GLC_	0.65	0.823	0.65
φ_GLC→TGL_	0.127	*V*_GLC→TGL_	1.78	2.31	1.78
φ_FFA→FAC_	0.095	*V*_FFA→FAC_	1.33	1.73	1.06
φ_FAC→ACoA_	0.095	*V*_FAC→ACoA_	0.951	1.2	0.768
φ_ACoA→CIT_	0.978	*V*_ACoA→CIT_	6.84	9.54	5.71
φ_CIT→AKG_	0.978	*V*_CIT→AKG_	9.78	14.2	8.22
φ_AKG→SCoA_	0.978	*V*_AKG→SCoA_	13.7	19.1	11.4
φ_SCoA→SUC_	0.978	*V*_SCoA→SUC_	13.7	19.1	11.4
φ_SUC→MAL_	0.978	*V*_SUC→MAL_	5.87	8.8	4.89
φ_MAL→OXA_	0.978	*V*_MAL→OXA_	5.87	8.8	4.89
φ_O2→H2O_	2.75	*V*_O2→H2O_	24.6	35.2	20.3
φ_PCR→CR_	667.0	*V*_PCR→CR_	4000.0	4000.0	4000.0
φ_CR→PCR_	667.0	*V*_CR→PCR_	4000.0	4000.0	4000.0
φ_ATP→ADP_	16.0	*V*_ATP→ADP_	16.2	16.2	16.2
φ_AMP→ADP_	50.0	*V*_AMP→ADP_	250.0	250.0	250.0
φ_ADP→AMP_	50.0	*V*_ADP→AMP_	250.0	250.0	250.0

The maximal velocities differ between different muscle states due to different K_m _values

**Figure 3:**
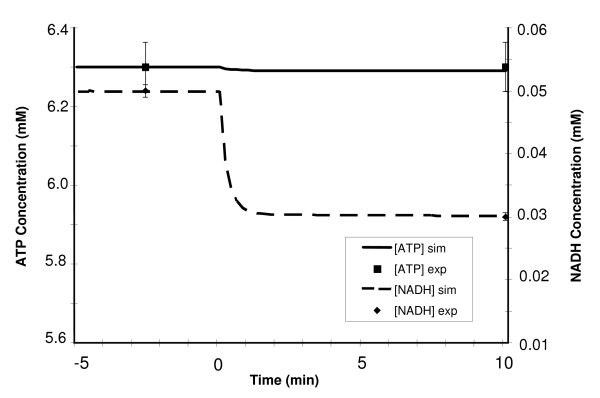
Dynamic responses of ATP and NADH concentrations during a 10-min moderate intensity exercise period (WR = 65W) of a sedentary individual. The left y-axis corresponds to changes in [ATP] while the right y-axis indicates the changes in [NADH]. Steady state responses are present from time -5 min to time 0 min when the step change in work rate (WR) is initiated. The solid and dashed lines represent the model simulation results with the symbols representing experimental data points [mean ± SD] [60,69]. [ATP] remains fairly constant while [NADH] drops 40% during exercise.

**Figure 4:**
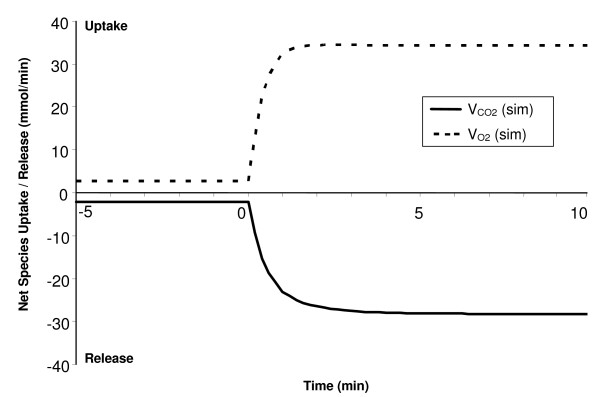
Simulated dynamic responses of oxygen uptake and carbon dioxide release during a 10-min moderate-intensity exercise bout (WR = 65W) of a sedentary individual. Positive values correspond to O_2 _uptake (VO_2 _= Q(C_a,O2 _- σ_O2_C_c,O2_)) while negative values correspond to CO_2 _release (VCO_2 _= Q(C_a,CO2 _- σ_CO2_C_c,CO2_)) from skeletal muscle. The time constant of O_2 _uptake (τ_VO2_) is about 0.5 min and that of CO_2 _release (τ_VCO2_) is about 0.6 min. The respiratory quotient (RQ = VCO_2_/VO_2_) increases from 0.785 at rest to 0.832 during exercise.

**Figure 5:**
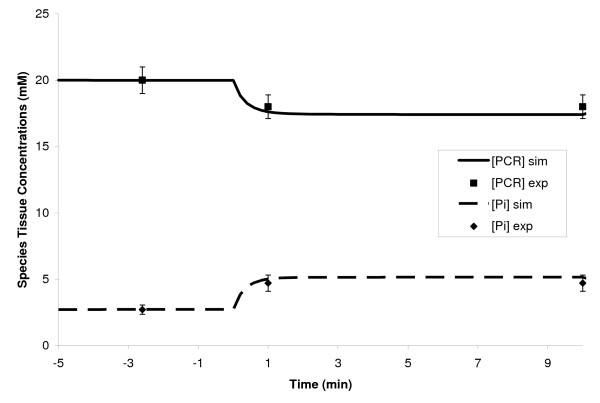
Dynamic concentration profiles of phosphocreatine (PCr) and inorganic phosphate (Pi) during a 10-min moderate-intensity exercise period (WR = 65W) of a sedentary individual. The steady state responses are present from time -5 min to time 0 min when the step change in work rate (WR) is initiated. The solid and dashed lines represent the model simulation results with the symbols representing experimental data points [mean ± SD] [65]. The time constants of both [PCr] drop and [Pi] rise are approximately equal (τ_PCr _≈ τ_Pi _≈ 0.5 min). The dynamics of [PCr] match very well with the dynamics of muscle VO_2 _[72-74].

**Figure 6:**
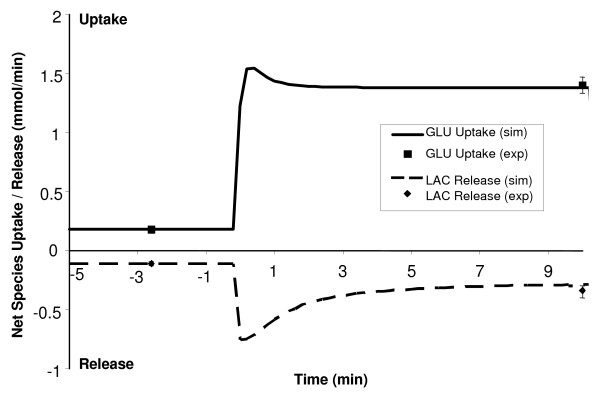
Simulated dynamic responses of glucose uptake and lactate release during a 10-min moderate-intensity exercise bout (WR = 65W) of a sedentary person. Positive values correspond to GLU uptake while negative values correspond to LAC release from skeletal muscle. The solid and dashed lines signify the model simulation results with the symbols representing experimental data points [mean ± SD] [12,13,43,45]. Both glucose uptake and lactate release have the biphasic behavior/transients.

The kinetic parameters (V_max _and K_m_) were obtained from information available on the specific enzyme kinetics. For enzymes catalyzing oxidative phosphorylation, hexokinase, and ATP hydrolysis, the K_m_'s were set to values much smaller than the corresponding intracellular reactants concentrations because of their high affinity for the respective reactants [9,30,34]. The remaining K_m_'s were assumed to have values very close to those of the corresponding reactants [30]. After setting the K_m _values, the V_max _values were estimated from known flux rates from flux balance analysis. Fine tuning of several kinetic parameters was needed by comparing model responses with experimental data during different physiological conditions, particularly during severe muscle ischemia [58,61] and moderate-intensity exercise [12,13,43,45,60,65,69] (e.g., see Figures 3, 4, 5, 6). Since the number of unknown model parameters was too large, without enough bound/physiological/thermodynamic constraints, a formal parameter estimation algorithm would provide non-unique estimations. Therefore, the model parameters were not estimated through optimization. Instead, only those parameters, which are directly linked to the measured output variables were estimated manually through an *ad hoc *trial-and-error method. For example, the K_m _values associated with the flux expression of the hexokinase reaction would directly influence the concentrations of glucose (GLU) and glucose-6-phosphate (G6P), and therefore for fitting the model to the data on [GLU] and [G6P], those K_m _parameters need to be tuned first. Though a formal parameter sensitivity analysis would be helpful in identifying the most or least sensitive model parameters for estimation, it needs special attention, as the model involve many unknown parameters, and therefore it is beyond the scope of the present study. Tables 4 and 5 list the maximal reaction velocities (V_max_) and Michaelis-Menten constants (K_m_) for each reaction under the three muscle conditioned states.

**Table 5:** Phenomenological Michaelis-Menten kinetic constants for chemical species and energy controller ratios in 26 reactions for three different loading states (SED, LD, UL). These are estimated as discussed in the Method section

Glucose Utilization:				Glycogen Synthesis:			
GLU + ATP → G6P + ADP				G6P + ATP → GLY + ADP 2 + Pi			
	SED	LD	UL		SED	LD	UL
*K*_GLU_	0.07	0.07	0.14	*K*_G6P_	0.253	0.843	0.253
*K*_G6P_	0.253	0.084	0.253	*K*_GLY_	95	367	110
KATPADP MathType@MTEF@5@5@+=feaafiart1ev1aaatCvAUfKttLearuWrP9MDH5MBPbIqV92AaeXatLxBI9gBaebbnrfifHhDYfgasaacH8akY=wiFfYdH8Gipec8Eeeu0xXdbba9frFj0=OqFfea0dXdd9vqai=hGuQ8kuc9pgc9s8qqaq=dirpe0xb9q8qiLsFr0=vr0=vr0dc8meaabaqaciaacaGaaeqabaqabeGadaaakeaacqWGlbWsdaWgaaWcbaWaaSaaaeaacqqGbbqqcqqGubavcqqGqbauaeaacqqGbbqqcqqGebarcqqGqbauaaaabeaaaaa@34A5@	315	315	315	*K*_Pi_	2.7	9	2.7
				KATPADP MathType@MTEF@5@5@+=feaafiart1ev1aaatCvAUfKttLearuWrP9MDH5MBPbIqV92AaeXatLxBI9gBaebbnrfifHhDYfgasaacH8akY=wiFfYdH8Gipec8Eeeu0xXdbba9frFj0=OqFfea0dXdd9vqai=hGuQ8kuc9pgc9s8qqaq=dirpe0xb9q8qiLsFr0=vr0=vr0dc8meaabaqaciaacaGaaeqabaqabeGadaaakeaacqWGlbWsdaWgaaWcbaWaaSaaaeaacqqGbbqqcqqGubavcqqGqbauaeaacqqGbbqqcqqGebarcqqGqbauaaaabeaaaaa@34A5@	315	315	315
							
Glycogen Utilization:				Glucose 6-Phosphate Breakdown:			
GLY + Pi → G6P				G6P + ATP → 2 GA3P + ADP			
	SED	LD	UL		SED	LD	UL
*K*_GLY_	95	110	132	*K*_G6P_	0.253	0.169	0.38
*K*_Pi_	2.7	2.7	3.24	*K*_GA3P_	0.08	0.053	0.12
*K*_G6P_	0.253	0.253	0.304	KAMPATP MathType@MTEF@5@5@+=feaafiart1ev1aaatCvAUfKttLearuWrP9MDH5MBPbIqV92AaeXatLxBI9gBaebbnrfifHhDYfgasaacH8akY=wiFfYdH8Gipec8Eeeu0xXdbba9frFj0=OqFfea0dXdd9vqai=hGuQ8kuc9pgc9s8qqaq=dirpe0xb9q8qiLsFr0=vr0=vr0dc8meaabaqaciaacaGaaeqabaqabeGadaaakeaacqWGlbWsdaWgaaWcbaWaaSaaaeaacqqGbbqqcqqGnbqtcqqGqbauaeaacqqGbbqqcqqGubavcqqGqbauaaaabeaaaaa@34B7@	1.9e – 5	1.9e – 5	1.9e – 5
KAMPATP MathType@MTEF@5@5@+=feaafiart1ev1aaatCvAUfKttLearuWrP9MDH5MBPbIqV92AaeXatLxBI9gBaebbnrfifHhDYfgasaacH8akY=wiFfYdH8Gipec8Eeeu0xXdbba9frFj0=OqFfea0dXdd9vqai=hGuQ8kuc9pgc9s8qqaq=dirpe0xb9q8qiLsFr0=vr0=vr0dc8meaabaqaciaacaGaaeqabaqabeGadaaakeaacqWGlbWsdaWgaaWcbaWaaSaaaeaacqqGbbqqcqqGnbqtcqqGqbauaeaacqqGbbqqcqqGubavcqqGqbauaaaabeaaaaa@34B7@	3.2e – 6	3.2e – 6	3.2e – 6				
							
Glyceraldehyde 3-Phosphate Breakdown:				Pyruvate Production:			
GA3P + NAD^+ ^+ Pi → BPG + NADH				BPG + 2 ADP → PYR + 2 ATP			
	SED	LD	UL		SED	LD	UL
*K*_GA3P_	0.8	0.8	0.8	*K*_BPG_	0.4	0.4	0.4
*K*_Pi_	27	27	27	*K*_PYR_	0.238	0.238	0.238
*K*_BPG_	0.8	0.8	0.8	KADPATP MathType@MTEF@5@5@+=feaafiart1ev1aaatCvAUfKttLearuWrP9MDH5MBPbIqV92AaeXatLxBI9gBaebbnrfifHhDYfgasaacH8akY=wiFfYdH8Gipec8Eeeu0xXdbba9frFj0=OqFfea0dXdd9vqai=hGuQ8kuc9pgc9s8qqaq=dirpe0xb9q8qiLsFr0=vr0=vr0dc8meaabaqaciaacaGaaeqabaqabeGadaaakeaacqWGlbWsdaWgaaWcbaWaaSaaaeaacqqGbbqqcqqGebarcqqGqbauaeaacqqGbbqqcqqGubavcqqGqbauaaaabeaaaaa@34A5@	0.029	0.029	0.029
KNAD+NADH MathType@MTEF@5@5@+=feaafiart1ev1aaatCvAUfKttLearuWrP9MDH5MBPbIqV92AaeXatLxBI9gBaebbnrfifHhDYfgasaacH8akY=wiFfYdH8Gipec8Eeeu0xXdbba9frFj0=OqFfea0dXdd9vqai=hGuQ8kuc9pgc9s8qqaq=dirpe0xb9q8qiLsFr0=vr0=vr0dc8meaabaqaciaacaGaaeqabaqabeGadaaakeaacqWGlbWsdaWgaaWcbaWaaSaaaeaacqqGobGtcqqGbbqqcqqGebarcqqGRaWkaeaacqqGobGtcqqGbbqqcqqGebarcqqGibasaaaabeaaaaa@3671@	0.09	0.09	0.09				
							
Pyruvate Reduction:				Lactate Oxidation:			
PYR + NADH → LAC + NAD^+^				LAC + NAD^+ ^→ PYR + NADH			
	SED	LD	UL		SED	LD	UL
*K*_PYR_	0.0475	0.016	0.0475	*K*_LAC_	1.75	0.583	1.75
*K*_LAC_	1.75	0.583	1.75	*K*_PYR_	0.0475	0.016	0.0475
KNADHNAD+ MathType@MTEF@5@5@+=feaafiart1ev1aaatCvAUfKttLearuWrP9MDH5MBPbIqV92AaeXatLxBI9gBaebbnrfifHhDYfgasaacH8akY=wiFfYdH8Gipec8Eeeu0xXdbba9frFj0=OqFfea0dXdd9vqai=hGuQ8kuc9pgc9s8qqaq=dirpe0xb9q8qiLsFr0=vr0=vr0dc8meaabaqaciaacaGaaeqabaqabeGadaaakeaacqWGlbWsdaWgaaWcbaWaaSaaaeaacqqGobGtcqqGbbqqcqqGebarcqqGibasaeaacqqGobGtcqqGbbqqcqqGebarcqqGRaWkaaaabeaaaaa@3671@	1	1	1	KNAD+NADH MathType@MTEF@5@5@+=feaafiart1ev1aaatCvAUfKttLearuWrP9MDH5MBPbIqV92AaeXatLxBI9gBaebbnrfifHhDYfgasaacH8akY=wiFfYdH8Gipec8Eeeu0xXdbba9frFj0=OqFfea0dXdd9vqai=hGuQ8kuc9pgc9s8qqaq=dirpe0xb9q8qiLsFr0=vr0=vr0dc8meaabaqaciaacaGaaeqabaqabeGadaaakeaacqWGlbWsdaWgaaWcbaWaaSaaaeaacqqGobGtcqqGbbqqcqqGebarcqqGRaWkaeaacqqGobGtcqqGbbqqcqqGebarcqqGibasaaaabeaaaaa@3671@	9	9	9
							
Alanine Production:				Lipolysis:			
PYR → ALA				TGL → GLC + 3 FFA			
	SED	LD	UL		SED	LD	UL
*K*_PYR_	0.0475	0.0475	0.095	*K*_TGL_	14.8	52.2	18.5
*K*_ALA_	1.3	1.3	2.6	*K*_GLC_	0.062	0.186	0.062
				*K*_FFA_	0.57	1.71	0.57
							
Pyruvate Oxidation:				Triglyceride Synthesis:			
PYR + CoA + NAD^+ ^→ ACoA + NADH + CO_2_				GLC + 3 FFA + 7 ATP → TGL 7 + ADP + 7 Pi			
	SED	LD	UL		SED	LD	UL
*K*_PYR_	0.0475	0.0475	0.0475	*K*_GLC_	0.062	0.083	0.062
*K*_CoA_	0.0255	0.0255	0.0255	*K*_FFA_	0.57	0.76	0.57
*K*_ACoA_	2.2e – 3	2.2e – 3	2.2e – 3	*K*_TGL_	14.8	23.2	18.5
*K*_CO2_	23.6	23.6	23.6	*K*_Pi_	2.7	3.6	2.7
KNAD+NADH MathType@MTEF@5@5@+=feaafiart1ev1aaatCvAUfKttLearuWrP9MDH5MBPbIqV92AaeXatLxBI9gBaebbnrfifHhDYfgasaacH8akY=wiFfYdH8Gipec8Eeeu0xXdbba9frFj0=OqFfea0dXdd9vqai=hGuQ8kuc9pgc9s8qqaq=dirpe0xb9q8qiLsFr0=vr0=vr0dc8meaabaqaciaacaGaaeqabaqabeGadaaakeaacqWGlbWsdaWgaaWcbaWaaSaaaeaacqqGobGtcqqGbbqqcqqGebarcqqGRaWkaeaacqqGobGtcqqGbbqqcqqGebarcqqGibasaaaabeaaaaa@3671@	81	81	81	KATPADP MathType@MTEF@5@5@+=feaafiart1ev1aaatCvAUfKttLearuWrP9MDH5MBPbIqV92AaeXatLxBI9gBaebbnrfifHhDYfgasaacH8akY=wiFfYdH8Gipec8Eeeu0xXdbba9frFj0=OqFfea0dXdd9vqai=hGuQ8kuc9pgc9s8qqaq=dirpe0xb9q8qiLsFr0=vr0=vr0dc8meaabaqaciaacaGaaeqabaqabeGadaaakeaacqWGlbWsdaWgaaWcbaWaaSaaaeaacqqGbbqqcqqGubavcqqGqbauaeaacqqGbbqqcqqGebarcqqGqbauaaaabeaaaaa@34A5@	315	315	315
							
Free Fatty Acid Utilization:				Fatty Acyl-CoA Oxidation:			
FFA + CoA + 2 ATP → FAC + 2ADP + 2Pi				FAC + 7 CoA + (35/3) NAD^+ ^→ 8 ACoA + (35/3) NADH			
	SED	LD	UL		SED	LD	UL
*K*_FFA_	0.57	0.76	0.43	*K*_FAC_	3.5e – 3	4.35e – 3	2.78e – 3
*K*_CoA_	0.026	0.034	0.019	*K*_CoA_	0.0255	0.0319	0.02
*K*_FAC_	3.5e – 3	4.6e – 3	2.6e – 3	*K*_ACoA_	2.2e – 3	2.8e – 3	1.8e – 3
*K*_Pi_	2.7	3.6	2	KNAD+NADH MathType@MTEF@5@5@+=feaafiart1ev1aaatCvAUfKttLearuWrP9MDH5MBPbIqV92AaeXatLxBI9gBaebbnrfifHhDYfgasaacH8akY=wiFfYdH8Gipec8Eeeu0xXdbba9frFj0=OqFfea0dXdd9vqai=hGuQ8kuc9pgc9s8qqaq=dirpe0xb9q8qiLsFr0=vr0=vr0dc8meaabaqaciaacaGaaeqabaqabeGadaaakeaacqWGlbWsdaWgaaWcbaWaaSaaaeaacqqGobGtcqqGbbqqcqqGebarcqqGRaWkaeaacqqGobGtcqqGbbqqcqqGebarcqqGibasaaaabeaaaaa@3671@	9	9	9
KATPADP MathType@MTEF@5@5@+=feaafiart1ev1aaatCvAUfKttLearuWrP9MDH5MBPbIqV92AaeXatLxBI9gBaebbnrfifHhDYfgasaacH8akY=wiFfYdH8Gipec8Eeeu0xXdbba9frFj0=OqFfea0dXdd9vqai=hGuQ8kuc9pgc9s8qqaq=dirpe0xb9q8qiLsFr0=vr0=vr0dc8meaabaqaciaacaGaaeqabaqabeGadaaakeaacqWGlbWsdaWgaaWcbaWaaSaaaeaacqqGbbqqcqqGubavcqqGqbauaeaacqqGbbqqcqqGebarcqqGqbauaaaabeaaaaa@34A5@	315	315	315				
							
Citrate Production:				α-Ketoglutarate Production:			
ACoA + OXA → CIT + CoA				CIT + NAD^+ ^→ AKG + NADH + CO_2_			
	SED	LD	UL		SED	LD	UL
*K*_ACoA_	2.2e – 3	3.2e – 3	1.8e – 3	*K*_CIT_	0.103	0.412	0.0824
*K*_OXA_	3.0e – 3	4.3e – 3	2.4e – 3	*K*_AKG_	0.0125	0.05	0.0125
*K*_CIT_	0.103	0.147	0.082	*K*_CO2_	23.6	94.4	23.6
*K*_CoA_	0.0255	0.0364	0.02	KNAD+NADH MathType@MTEF@5@5@+=feaafiart1ev1aaatCvAUfKttLearuWrP9MDH5MBPbIqV92AaeXatLxBI9gBaebbnrfifHhDYfgasaacH8akY=wiFfYdH8Gipec8Eeeu0xXdbba9frFj0=OqFfea0dXdd9vqai=hGuQ8kuc9pgc9s8qqaq=dirpe0xb9q8qiLsFr0=vr0=vr0dc8meaabaqaciaacaGaaeqabaqabeGadaaakeaacqWGlbWsdaWgaaWcbaWaaSaaaeaacqqGobGtcqqGbbqqcqqGebarcqqGRaWkaeaacqqGobGtcqqGbbqqcqqGebarcqqGibasaaaabeaaaaa@3671@	9	9	9
							
Succinyl-CoA Production:				Succinate Formation:			
AKG + CoA + NAD^+ ^→ SCoA + NADH + CO_2_				SCoA +ADP + Pi → SUC + CoA + ATP			
	SED	LD	UL		SED	LD	UL
*K*_AKG_	0.0125	0.0179	0.01	*K*_SCoA_	0.123	0.176	0.0984
*K*_CoA_	0.0255	0.0364	0.02	*K*_Pi_	2.7	3.9	2.2
*K*_SCoA_	0.123	0.176	0.0984	*K*_SUC_	0.095	0.136	0.076
*K*_CO2_	23.6	33.7	18.9	*K*_CoA_	0.0255	0.0364	0.02
KNAD+NADH MathType@MTEF@5@5@+=feaafiart1ev1aaatCvAUfKttLearuWrP9MDH5MBPbIqV92AaeXatLxBI9gBaebbnrfifHhDYfgasaacH8akY=wiFfYdH8Gipec8Eeeu0xXdbba9frFj0=OqFfea0dXdd9vqai=hGuQ8kuc9pgc9s8qqaq=dirpe0xb9q8qiLsFr0=vr0=vr0dc8meaabaqaciaacaGaaeqabaqabeGadaaakeaacqWGlbWsdaWgaaWcbaWaaSaaaeaacqqGobGtcqqGbbqqcqqGebarcqqGRaWkaeaacqqGobGtcqqGbbqqcqqGebarcqqGibasaaaabeaaaaa@3671@	9	9	9	KADPATP MathType@MTEF@5@5@+=feaafiart1ev1aaatCvAUfKttLearuWrP9MDH5MBPbIqV92AaeXatLxBI9gBaebbnrfifHhDYfgasaacH8akY=wiFfYdH8Gipec8Eeeu0xXdbba9frFj0=OqFfea0dXdd9vqai=hGuQ8kuc9pgc9s8qqaq=dirpe0xb9q8qiLsFr0=vr0=vr0dc8meaabaqaciaacaGaaeqabaqabeGadaaakeaacqWGlbWsdaWgaaWcbaWaaSaaaeaacqqGbbqqcqqGebarcqqGqbauaeaacqqGbbqqcqqGubavcqqGqbauaaaabeaaaaa@34A5@	3.2e – 3	3.2e – 3	3.2e – 3
							
Malate Production:				Oxaloacetate Production:			
SUC + (2/3) NAD^+ ^→ MAL + (2/3) NADH				MAL + NAD^+ ^→ OXA + NADH			
	SED	LD	UL		SED	LD	UL
*K*_SUC_	0.095	0.238	0.0475	*K*_MAL_	0.0975	0.244	0.0488
*K*_MAL_	0.0975	0.244	0.0488	*K*_OXA_	3.0e – 3	7.5e – 3	1.5e – 3
KNAD+NADH MathType@MTEF@5@5@+=feaafiart1ev1aaatCvAUfKttLearuWrP9MDH5MBPbIqV92AaeXatLxBI9gBaebbnrfifHhDYfgasaacH8akY=wiFfYdH8Gipec8Eeeu0xXdbba9frFj0=OqFfea0dXdd9vqai=hGuQ8kuc9pgc9s8qqaq=dirpe0xb9q8qiLsFr0=vr0=vr0dc8meaabaqaciaacaGaaeqabaqabeGadaaakeaacqWGlbWsdaWgaaWcbaWaaSaaaeaacqqGobGtcqqGbbqqcqqGebarcqqGRaWkaeaacqqGobGtcqqGbbqqcqqGebarcqqGibasaaaabeaaaaa@3671@	9	9	9	KNAD+NADH MathType@MTEF@5@5@+=feaafiart1ev1aaatCvAUfKttLearuWrP9MDH5MBPbIqV92AaeXatLxBI9gBaebbnrfifHhDYfgasaacH8akY=wiFfYdH8Gipec8Eeeu0xXdbba9frFj0=OqFfea0dXdd9vqai=hGuQ8kuc9pgc9s8qqaq=dirpe0xb9q8qiLsFr0=vr0=vr0dc8meaabaqaciaacaGaaeqabaqabeGadaaakeaacqWGlbWsdaWgaaWcbaWaaSaaaeaacqqGobGtcqqGbbqqcqqGebarcqqGRaWkaeaacqqGobGtcqqGbbqqcqqGebarcqqGibasaaaabeaaaaa@3671@	9	9	9
							
Phosphocreatine Breakdown:				Phosphocreatine Synthesis:			
PCR + ADP → CR + ATP				CR + ATP → PCR + ADP			
	SED	LD	UL		SED	LD	UL
*K*_PCR_	20	20	20	*K*_CR_	10	10	10
*K*_CR_	10	10	10	*K*_PCR_	20	20	20
KADPATP MathType@MTEF@5@5@+=feaafiart1ev1aaatCvAUfKttLearuWrP9MDH5MBPbIqV92AaeXatLxBI9gBaebbnrfifHhDYfgasaacH8akY=wiFfYdH8Gipec8Eeeu0xXdbba9frFj0=OqFfea0dXdd9vqai=hGuQ8kuc9pgc9s8qqaq=dirpe0xb9q8qiLsFr0=vr0=vr0dc8meaabaqaciaacaGaaeqabaqabeGadaaakeaacqWGlbWsdaWgaaWcbaWaaSaaaeaacqqGbbqqcqqGebarcqqGqbauaeaacqqGbbqqcqqGubavcqqGqbauaaaabeaaaaa@34A5@	3.2e – 3	3.2e – 3	3.2e – 3	KATPADP MathType@MTEF@5@5@+=feaafiart1ev1aaatCvAUfKttLearuWrP9MDH5MBPbIqV92AaeXatLxBI9gBaebbnrfifHhDYfgasaacH8akY=wiFfYdH8Gipec8Eeeu0xXdbba9frFj0=OqFfea0dXdd9vqai=hGuQ8kuc9pgc9s8qqaq=dirpe0xb9q8qiLsFr0=vr0=vr0dc8meaabaqaciaacaGaaeqabaqabeGadaaakeaacqWGlbWsdaWgaaWcbaWaaSaaaeaacqqGbbqqcqqGubavcqqGqbauaeaacqqGbbqqcqqGebarcqqGqbauaaaabeaaaaa@34A5@	315	315	315
							
Oxygen Utilization:				ATP Hydrolysis			
O_2 _+ 5.63 ADP + 5.63 Pi + 1.88 NADH → 2 H_2_O + 5.63 ATP + 1.88 NAD^+^				ATP → ADP + Pi			
	SED	LD	UL		SED	LD	UL
*K*_O2_	0.01	0.01	0.01	*K*_ATP_	0.063	0.063	0.063
*K*_NADH_	0.07	0.09	0.06				
*K*_Pi_	3.8	4.9	3.2				
KNAD+ MathType@MTEF@5@5@+=feaafiart1ev1aaatCvAUfKttLearuWrP9MDH5MBPbIqV92AaeXatLxBI9gBaebbnrfifHhDYfgasaacH8akY=wiFfYdH8Gipec8Eeeu0xXdbba9frFj0=OqFfea0dXdd9vqai=hGuQ8kuc9pgc9s8qqaq=dirpe0xb9q8qiLsFr0=vr0=vr0dc8meaabaqaciaacaGaaeqabaqabeGadaaakeaacqWGlbWsdaWgaaWcbaGaeeOta4KaeeyqaeKaeeiraq0aaWbaaWqabeaacqGHRaWkaaaaleqaaaaa@324D@	0.63	0.81	0.54				
KADPATP MathType@MTEF@5@5@+=feaafiart1ev1aaatCvAUfKttLearuWrP9MDH5MBPbIqV92AaeXatLxBI9gBaebbnrfifHhDYfgasaacH8akY=wiFfYdH8Gipec8Eeeu0xXdbba9frFj0=OqFfea0dXdd9vqai=hGuQ8kuc9pgc9s8qqaq=dirpe0xb9q8qiLsFr0=vr0=vr0dc8meaabaqaciaacaGaaeqabaqabeGadaaakeaacqWGlbWsdaWgaaWcbaWaaSaaaeaacqqGbbqqcqqGebarcqqGqbauaeaacqqGbbqqcqqGubavcqqGqbauaaaabeaaaaa@34A5@	6.3e – 3	6.3e – 3	6.3e – 3				
							
AMP Utilization:				AMP Production:			
AMP + ATP → 2 ADP				2 ADP → AMP + ATP			
	SED	LD	UL		SED	LD	UL
*K*_AMP_	6e – 5	6e – 5	6e – 5	*K*_ADP_	0.02	0.02	0.02
*K*_ATP_	6.3	6.3	6.3	*K*_AMP_	6e – 5	6e – 5	6e – 5
*K*_ADP_	0.02	0.02	0.02	*K*_ATP_	6.3	6.3	6.3

The conversion factor relating work rate to ATP turnover rate is derived from known relationships between muscle VO_2 _and work rate [29]. The relationship between muscle volume recruitment and work rate was determined from MRI studies quantifying the amount of active muscle for different work rates [41,42]. Parameters characterizing the dynamics of blood flow response to exercise were estimated from known relationships between muscle blood flow and work rate [29,70] and studies using Doppler ultrasound to measure the femoral artery blood velocity [53]. The steady-state blood flow for a particular work rate is found using the known relationship between muscle blood flow and work rate [29,70]. Finally, the relative metabolic rate term that links processes of ATP synthesis to ATP hydrolysis is a function of mechanical efficiency and resting metabolic rate. The mechanical efficiency, which converts external work into units of metabolic watts, is determined from studies that measure the mechanical work output as a result of free energy change through ATP hydrolysis [54,71].

To differentiate between different loading states, parameter values characterizing these states were used in simulations, namely the maximal reaction velocities (V_max_) of the enzymes and time constant characterizing the blood flow response. These parameters are directly linked to adaptations occurring as a result of either chronic loading or unloading. Indeed, in the model representing loading, significant modifications were made to the V_max _values of the oxidative enzymes according to relative percent changes measured experimentally. This change reflects the considerable increases in both mitochondrial size and density within the tissue [10]. Also, the time constant for blood flow (τ_Q_) was reduced in the loading model to account for the increase in muscle capillarization [53]; τ_Q _in the unloading model was accordingly increased to reflect the presence of fewer capillaries perfusing muscle fibers [8,63]. In regards to the unloading model, experimental data shows that more emphasis is placed on carbohydrate metabolism for ATP production, in particular glycogenolysis. Therefore, the V_max _values for glycogen phosphorylase and glucose phosphorylation were increased with accompanying decreases in oxidative enzymes' maximal reaction velocities [22,23].

## Results

We first compared computer-simulated responses with experimental data obtained in healthy individuals during a 10-min moderate-intensity exercise bout (30–45% VO_2 max_, WR = 65W) on a cycle ergometer. Then, the steady-state and dynamic responses of individuals who have under-gone chronic interventions involving marked enhancements (loading) or reductions (unloading) in physical activity to a step change in ATP turnover rate, equivalent to the same change in work rate (WR = 65W), were compared to those obtained in healthy sedentary subjects. From these results, we determined quantitatively the effects of loading and unloading adaptations on fuel preference during submaximal exercise.

### Dynamic responses to submaximal exercise in sedentary state

A moderate-intensity exercise bout (30–45% VO_2 max_) for a normal, sedentary individual was simulated for 10 min by inducing changes in ATP turnover rate and other physiological variables equivalent to a step increase in work rate WR from 0 to 65 W. Active muscle mass increased instantaneously from 4 to 7.5 kg ww (Eq. 7), while muscle blood flow increased exponentially (τ_Q _≈ 0.35 min) from 0.9 to 6.0 L/min (Eq. 8). The rates of ATP production and utilization were closely matched, as the ATP concentration was stable throughout the exercise bout. In contrast, exercise caused a 40% drop in NADH concentration, from 0.05 to 0.03 mM [60,69]; this decline occurred in an exponential fashion, with the new steady-state in [NADH] reaching within 1 min of exercise onset, as depicted in Figure 3.

Model-simulated muscle oxygen uptake (VO_2 _= Q(C_a,O2 _– σ_O2_C_c,O2_)) increased exponentially (τ_VO2 _≈ 0.5 min) from 2.75 mmol/min at rest to 36.5 mmol/min during exercise while muscle CO_2 _release (VCO_2 _= Q(C_a,CO2 _- σ_CO2_C_c,CO2_)) increased slightly slower (τ_VCO2 _≈ 0.6 min) from 2.16 to 30.4 mmol/min, as depicted in Figure 4. The respiratory quotient (RQ = VCO_2_/VO_2_) increased from 0.785 at rest to 0.832 during 10-min moderate-intensity exercise bout. The [PCr] dynamic response closely mirrored that of VO_2 _[72-74] decreasing about 10% from its resting value of 20 mM to about 16 mM during exercise with a τ_PCr _≈ 0.5 min, as depicted in Figure 5. Therefore, the model supports one of the key hypotheses in muscle bioenergetics that the muscle [PCr] and VO_2 _dynamics match very well during moderate-intensity exercise [72-74]. The model also predicts linear relationships between the steady-state [PCr] and WR and steady-state VO_2 _and WR during submaximal exercise bouts (0 ≤ WR ≤ 100 Watts), indicating that the model successfully mimic the linear relationship between [PCR] and VO_2 _at steady-state (not shown), seen experimentally [75]. The concentration of Pi (inorganic phosphate) increased from 2.7 mmol/kg ww at rest to about 4.6 mmol/kg ww during exercise with a τ_Pi _equivalent to τ_PCr _(Fig 5).

Muscle glucose uptake increased from 0.18 to 1.76 mmol/min within the first 30 seconds of exercise onset, and then decreased to its steady-state value of 1.38 mmol/min, as shown in Figure 6. The net glycogen breakdown increased rapidly from 0.0 to 0.15 mmol/min, resulting in a linear decrease in glycogen concentration from 95 to 93.5 mM during 10-min moderate-intensity exercise bout. Concentrations of both glucose 6-phosphate and pyruvate increased ~ 35% from their resting values in an exponential manner (τ ≈ 0.85 min). The lactate release from muscle increased abruptly from 0.11 to 0.63 mmol/min before reaching its steady-state value of 0.32 mmol/min, as shown in Figure 6. Both glucose uptake and lactate release were seen to have a biphasic behavior.

**Figure 7:**
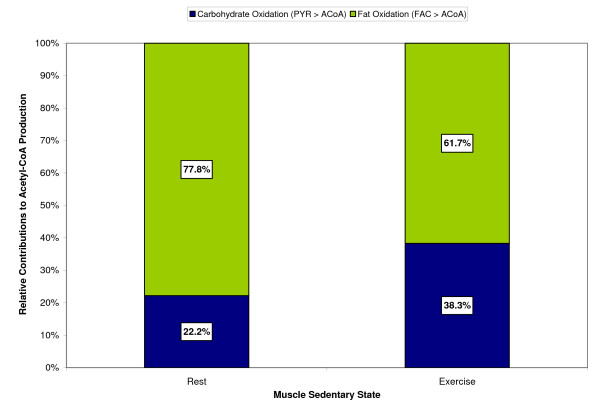
Simulated relative contributions from carbohydrates and fats towards the steady-state acetyl-CoA production at rest and during a 10-min moderate-intensity exercise bout (WR = 65W) of a sedentary individual.

During 10-min exercise bout, the concentration of acetyl-CoA increased 50% from its resting value of 2.23 μM, with the carbohydrates contributing a larger portion toward the increased acetyl-CoA production, as shown in Figure 7. At rest, approximately 22% of the acetyl-CoA produced came from pyruvate oxidation, with the remaining 78% coming from fat oxidation. However, during exercise, the pyruvate oxidation contributed about 38% towards total acetyl-CoA production, with the fats contributing about 62%. This causes an increase in the muscle respiratory quotient (RQ) from 0.785 at rest to 0.832 at the end of 10-min exercise, though not significantly, but within the experimental range for moderate-intensity exercise [67,68].

### Dynamic responses to submaximal exercise after chronic loading or unloading

**Figure 8:**
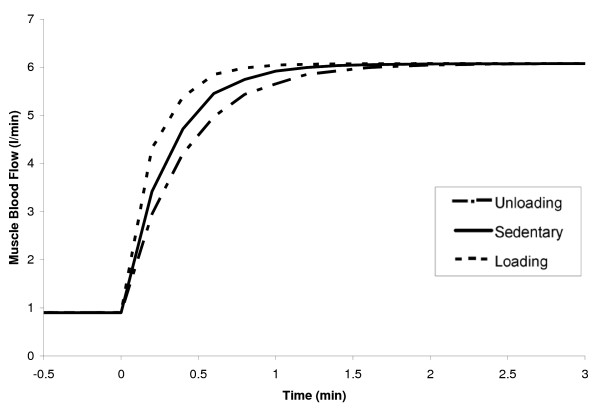
Skeletal muscle blood flow responses to a moderate-intensity exercise bout (WR = 65W) for three different muscle states. The range of the x-axis is reduced in order to amplify the transient changes between the chronically-loaded and unloaded states. The time constant of blood flow (τ_Q_) for loaded, sedentary and unloaded muscles was set as 0.25, 0.35 and 0.45 min, respectively (Table 1).

**Figure 9:**
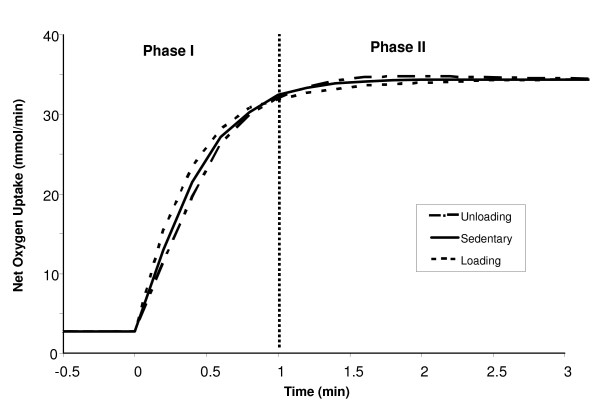
Simulated dynamic oxygen uptake responses during a 10-min moderate-intensity exercise bout (WR = 65W) for sedentary and chronically-loaded/unloaded individuals. The range of the x-axis is reduced in order to amplify the transient changes between loading states. The vertical bar located at time = 1 min divides the response into two phases. The initial muscle VO_2 _responses at the onset of exercise (phase I) are largely functions of the blood flow response. In phase II, the responses are dependent on the intramuscular oxygen stores. The time constant of muscle VO_2 _(τ_VO2_) for loaded, sedentary and unloaded muscles was about 0.4, 0.5 and 0.56 min, respectively.

The dynamic responses of selected metabolite concentrations and reaction fluxes to 10 min of moderate-intensity, constant work rate exercise (WR = 65W) were compared for the chronic loading (LD) and unloading (UL) states. The simulations previously described for the sedentary (SED) state served as the control state. The pattern of muscle mass recruited at the onset of exercise was assumed the same in all three groups (Eq. 7, Table 1). Muscle blood flow for LD and UL states was increased in an exponential manner (input) towards the same steady-state value, with LD reaching it faster than UL (τQLD
 MathType@MTEF@5@5@+=feaafiart1ev1aaatCvAUfKttLearuWrP9MDH5MBPbIqV92AaeXatLxBI9gBaebbnrfifHhDYfgasaacH8akY=wiFfYdH8Gipec8Eeeu0xXdbba9frFj0=OqFfea0dXdd9vqai=hGuQ8kuc9pgc9s8qqaq=dirpe0xb9q8qiLsFr0=vr0=vr0dc8meaabaqaciaacaGaaeqabaqabeGadaaakeaaiiGacqWFepaDdaqhaaWcbaGaemyuaefabaGaeeitaWKaeeiraqeaaaaa@31FE@ ≈ 0.25 min vs. τQUL
 MathType@MTEF@5@5@+=feaafiart1ev1aaatCvAUfKttLearuWrP9MDH5MBPbIqV92AaeXatLxBI9gBaebbnrfifHhDYfgasaacH8akY=wiFfYdH8Gipec8Eeeu0xXdbba9frFj0=OqFfea0dXdd9vqai=hGuQ8kuc9pgc9s8qqaq=dirpe0xb9q8qiLsFr0=vr0=vr0dc8meaabaqaciaacaGaaeqabaqabeGadaaakeaaiiGacqWFepaDdaqhaaWcbaGaemyuaefabaGaeeyvauLaeeitaWeaaaaa@3220@ ≈ 0.45 min; Eq. 8, Table 1), as shown in Figure 8. Similar transient profiles were predicted from model simulations for muscle oxygen uptake (VO_2_), with differences between LD and UL states occurring only in the transient phase, as shown in Figure 9. In the first minute, the muscle VO_2 _for a LD individual reached steady state sooner than a UL individual (τVO2LD
 MathType@MTEF@5@5@+=feaafiart1ev1aaatCvAUfKttLearuWrP9MDH5MBPbIqV92AaeXatLxBI9gBaebbnrfifHhDYfgasaacH8akY=wiFfYdH8Gipec8Eeeu0xXdbba9frFj0=OqFfea0dXdd9vqai=hGuQ8kuc9pgc9s8qqaq=dirpe0xb9q8qiLsFr0=vr0=vr0dc8meaabaqaciaacaGaaeqabaqabeGadaaakeaaiiGacqWFepaDdaqhaaWcbaGaeeOvayLaee4ta8KaeeOmaidabaGaeeitaWKaeeiraqeaaaaa@3416@ ≈ 0.4 min vs. τVO2UL
 MathType@MTEF@5@5@+=feaafiart1ev1aaatCvAUfKttLearuWrP9MDH5MBPbIqV92AaeXatLxBI9gBaebbnrfifHhDYfgasaacH8akY=wiFfYdH8Gipec8Eeeu0xXdbba9frFj0=OqFfea0dXdd9vqai=hGuQ8kuc9pgc9s8qqaq=dirpe0xb9q8qiLsFr0=vr0=vr0dc8meaabaqaciaacaGaaeqabaqabeGadaaakeaaiiGacqWFepaDdaqhaaWcbaGaeeOvayLaee4ta8KaeeOmaidabaGaeeyvauLaeeitaWeaaaaa@3438@ ≈ 0.55 min). As a result of this faster delivery of arterial O_2 _to the tissue in LD muscles, the LD intramuscular O_2 _stores are spared, and the increase in VO_2_(LD) is more gradual after the first minute of exercise onset until a new steady-state is reached. In UL muscles, with a larger reliance on tissue O_2 _during the initial minute of exercise, VO_2_(UL) increases more rapidly from minute one until the new steady-state is reached. However, in both states, the muscle VO_2 _dynamics was slower than the muscle blood flow dynamics, which indicates that O_2 _delivery to the tissue was limited due to the availability of stored O_2 _in the tissue.

**Figure 10:**
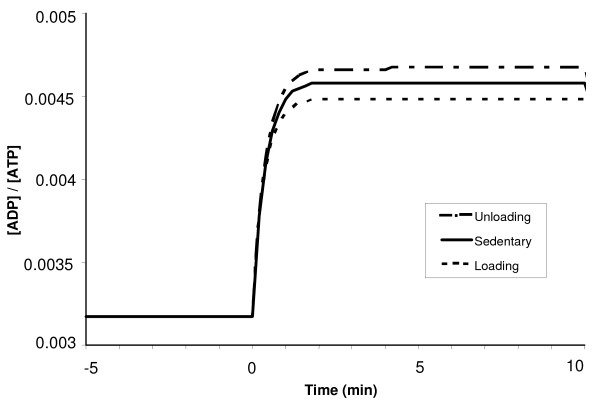
Simulated dynamic responses of the phosphorylation ratio [ADP]/[ATP] during a 10-min moderate-intensity exercise period (WR = 65W) for sedentary and chronically-loaded/unloaded individuals. The steady state responses are present from time -5 min to time 0 min when the step change in work rate (WR) is initiated.

**Figure 11:**
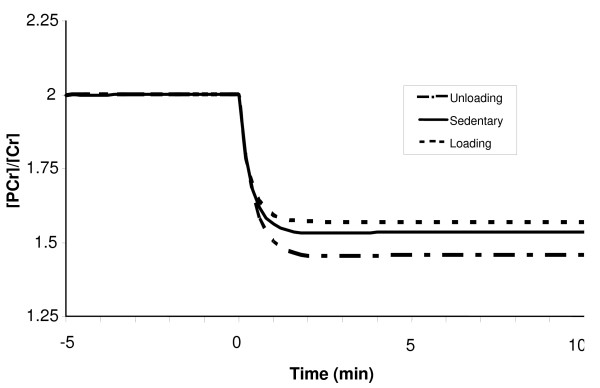
Simulated dynamic responses of the creatine ratio [PCr]/[Cr] during a 10-min moderate-intensity exercise period (WR = 65W) for sedentary and chronically-loaded/unloaded individuals. The dynamics of [PCr]/[Cr] ratio matches well with the dynamics of [ADP]/[ATP] ratio, with τADP/ATPLD
 MathType@MTEF@5@5@+=feaafiart1ev1aaatCvAUfKttLearuWrP9MDH5MBPbIqV92AaeXatLxBI9gBaebbnrfifHhDYfgasaacH8akY=wiFfYdH8Gipec8Eeeu0xXdbba9frFj0=OqFfea0dXdd9vqai=hGuQ8kuc9pgc9s8qqaq=dirpe0xb9q8qiLsFr0=vr0=vr0dc8meaabaqaciaacaGaaeqabaqabeGadaaakeaaiiGacqWFepaDdaqhaaWcbaGaeeyqaeKaeeiraqKaeeiuaaLaee4la8IaeeyqaeKaeeivaqLaeeiuaafabaGaeeitaWKaeeiraqeaaaaa@3856@ ≈ τPCr/CrLD
 MathType@MTEF@5@5@+=feaafiart1ev1aaatCvAUfKttLearuWrP9MDH5MBPbIqV92AaeXatLxBI9gBaebbnrfifHhDYfgasaacH8akY=wiFfYdH8Gipec8Eeeu0xXdbba9frFj0=OqFfea0dXdd9vqai=hGuQ8kuc9pgc9s8qqaq=dirpe0xb9q8qiLsFr0=vr0=vr0dc8meaabaqaciaacaGaaeqabaqabeGadaaakeaaiiGacqWFepaDdaqhaaWcbaGaeeiuaaLaee4qamKaeeOCaiNaee4la8Iaee4qamKaeeOCaihabaGaeeitaWKaeeiraqeaaaaa@37CF@ ≈ 0.3 min, τADP/ATPSED
 MathType@MTEF@5@5@+=feaafiart1ev1aaatCvAUfKttLearuWrP9MDH5MBPbIqV92AaeXatLxBI9gBaebbnrfifHhDYfgasaacH8akY=wiFfYdH8Gipec8Eeeu0xXdbba9frFj0=OqFfea0dXdd9vqai=hGuQ8kuc9pgc9s8qqaq=dirpe0xb9q8qiLsFr0=vr0=vr0dc8meaabaqaciaacaGaaeqabaqabeGadaaakeaaiiGacqWFepaDdaqhaaWcbaGaeeyqaeKaeeiraqKaeeiuaaLaee4la8IaeeyqaeKaeeivaqLaeeiuaafabaGaee4uamLaeeyrauKaeeiraqeaaaaa@3975@ ≈ τPCr/CrSED
 MathType@MTEF@5@5@+=feaafiart1ev1aaatCvAUfKttLearuWrP9MDH5MBPbIqV92AaeXatLxBI9gBaebbnrfifHhDYfgasaacH8akY=wiFfYdH8Gipec8Eeeu0xXdbba9frFj0=OqFfea0dXdd9vqai=hGuQ8kuc9pgc9s8qqaq=dirpe0xb9q8qiLsFr0=vr0=vr0dc8meaabaqaciaacaGaaeqabaqabeGadaaakeaaiiGacqWFepaDdaqhaaWcbaGaeeiuaaLaee4qamKaeeOCaiNaee4la8Iaee4qamKaeeOCaihabaGaee4uamLaeeyrauKaeeiraqeaaaaa@38EE@ ≈ 0.35 min, and τADP/ATPUL
 MathType@MTEF@5@5@+=feaafiart1ev1aaatCvAUfKttLearuWrP9MDH5MBPbIqV92AaeXatLxBI9gBaebbnrfifHhDYfgasaacH8akY=wiFfYdH8Gipec8Eeeu0xXdbba9frFj0=OqFfea0dXdd9vqai=hGuQ8kuc9pgc9s8qqaq=dirpe0xb9q8qiLsFr0=vr0=vr0dc8meaabaqaciaacaGaaeqabaqabeGadaaakeaaiiGacqWFepaDdaqhaaWcbaGaeeyqaeKaeeiraqKaeeiuaaLaee4la8IaeeyqaeKaeeivaqLaeeiuaafabaGaeeyvauLaeeitaWeaaaaa@3878@ ≈ τPCr/CrUL
 MathType@MTEF@5@5@+=feaafiart1ev1aaatCvAUfKttLearuWrP9MDH5MBPbIqV92AaeXatLxBI9gBaebbnrfifHhDYfgasaacH8akY=wiFfYdH8Gipec8Eeeu0xXdbba9frFj0=OqFfea0dXdd9vqai=hGuQ8kuc9pgc9s8qqaq=dirpe0xb9q8qiLsFr0=vr0=vr0dc8meaabaqaciaacaGaaeqabaqabeGadaaakeaaiiGacqWFepaDdaqhaaWcbaGaeeiuaaLaee4qamKaeeOCaiNaee4la8Iaee4qamKaeeOCaihabaGaeeyvauLaeeitaWeaaaaa@37F1@ ≈ 0.45 min.

At exercise onset, the phosphorylation ratio [ADP]/[ATP] increases exponentially, while the creatine ratio [PCr]/[Cr] decreases exponentially with the similar time constant (τADP/ATPLD
 MathType@MTEF@5@5@+=feaafiart1ev1aaatCvAUfKttLearuWrP9MDH5MBPbIqV92AaeXatLxBI9gBaebbnrfifHhDYfgasaacH8akY=wiFfYdH8Gipec8Eeeu0xXdbba9frFj0=OqFfea0dXdd9vqai=hGuQ8kuc9pgc9s8qqaq=dirpe0xb9q8qiLsFr0=vr0=vr0dc8meaabaqaciaacaGaaeqabaqabeGadaaakeaaiiGacqWFepaDdaqhaaWcbaGaeeyqaeKaeeiraqKaeeiuaaLaee4la8IaeeyqaeKaeeivaqLaeeiuaafabaGaeeitaWKaeeiraqeaaaaa@3856@ ≈ τPCr/CrLD
 MathType@MTEF@5@5@+=feaafiart1ev1aaatCvAUfKttLearuWrP9MDH5MBPbIqV92AaeXatLxBI9gBaebbnrfifHhDYfgasaacH8akY=wiFfYdH8Gipec8Eeeu0xXdbba9frFj0=OqFfea0dXdd9vqai=hGuQ8kuc9pgc9s8qqaq=dirpe0xb9q8qiLsFr0=vr0=vr0dc8meaabaqaciaacaGaaeqabaqabeGadaaakeaaiiGacqWFepaDdaqhaaWcbaGaeeiuaaLaee4qamKaeeOCaiNaee4la8Iaee4qamKaeeOCaihabaGaeeitaWKaeeiraqeaaaaa@37CF@ ≈ 0.3 min and τADP/ATPUL
 MathType@MTEF@5@5@+=feaafiart1ev1aaatCvAUfKttLearuWrP9MDH5MBPbIqV92AaeXatLxBI9gBaebbnrfifHhDYfgasaacH8akY=wiFfYdH8Gipec8Eeeu0xXdbba9frFj0=OqFfea0dXdd9vqai=hGuQ8kuc9pgc9s8qqaq=dirpe0xb9q8qiLsFr0=vr0=vr0dc8meaabaqaciaacaGaaeqabaqabeGadaaakeaaiiGacqWFepaDdaqhaaWcbaGaeeyqaeKaeeiraqKaeeiuaaLaee4la8IaeeyqaeKaeeivaqLaeeiuaafabaGaeeyvauLaeeitaWeaaaaa@3878@ ≈ τPCr/CrUL
 MathType@MTEF@5@5@+=feaafiart1ev1aaatCvAUfKttLearuWrP9MDH5MBPbIqV92AaeXatLxBI9gBaebbnrfifHhDYfgasaacH8akY=wiFfYdH8Gipec8Eeeu0xXdbba9frFj0=OqFfea0dXdd9vqai=hGuQ8kuc9pgc9s8qqaq=dirpe0xb9q8qiLsFr0=vr0=vr0dc8meaabaqaciaacaGaaeqabaqabeGadaaakeaaiiGacqWFepaDdaqhaaWcbaGaeeiuaaLaee4qamKaeeOCaiNaee4la8Iaee4qamKaeeOCaihabaGaeeyvauLaeeitaWeaaaaa@37F1@ ≈ 0.45 min), as shown in Figures 10 and 11. So the model supports the hypothesis that the dynamics of [ADP]/[ATP] and [PCr]/[Cr] ratios match very well during moderate-intensity exercise [74], even in LD and UL muscles. The steady-state [ADP]/[ATP] ratio during exercise for the UL muscle was about 7% higher than that for LD muscle (Fig 10), suggesting a larger uncoupling between ATP utilization and ATP formation in the UL muscle. A 7% greater decline in steady-state [PCr]/[Cr] ratio occurred during exercise in the UL muscles compared with the LD muscles as a result of increased reliance on PCr (Fig 11).

**Figure 12:**
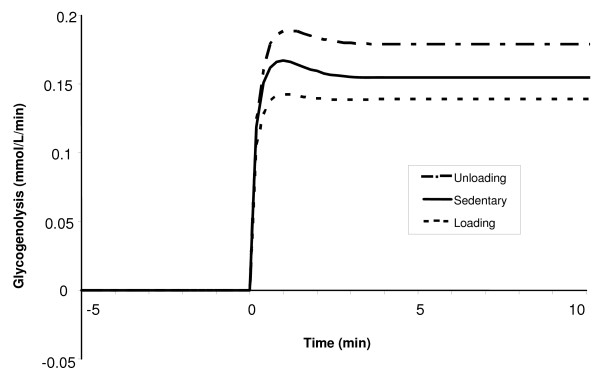
Simulated dynamic responses of the net glycogen breakdown in muscle tissue during a 10-min moderate-intensity exercise period (WR = 65W) for sedentary and chronically-loaded/unloaded individuals. The steady state responses are present from time -5 min to time 0 min when the step change in work rate (WR) is initiated.

Figure 12 shows the net glycogen breakdown rates for the three conditioned muscle states during a 10-min moderate-intensity exercise bout. In each case, the glycogenolysis initially increases rapidly before slowly decreasing towards a new steady-state level. It is seen that the net glycogen breakdown rate for UL muscle increases from 0 at rest to 0.18 mM/min over the 10-min exercise period, while in the LD muscle, the response shows an increase from 0 to 0.13 mM/min. Thus, the chronically loaded muscle utilizes less glycogen for a submaximal exercise bout.

**Figure 13:**
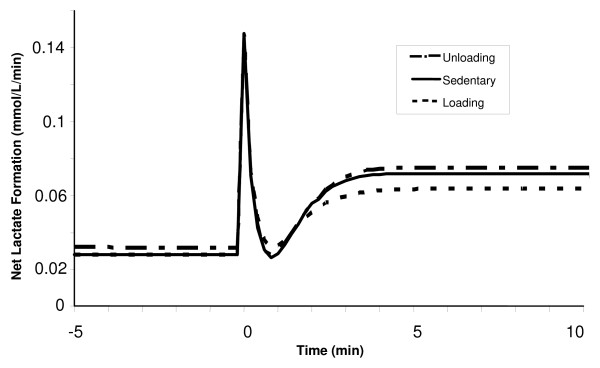
Simulated dynamic responses of the net lactate formation in muscle tissue during a 10-min moderate-intensity exercise period (WR = 65W) for sedentary and chronically-loaded/unloaded individuals. The net lactate formation has tri-phasic behavior.

The increased reliance on glycogen for the unloading state results in a 10% higher rate of glycolysis during moderate-intensity exercise compared with the loading state. This difference ultimately leads to higher concentrations of glycolytic intermediates such as glucose 6-phosphate and pyruvate for the unloading state. Since there were no differences in the redox ratio between conditioned states during exercise, the higher rates of net lactate formation seen (Fig 13) for chronically-unloaded compared with the loaded muscle is mainly due to the increase in pyruvate production from glycolysis during exercise, similar to what has been seen experimentally [65].

**Figure 14:**
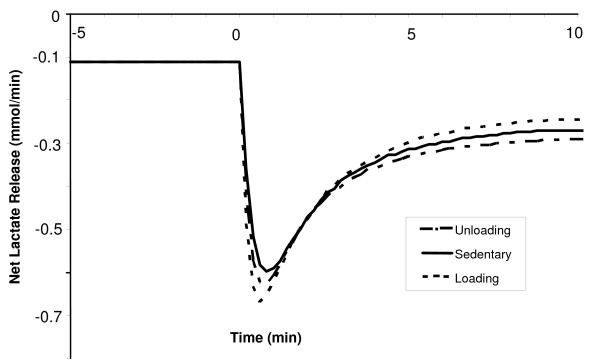
Simulated dynamic responses of the net lactate release from muscle tissue during a 10-min moderate-intensity exercise period (WR = 65W) for sedentary and chronically-loaded/unloaded individuals. Negative values correspond to a net lactate release from skeletal muscle which has bi-phasic behavior.

The lactate production rate in muscle tissue during exercise was seen to have a triphasic behavior. At the onset of exercise, the production of lactate increases rapidly to levels around 5-fold higher than that at rest, followed by a swift drop to resting levels. This transient response occurs within the first minute, and is the same for all conditioned states, as depicted in Figure 13. After that, there is an exponential increase, with the new steady-state values reached by 3 min. The early large production of lactate is consistent with the previous experimental finding by Connett et al. [44]. The steady-state net lactate production in the chronically unloaded muscle is about 20% higher when compared with the loaded muscle. The dynamic responses for net lactate release from muscle tissue during exercise for loading and unloading states are displayed in Figure 14. Initially, the net lactate release (negative of the shown flux) increased rapidly, with largest changes seen in the loading state (0.11 to 0.68 mM/min). In about 45 seconds, the net lactate release increased exponentially in all conditioned states to its new steady-state levels, which were reached in 4 min. The difference in steady-state lactate release values between chronically unloaded and loaded muscles was around 17%, as depicted in Figure 14.

**Figure 15:**
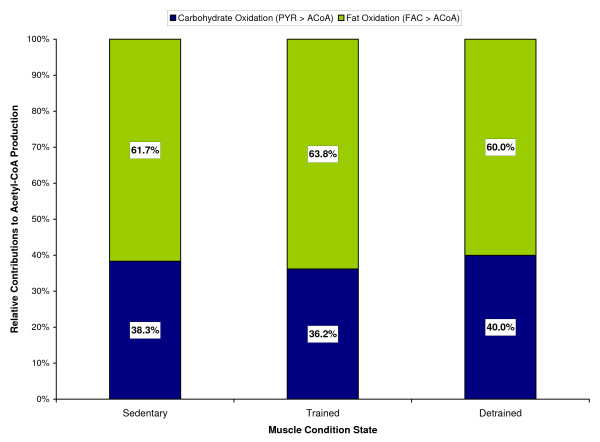
Simulated relative contributions from carbohydrates and fats towards the steady-state acetyl-CoA production during a 10-min moderate-intensity exercise period (WR = 65W) for sedentary and chronically-loaded/unloaded individuals.

The rate of free fatty acid uptake increased exponentially during exercise, with τFFALD
 MathType@MTEF@5@5@+=feaafiart1ev1aaatCvAUfKttLearuWrP9MDH5MBPbIqV92AaeXatLxBI9gBaebbnrfifHhDYfgasaacH8akY=wiFfYdH8Gipec8Eeeu0xXdbba9frFj0=OqFfea0dXdd9vqai=hGuQ8kuc9pgc9s8qqaq=dirpe0xb9q8qiLsFr0=vr0=vr0dc8meaabaqaciaacaGaaeqabaqabeGadaaakeaaiiGacqWFepaDdaqhaaWcbaGaeeOrayKaeeOrayKaeeyqaeeabaGaeeitaWKaeeiraqeaaaaa@3402@ = 0.3 min and τFFAUL
 MathType@MTEF@5@5@+=feaafiart1ev1aaatCvAUfKttLearuWrP9MDH5MBPbIqV92AaeXatLxBI9gBaebbnrfifHhDYfgasaacH8akY=wiFfYdH8Gipec8Eeeu0xXdbba9frFj0=OqFfea0dXdd9vqai=hGuQ8kuc9pgc9s8qqaq=dirpe0xb9q8qiLsFr0=vr0=vr0dc8meaabaqaciaacaGaaeqabaqabeGadaaakeaaiiGacqWFepaDdaqhaaWcbaGaeeOrayKaeeOrayKaeeyqaeeabaGaeeyvauLaeeitaWeaaaaa@3424@ = 0.4 min. The chronically loaded muscle had a 7% higher rate of free fatty acid uptake compared to the unloaded muscle. The steady-state rates of acetyl-CoA production during exercise were similar in all three muscle conditions but the relative contributions differed from carbohydrates and fats. Figure 15 shows the relative contribution towards acetyl-CoA production from carbohydrate and fat oxidations during exercise in sedentary vs. chronically loaded/unloaded muscle. It is seen that carbohydrates have a larger contribution to the formation of acetyl-CoA in unloaded muscle (40% vs. 36%), while fats contribution is higher in chronically loaded muscle (64% vs. 60%). Both chronically loaded and -unloaded muscles at rest have similar tissue respiratory quotients (RQ) of 0.785. However, during exercise, the RQ for loaded muscle rises to 0.827, while the RQ for unloaded muscle increases to 0.836.

## Discussion

In this paper, we successfully extended a previously developed mathematical model of skeletal muscle metabolism [28,29] by adding necessary biochemical components that are altered after periods of chronic loading or unloading to quantify some of the underlying key mechanisms associated with the metabolic responses to constant work rate, moderate-intensity exercise in these loading states. The model was first validated with published experimental data obtained from quadriceps muscle under conditions of moderate-intensity exercise in sedentary individuals. Once validated, the model was used to simulate and predict metabolic responses to moderate-intensity exercise in contracting muscle of individuals who have been subjected to a period of chronic loading or unloading.

### Parallel activation of metabolic pathways during exercise

We conducted extensive computer simulations using this model of skeletal muscle metabolism in response to changes in metabolic rate (simulating work rate changes in the cycle ergometer) to predict changes in metabolite concentrations and reaction flux rates during increased metabolic demand. The aim of these simulations has been to investigate various mechanisms of respiratory control to gain understanding on how the cell adjusts its metabolic machinery and processes to activate enhanced delivery of reducing equivalents (NADH) to the mitochondria for a sudden increase in the rate of ATP synthesis. During voluntary muscle contraction, cellular metabolism is altered and oxygen and fuel delivery is stimulated through adjustments in convection/diffusion reaction processes by the ventilatory, cardiovascular, and muscular systems. Since the current *in vivo *techniques, such as indirect calorimetry, near-infrared spectroscopy, and A-V difference measurements, can only provide information at the pulmonary or muscular level, we are limited in deriving inferences about the metabolic control solely based on this information. Specifically, these techniques can neither provide reliable measurements of the rate of mitochondrial oxygen consumption (VO_2 m_) nor provide a quantitative relationship of the dependence of VO_2 m _on ADP, Pi, NADH and available cellular O_2_, especially at the onset of exercise in an *in vivo *contracting skeletal muscle.

The computational model of skeletal muscle metabolism that we developed permits quantitative evaluation of current hypotheses of pathway activation with increase in work rate (ATP demand). Specifically, we investigated two mechanisms of pathway activation: (i) feedback activation via the products ADP, Pi and NADH and (ii) feedforward activation through parallel activation of each reaction in the pathways of ATP formation. Accordingly, we investigated the effect of increased [ADP] and [Pi] induced by an increased ATP hydrolysis rate – equivalent to a change in work rate (WR) – on the activation of glycogenolysis, glycolysis, pyruvate oxidation, fatty acid oxidation, Krebs cycle, and oxidative phosphorylation. Simulations revealed that the increases in [ADP] and [Pi] were not sufficient to increase the reaction flux rates of the reaction processes involved in providing the corresponding amount of reducing equivalents per unit time to the mitochondria in proportion to the rates of O_2 _utilization and ATP turnover (results not shown). We then investigated the effect of simultaneously increasing maximal reaction velocities (V_max_) of most catalytic reactions participating in energy metabolism in proportion to the increase in metabolic rate (parallel activation as described in the Methods section). Simulations indicated that only by altering simultaneously the V_max _parameters of the reaction flux expressions, it is possible to obtain physiologically meaningful predictions of the metabolic responses to exercise (see Figures 3, 4, 5, 6). Though the exact mechanisms of "parallel activation" have not been clearly elucidated, it may in part be attributed to the stimulation of the activities of mitochondrial dehydrogenases by the levels of free calcium [Ca^2+^] and/or other enzymes by catecholamines [29,34-37], which are in fact activated by neural stimulation [38-40]. The mechanism may also be attributed to other regulatory mechanisms (known or unknown), such as the regulation of TCA cycle and oxidative phosphorylation fluxes through phosphates [76], that have not been accounted for in this phenomenological model of skeletal muscle bioenergetics.

### Muscle oxygen uptake dynamics

Different model-simulated time courses of the increase in muscle oxygen uptake (VO_2_) toward its steady-state in response to a step change in work rate were obtained in chronically-loaded and unloaded muscle (Fig. 8). There is evidence that pulmonary VO_2 _reaches steady-state faster in a well-trained individual than in a sedentary individual at the same absolute work rate [27]. However, at moderate exercise intensities (WR ≤ 40% VO_2 max_), significant differences are not seen between trained or loaded and sedentary subjects until approximately 30 seconds into the exercise bout [27]. Our simulations show similar findings in muscle VO_2 _dynamics, with minor differences between loaded and unloaded responses earlier in the exercise period.

The differences in the dynamics of muscle VO_2 _between loading states at exercise onset may be due to a faster muscle blood flow dynamics and thus increased rate of O_2 _delivery [25,53], or due to a higher oxidative capacity of chronically-loaded muscle [70]. Initially, we hypothesized that the differences in muscle VO_2 _between loading states were due to the faster blood flow response to exercise in chronically-loaded muscle as observed experimentally [53]. The faster delivery of O_2 _to contracting muscle in trained individuals would allow improvements in the transient metabolic responses to exercise, thus permitting a closer coupling between the rates ATP production and ATP utilization and a decreased reliance on anaerobic energy sources such as phosphocreatine and glycogen. A smaller glycogenolytic flux would lead to lower production rates of pyruvate and, ultimately lactate, in chronically loaded muscle.

By performing *in silico *experiments (i.e., computer simulations), it was possible to examine quantitatively the source of the differences in muscle VO_2 _dynamic responses in the three different loading states. By increasing the rate of muscle blood flow at the onset of exercise, with different time profiles but same steady-state value, we could determine if the rate of O_2 _delivery to contracting muscle is limiting. Model simulation showed that, during moderate exercise, regardless of the time profile of blood flow, muscle VO_2 _increased exponentially with similar time constants, τ_VO2_. This suggests that differences in muscle VO_2 _dynamics characteristic of these loading states – during moderate exercise – may be due to cellular adaptations in oxidative machinery (i.e., levels of metabolic controllers and/or enzyme activation) and not to the kinetics of muscle blood flow.

### Phosphorylation ratio differences among muscle loading states during exercise

Various experimental studies have observed smaller increases in [ADP]/[ATP] ratio for trained compared with sedentary individuals during exercise [62,65]. The difference in this ratio between chronically loaded and unloaded muscle in our model is even more drastic (Fig. 9). The increased levels of resting maximal enzyme activity for the oxidative phosphorylation pathway in trained muscle are associated with a greater capacity for increasing reaction velocity and reducing ADP/ATP ratio. In our simulations, the steady-state oxidative phosphorylation flux is the same regardless of the conditioned state of the muscle during moderate-intensity exercise; however, in chronically loaded muscle, a lower ADP/ATP ratio is needed in order to reach this steady-state level. Since this control ratio plays a major role in fine-tuning the flux rates of glycolysis, fat oxidation, and phosphocreatine breakdown, a lower ADP/ATP ratio would result in a tighter coupling of the processes of ATP production and utilization and sparing of fuels and energy stores. For instance, phosphocreatine acts as a buffer to maintain ATP homeostasis and the creatine kinase reaction rate is highly sensitive to changes in ADP/ATP. Simulations showed a smaller decline in ATP concentration and less phosphocreatine breakdown at exercise onset in chronically-loaded muscle during exercise, similar to what is seen experimentally [62,65].

ATP also plays a key role in the activation of both exogenous glucose and fatty acids and in controlling the rates of glycogen synthesis. Glycogen synthase, the enzyme responsible for the synthesis of glycogen from G6P, is activated by ATP. In chronically loaded muscle, there is a lower net glycogen breakdown when compared with unloaded muscle during exercise due to increased rates of glycogen formation as a result of increased ATP levels. A lower rate of glycolysis leads to a lower rate of lactate production as seen in chronically-loaded muscle compared with unloaded muscle during exercise [13,65]. Since lactate concentrations in muscle were similar during moderate exercise regardless of loading state, the increased production of lactate was coupled with higher net release rates in chronically unloaded muscle. This result is in agreement with experimental observations [13,65,77].

### Transient lactate metabolic responses to exercise

A noteworthy observation from our simulations is the transient response of net lactate production during exercise. At the onset of exercise, net lactate production increases dramatically, and then declines rapidly to a value near its resting value before increasing again in an exponential fashion to its elevated steady-state level (Fig. 13). This is a triphasic behavior. The early large production of lactate is consistent with the previous experimental finding by Connett et al. [44]. The reason for this is unknown; however, one explanation may be a delay in the activation of the enzyme lactate dehydrogenase (LDH). The activation of intracellular enzymes occurs at the beginning of exercise, and all reaction fluxes increase proportionately as per parallel activation hypothesis. Lactate and pyruvate are substrates in an equilibrium reaction catalyzed by the enzyme LDH, which is also coupled to the redox ratio NADH/NAD^+^. As exercise starts, NADH drops rapidly to its new steady-state value, causing the LDH reaction to shift towards pyruvate production. As glycolysis increases towards its new steady state, the production of pyruvate from carbohydrates increases which overrides the decrease of NADH, shifting the LDH equilibrium back towards lactate production. A more extensive experimental look at this profile is required to try to determine the causes of the behavior of this transient response, or to refute its existence entirely.

### Acetyl-CoA production during exercise

Increased flux through fat oxidation pathway leads to higher rates of acetyl-CoA production from fatty acids in chronically loaded muscle compared to unloaded muscle during exercise. Chronically unloaded muscle, with its higher flux through glycolytic pathway, produces more pyruvate, which leads to increased levels of acetyl-CoA from carbohydrates than chronically loaded muscle (Fig. 15). Studies have shown that following endurance training, insignificant changes are measured both in the total and active concentrations for pyruvate dehydrogenase (PDH), the enzyme responsible for oxidizing pyruvate to acetyl-CoA [65]. In our model, PDH maximal reaction velocity was the same for all three loading states. Thus, reductions in pyruvate oxidation for chronically loaded muscle during exercise are most likely due to the decreased availability of pyruvate from glycogenolysis and glycolysis. Additional inhibition of this reaction by its product may also play some role, as increased levels of acetyl-CoA from fat oxidation may inhibit its production from carbohydrates. While experimental techniques may not be sensitive enough to measure these fuel differences between chronically-loaded and unloaded states, especially at the exercise intensities performed in these simulations, our model predicts that the respiratory quotients for trained and detrained muscle during moderate-intensity exercise are 0.826 and 0.835, respectively, corresponding to 36% and 40% contributions from carbohydrates towards the production of acetyl-CoA.

### Effect of enzyme activation on reaction flux regulation

We have distinguished the roles of various species and controllers in regulating flux rates through various metabolic pathways (Appendix B). While each species and controller does serve key functions, we feel it is important to emphasize that they are responsible only for fine-tuned regulation. The main regulatory control is achieved through both the alterations in the ratio of active/inactive enzyme molecules and different enzymatic adaptations to training and detraining, as proposed by Hochachka and Matheson [52]. As we have shown earlier, the maximal velocity for each enzymatic reaction in our model is coupled with a relative metabolic rate (RMR) term that increases in parallel with increases in ATP turnover rate. For our simulated exercise bout (WR = 65W), we could induce a 14-fold increase in the ATP hydrolysis rate that we feel is representative of moderate-intensity exercise. In order for us to closely match this increased rate of ATP-utilization, the ATP-producing pathways must increase its flux turnover capacity to similar levels. As was mentioned earlier, it is not possible for this to be achieved strictly through changes in species concentration only (e.g., feedback activation by ADP, Pi and NADH), and thus we felt it was appropriate to increase the V_max _for each reaction in parallel by some factor similar for all states (parallel activation). This approach has been done in other *in silico *research that has attempted to study the regulation of ATP turnover rates during exercise [29,34-37].

### Model limitations and future developments

In this model of skeletal muscle metabolism, the cytosolic and mitochondrial regions were not distinguished. Consequently, for some physiological stresses such as heavy-intensity exercise, the model may not accurately predict the dynamics of several metabolite concentrations and reaction fluxes that are critical in the regulation of cellular respiration and fuel (carbohydrate, fat and lactate) metabolism. For example, the modulators of several key metabolic reactions in cytosol and mitochondria ([ATP]/[ADP] and [NADH]/[NAD^+^]) have different concentrations in these subcellular domains [48]. Another limitation in this model is that most of the lumped enzymatic reactions in the cellular biochemical pathways were considered irreversible in the direction of product formation. In principle, almost all cellular metabolic reactions are essentially reversible [31,33] and are governed by thermodynamic equilibrium conditions [30,50]. Under typical physiological conditions, which are far from the equilibrium, however, most metabolic fluxes are typically dominant in one direction although their magnitudes can change during some pathological conditions.

A more physiologically and biochemically mechanistic model of skeletal muscle metabolism should include the distinction of cytosol and mitochondrial domains within the tissue cells. Also, the subcellular compartmentation could account for distinct regions of metabolite distribution, for example, associated with glycolysis [48]. Furthermore, alternative kinetic flux expressions can be based on Michaelis-Menten formalism for reversible enzymatic reactions satisfying Haldane relationship that apply for thermodynamic equilibrium [30,31,49,50]. This would provide additional thermodynamic constraints on maximal reaction velocities.

## Conclusion

We successfully extended a previously developed model of skeletal muscle metabolism and obtained agreement with experimental data under several different physiological conditions for sedentary muscle. By selecting kinetic model parameter values in agreement with alterations in enzymes contents/activities induced by chronic loading and unloading, we were able to identify differences in fuel preference and delivery of arterial species during moderate-intensity exercise. In particular, we were able to examine – with computer simulations – the impact of cellular metabolic adaptations induced by chronic unloading/loading on the dynamics of various exercise responses. Chronically loaded muscle displayed a faster muscle VO_2 _kinetic response to a step increase in work rate. Unloaded muscle oxidized a larger percentage of carbohydrates for ATP synthesis during exercise. This increase in carbohydrates utilization also leads to higher rates of intramuscular lactate formation. With the proposed enhancements and with additional metabolic pathways similar to those of the model of cardiac metabolism [48], a model of skeletal muscle metabolism could be applied to test complex hypotheses involving the integration of cellular metabolic networks and responses during exercise in individuals who have undergone periods of training (loading) or chronic physical inactivity (unloading).

## Appendix A: Dynamic mass balance equations

**Table 6:** 

Glucose (GLU)	VcdCc,GLUdt=−φGLU→G6P+Q(Ca,GLU−σGLUCc,GLU) MathType@MTEF@5@5@+=feaafiart1ev1aaatCvAUfKttLearuWrP9MDH5MBPbIqV92AaeXatLxBI9gBaebbnrfifHhDYfgasaacH8akY=wiFfYdH8Gipec8Eeeu0xXdbba9frFj0=OqFfea0dXdd9vqai=hGuQ8kuc9pgc9s8qqaq=dirpe0xb9q8qiLsFr0=vr0=vr0dc8meaabaqaciaacaGaaeqabaqabeGadaaakeaacqWGwbGvdaWgaaWcbaGaee4yamgabeaakmaalaaabaGaemizaqMaem4qam0aaSbaaSqaaiabbogaJjabbYcaSiabbEeahjabbYeamjabbwfavbqabaaakeaacqWGKbazcqWG0baDaaGaeyypa0JaeyOeI0ccciGae8NXdy2aaSbaaSqaaiabbEeahjabbYeamjabbwfavjabgkziUkabbEeahjabbAda2iabbcfaqbqabaGccqGHRaWkcqWGrbqucqGGOaakcqWGdbWqdaWgaaWcbaGaeeyyaeMaeeilaWIaee4raCKaeeitaWKaeeyvaufabeaakiabgkHiTiab=n8aZnaaBaaaleaacqqGhbWrcqqGmbatcqqGvbqvaeqaaOGaem4qam0aaSbaaSqaaiabbogaJjabbYcaSiabbEeahjabbYeamjabbwfavbqabaGccqGGPaqkaaa@5E74@
Glucose 6-Phosphate (G6P)	VcdCc,G6Pdt=φGLU→G6P+φGLY→G6P−φG6P→GLY−φG6P→GA3P MathType@MTEF@5@5@+=feaafiart1ev1aaatCvAUfKttLearuWrP9MDH5MBPbIqV92AaeXatLxBI9gBaebbnrfifHhDYfgasaacH8akY=wiFfYdH8Gipec8Eeeu0xXdbba9frFj0=OqFfea0dXdd9vqai=hGuQ8kuc9pgc9s8qqaq=dirpe0xb9q8qiLsFr0=vr0=vr0dc8meaabaqaciaacaGaaeqabaqabeGadaaakeaacqWGwbGvdaWgaaWcbaGaee4yamgabeaakmaalaaabaGaemizaqMaem4qam0aaSbaaSqaaiabbogaJjabbYcaSiabbEeahjabbAda2iabbcfaqbqabaaakeaacqWGKbazcqWG0baDaaGaeyypa0dcciGae8NXdy2aaSbaaSqaaiabbEeahjabbYeamjabbwfavjabgkziUkabbEeahjabbAda2iabbcfaqbqabaGccqGHRaWkcqWFgpGzdaWgaaWcbaGaee4raCKaeeitaWKaeeywaKLaeyOKH4Qaee4raCKaeeOnayJaeeiuaafabeaakiabgkHiTiab=z8aMnaaBaaaleaacqqGhbWrcqqG2aGncqqGqbaucqGHsgIRcqqGhbWrcqqGmbatcqqGzbqwaeqaaOGaeyOeI0Iae8NXdy2aaSbaaSqaaiabbEeahjabbAda2iabbcfaqjabgkziUkabbEeahjabbgeabjabbodaZiabbcfaqbqabaaaaa@6874@
Glycogen (GLY)	VcdCc,GLYdt=φG6P→GLY−φGLY→G6P MathType@MTEF@5@5@+=feaafiart1ev1aaatCvAUfKttLearuWrP9MDH5MBPbIqV92AaeXatLxBI9gBaebbnrfifHhDYfgasaacH8akY=wiFfYdH8Gipec8Eeeu0xXdbba9frFj0=OqFfea0dXdd9vqai=hGuQ8kuc9pgc9s8qqaq=dirpe0xb9q8qiLsFr0=vr0=vr0dc8meaabaqaciaacaGaaeqabaqabeGadaaakeaacqWGwbGvdaWgaaWcbaGaee4yamgabeaakmaalaaabaGaemizaqMaem4qam0aaSbaaSqaaiabbogaJjabbYcaSiabbEeahjabbYeamjabbMfazbqabaaakeaacqWGKbazcqWG0baDaaGaeyypa0dcciGae8NXdy2aaSbaaSqaaiabbEeahjabbAda2iabbcfaqjabgkziUkabbEeahjabbYeamjabbMfazbqabaGccqGHsislcqWFgpGzdaWgaaWcbaGaee4raCKaeeitaWKaeeywaKLaeyOKH4Qaee4raCKaeeOnayJaeeiuaafabeaaaaa@5140@
Glyceraldehyde 3-Phosphate (GA3P)	VcdCc,GA3Pdt=2φG6P→GA3P−φGA3P→BPG MathType@MTEF@5@5@+=feaafiart1ev1aaatCvAUfKttLearuWrP9MDH5MBPbIqV92AaeXatLxBI9gBaebbnrfifHhDYfgasaacH8akY=wiFfYdH8Gipec8Eeeu0xXdbba9frFj0=OqFfea0dXdd9vqai=hGuQ8kuc9pgc9s8qqaq=dirpe0xb9q8qiLsFr0=vr0=vr0dc8meaabaqaciaacaGaaeqabaqabeGadaaakeaacqWGwbGvdaWgaaWcbaGaee4yamgabeaakmaalaaabaGaemizaqMaem4qam0aaSbaaSqaaiabbogaJjabbYcaSiabbEeahjabbgeabjabbodaZiabbcfaqbqabaaakeaacqWGKbazcqWG0baDaaGaeyypa0JaeGOmaidcciGae8NXdy2aaSbaaSqaaiabbEeahjabbAda2iabbcfaqjabgkziUkabbEeahjabbgeabjabbodaZiabbcfaqbqabaGccqGHsislcqWFgpGzdaWgaaWcbaGaee4raCKaeeyqaeKaee4mamJaeeiuaaLaeyOKH4QaeeOqaiKaeeiuaaLaee4raCeabeaaaaa@5499@
1,3-Biphospho glycerate (BPG)	VcdCc,BPGdt=φGA3P→BPG−φBPG→PYR MathType@MTEF@5@5@+=feaafiart1ev1aaatCvAUfKttLearuWrP9MDH5MBPbIqV92AaeXatLxBI9gBaebbnrfifHhDYfgasaacH8akY=wiFfYdH8Gipec8Eeeu0xXdbba9frFj0=OqFfea0dXdd9vqai=hGuQ8kuc9pgc9s8qqaq=dirpe0xb9q8qiLsFr0=vr0=vr0dc8meaabaqaciaacaGaaeqabaqabeGadaaakeaacqWGwbGvdaWgaaWcbaGaee4yamgabeaakmaalaaabaGaemizaqMaem4qam0aaSbaaSqaaiabbogaJjabbYcaSiabbkeacjabbcfaqjabbEeahbqabaaakeaacqWGKbazcqWG0baDaaGaeyypa0dcciGae8NXdy2aaSbaaSqaaiabbEeahjabbgeabjabbodaZiabbcfaqjabgkziUkabbkeacjabbcfaqjabbEeahbqabaGccqGHsislcqWFgpGzdaWgaaWcbaGaeeOqaiKaeeiuaaLaee4raCKaeyOKH4QaeeiuaaLaeeywaKLaeeOuaifabeaaaaa@522D@
Pyruvate (PYR)	VcdCc,PYRdt=φBPG→PYR+φLAC→PYR−φPYR→LAC−φPYR→ALA−φPYR→ACoA+Q(Ca,PYR−σPYRCc,PYR) MathType@MTEF@5@5@+=feaafiart1ev1aaatCvAUfKttLearuWrP9MDH5MBPbIqV92AaeXatLxBI9gBaebbnrfifHhDYfgasaacH8akY=wiFfYdH8Gipec8Eeeu0xXdbba9frFj0=OqFfea0dXdd9vqai=hGuQ8kuc9pgc9s8qqaq=dirpe0xb9q8qiLsFr0=vr0=vr0dc8meaabaqaciaacaGaaeqabaqabeGadaaakeaafaqadeGabaaabaGaemOvay1aaSbaaSqaaiabbogaJbqabaGcdaWcaaqaaiabdsgaKjabdoeadnaaBaaaleaacqqGJbWycqqGSaalcqqGqbaucqqGzbqwcqqGsbGuaeqaaaGcbaGaemizaqMaemiDaqhaaiabg2da9GGaciab=z8aMnaaBaaaleaacqqGcbGqcqqGqbaucqqGhbWrcqGHsgIRcqqGqbaucqqGzbqwcqqGsbGuaeqaaOGaey4kaSIae8NXdy2aaSbaaSqaaiabbYeamjabbgeabjabboeadjabgkziUkabbcfaqjabbMfazjabbkfasbqabaGccqGHsislcqWFgpGzdaWgaaWcbaGaeeiuaaLaeeywaKLaeeOuaiLaeyOKH4QaeeitaWKaeeyqaeKaee4qameabeaakiabgkHiTiab=z8aMnaaBaaaleaacqqGqbaucqqGzbqwcqqGsbGucqGHsgIRcqqGbbqqcqqGmbatcqqGbbqqaeqaaaGcbaGaeyOeI0Iae8NXdy2aaSbaaSqaaiabbcfaqjabbMfazjabbkfasjabgkziUkabbgeabjabboeadjabb+gaVjabbgeabbqabaGccqGHRaWkcqWGrbqucqGGOaakcqWGdbWqdaWgaaWcbaGaeeyyaeMaeeilaWIaeeiuaaLaeeywaKLaeeOuaifabeaakiabgkHiTiab=n8aZnaaBaaaleaacqqGqbaucqqGzbqwcqqGsbGuaeqaaOGaem4qam0aaSbaaSqaaiabbogaJjabbYcaSiabbcfaqjabbMfazjabbkfasbqabaGccqGGPaqkaaaaaa@8DBC@
Lactate (LAC)	VcdCc,LACdt=φPYR→LAC−φLAC→PYR+Q(Ca,LAC−σLACCc,LAC) MathType@MTEF@5@5@+=feaafiart1ev1aaatCvAUfKttLearuWrP9MDH5MBPbIqV92AaeXatLxBI9gBaebbnrfifHhDYfgasaacH8akY=wiFfYdH8Gipec8Eeeu0xXdbba9frFj0=OqFfea0dXdd9vqai=hGuQ8kuc9pgc9s8qqaq=dirpe0xb9q8qiLsFr0=vr0=vr0dc8meaabaqaciaacaGaaeqabaqabeGadaaakeaacqWGwbGvdaWgaaWcbaGaee4yamgabeaakmaalaaabaGaemizaqMaem4qam0aaSbaaSqaaiabbogaJjabbYcaSiabbYeamjabbgeabjabboeadbqabaaakeaacqWGKbazcqWG0baDaaGaeyypa0dcciGae8NXdy2aaSbaaSqaaiabbcfaqjabbMfazjabbkfasjabgkziUkabbYeamjabbgeabjabboeadbqabaGccqGHsislcqWFgpGzdaWgaaWcbaGaeeitaWKaeeyqaeKaee4qamKaeyOKH4QaeeiuaaLaeeywaKLaeeOuaifabeaakiabgUcaRiabdgfarjabcIcaOiabdoeadnaaBaaaleaacqqGHbqycqqGSaalcqqGmbatcqqGbbqqcqqGdbWqaeqaaOGaeyOeI0Iae83Wdm3aaSbaaSqaaiabbYeamjabbgeabjabboeadbqabaGccqWGdbWqdaWgaaWcbaGaee4yamMaeeilaWIaeeitaWKaeeyqaeKaee4qameabeaakiabcMcaPaaa@6877@
Alanine (ALA)	VcdCc,ALAdt=φPYR→ALA+Q(Ca,ALA−σALACc,ALA) MathType@MTEF@5@5@+=feaafiart1ev1aaatCvAUfKttLearuWrP9MDH5MBPbIqV92AaeXatLxBI9gBaebbnrfifHhDYfgasaacH8akY=wiFfYdH8Gipec8Eeeu0xXdbba9frFj0=OqFfea0dXdd9vqai=hGuQ8kuc9pgc9s8qqaq=dirpe0xb9q8qiLsFr0=vr0=vr0dc8meaabaqaciaacaGaaeqabaqabeGadaaakeaacqWGwbGvdaWgaaWcbaGaee4yamgabeaakmaalaaabaGaemizaqMaem4qam0aaSbaaSqaaiabbogaJjabbYcaSiabbgeabjabbYeamjabbgeabbqabaaakeaacqWGKbazcqWG0baDaaGaeyypa0dcciGae8NXdy2aaSbaaSqaaiabbcfaqjabbMfazjabbkfasjabgkziUkabbgeabjabbYeamjabbgeabbqabaGccqGHRaWkcqWGrbqucqGGOaakcqWGdbWqdaWgaaWcbaGaeeyyaeMaeeilaWIaeeyqaeKaeeitaWKaeeyqaeeabeaakiabgkHiTiab=n8aZnaaBaaaleaacqqGbbqqcqqGmbatcqqGbbqqaeqaaOGaem4qam0aaSbaaSqaaiabbogaJjabbYcaSiabbgeabjabbYeamjabbgeabbqabaGccqGGPaqkaaa@5CDF@
Triglycerides (TGL)	VcdCc,TGLdt=φGLC→TGL−φTGL→GLC+Q(Ca,TGL−σTGLCc,TGL) MathType@MTEF@5@5@+=feaafiart1ev1aaatCvAUfKttLearuWrP9MDH5MBPbIqV92AaeXatLxBI9gBaebbnrfifHhDYfgasaacH8akY=wiFfYdH8Gipec8Eeeu0xXdbba9frFj0=OqFfea0dXdd9vqai=hGuQ8kuc9pgc9s8qqaq=dirpe0xb9q8qiLsFr0=vr0=vr0dc8meaabaqaciaacaGaaeqabaqabeGadaaakeaacqWGwbGvdaWgaaWcbaGaee4yamgabeaakmaalaaabaGaemizaqMaem4qam0aaSbaaSqaaiabbogaJjabbYcaSiabbsfaujabbEeahjabbYeambqabaaakeaacqWGKbazcqWG0baDaaGaeyypa0dcciGae8NXdy2aaSbaaSqaaiabbEeahjabbYeamjabboeadjabgkziUkabbsfaujabbEeahjabbYeambqabaGccqGHsislcqWFgpGzdaWgaaWcbaGaeeivaqLaee4raCKaeeitaWKaeyOKH4Qaee4raCKaeeitaWKaee4qameabeaakiabgUcaRiabdgfarjabcIcaOiabdoeadnaaBaaaleaacqqGHbqycqqGSaalcqqGubavcqqGhbWrcqqGmbataeqaaOGaeyOeI0Iae83Wdm3aaSbaaSqaaiabbsfaujabbEeahjabbYeambqabaGccqWGdbWqdaWgaaWcbaGaee4yamMaeeilaWIaeeivaqLaee4raCKaeeitaWeabeaakiabcMcaPaaa@68F7@
Glycerol (GLC)	VcdCc,GLCdt=φTGL→GLC−φGLC→TGL+Q(Ca,GLC−σGLCCc,GLC) MathType@MTEF@5@5@+=feaafiart1ev1aaatCvAUfKttLearuWrP9MDH5MBPbIqV92AaeXatLxBI9gBaebbnrfifHhDYfgasaacH8akY=wiFfYdH8Gipec8Eeeu0xXdbba9frFj0=OqFfea0dXdd9vqai=hGuQ8kuc9pgc9s8qqaq=dirpe0xb9q8qiLsFr0=vr0=vr0dc8meaabaqaciaacaGaaeqabaqabeGadaaakeaacqWGwbGvdaWgaaWcbaGaee4yamgabeaakmaalaaabaGaemizaqMaem4qam0aaSbaaSqaaiabbogaJjabbYcaSiabbEeahjabbYeamjabboeadbqabaaakeaacqWGKbazcqWG0baDaaGaeyypa0dcciGae8NXdy2aaSbaaSqaaiabbsfaujabbEeahjabbYeamjabgkziUkabbEeahjabbYeamjabboeadbqabaGccqGHsislcqWFgpGzdaWgaaWcbaGaee4raCKaeeitaWKaee4qamKaeyOKH4QaeeivaqLaee4raCKaeeitaWeabeaakiabgUcaRiabdgfarjabcIcaOiabdoeadnaaBaaaleaacqqGHbqycqqGSaalcqqGhbWrcqqGmbatcqqGdbWqaeqaaOGaeyOeI0Iae83Wdm3aaSbaaSqaaiabbEeahjabbYeamjabboeadbqabaGccqWGdbWqdaWgaaWcbaGaee4yamMaeeilaWIaee4raCKaeeitaWKaee4qameabeaakiabcMcaPaaa@686F@
Free Fatty Acid (FFA)	VcdCc,FFAdt=3φTGL→GLC−3φGLC→TGL−φFFA→FAC+Q(Ca,FFA−σFFACc,FFA) MathType@MTEF@5@5@+=feaafiart1ev1aaatCvAUfKttLearuWrP9MDH5MBPbIqV92AaeXatLxBI9gBaebbnrfifHhDYfgasaacH8akY=wiFfYdH8Gipec8Eeeu0xXdbba9frFj0=OqFfea0dXdd9vqai=hGuQ8kuc9pgc9s8qqaq=dirpe0xb9q8qiLsFr0=vr0=vr0dc8meaabaqaciaacaGaaeqabaqabeGadaaakeaacqWGwbGvdaWgaaWcbaGaee4yamgabeaakmaalaaabaGaemizaqMaem4qam0aaSbaaSqaaiabbogaJjabbYcaSiabbAeagjabbAeagjabbgeabbqabaaakeaacqWGKbazcqWG0baDaaGaeyypa0JaeG4mamdcciGae8NXdy2aaSbaaSqaaiabbsfaujabbEeahjabbYeamjabgkziUkabbEeahjabbYeamjabboeadbqabaGccqGHsislcqaIZaWmcqWFgpGzdaWgaaWcbaGaee4raCKaeeitaWKaee4qamKaeyOKH4QaeeivaqLaee4raCKaeeitaWeabeaakiabgkHiTiab=z8aMnaaBaaaleaacqqGgbGrcqqGgbGrcqqGbbqqcqGHsgIRcqqGgbGrcqqGbbqqcqqGdbWqaeqaaOGaey4kaSIaemyuaeLaeiikaGIaem4qam0aaSbaaSqaaiabbggaHjabbYcaSiabbAeagjabbAeagjabbgeabbqabaGccqGHsislcqWFdpWCdaWgaaWcbaGaeeOrayKaeeOrayKaeeyqaeeabeaakiabdoeadnaaBaaaleaacqqGJbWycqqGSaalcqqGgbGrcqqGgbGrcqqGbbqqaeqaaOGaeiykaKcaaa@752B@
Fatty Acyl-CoA (FAC)	VcdCc,FACdt=φFFA→FAC−φFAC→ACoA MathType@MTEF@5@5@+=feaafiart1ev1aaatCvAUfKttLearuWrP9MDH5MBPbIqV92AaeXatLxBI9gBaebbnrfifHhDYfgasaacH8akY=wiFfYdH8Gipec8Eeeu0xXdbba9frFj0=OqFfea0dXdd9vqai=hGuQ8kuc9pgc9s8qqaq=dirpe0xb9q8qiLsFr0=vr0=vr0dc8meaabaqaciaacaGaaeqabaqabeGadaaakeaacqWGwbGvdaWgaaWcbaGaee4yamgabeaakmaalaaabaGaemizaqMaem4qam0aaSbaaSqaaiabbogaJjabbYcaSiabbAeagjabbgeabjabboeadbqabaaakeaacqWGKbazcqWG0baDaaGaeyypa0dcciGae8NXdy2aaSbaaSqaaiabbAeagjabbAeagjabbgeabjabgkziUkabbAeagjabbgeabjabboeadbqabaGccqGHsislcqWFgpGzdaWgaaWcbaGaeeOrayKaeeyqaeKaee4qamKaeyOKH4QaeeyqaeKaee4qamKaee4Ba8Maeeyqaeeabeaaaaa@51C9@
Acetyl-CoA (ACoA)	VcdCc,ACoAdt=φPYR→ACoA+8φFAC→ACoA−φACoA→CIT MathType@MTEF@5@5@+=feaafiart1ev1aaatCvAUfKttLearuWrP9MDH5MBPbIqV92AaeXatLxBI9gBaebbnrfifHhDYfgasaacH8akY=wiFfYdH8Gipec8Eeeu0xXdbba9frFj0=OqFfea0dXdd9vqai=hGuQ8kuc9pgc9s8qqaq=dirpe0xb9q8qiLsFr0=vr0=vr0dc8meaabaqaciaacaGaaeqabaqabeGadaaakeaacqWGwbGvdaWgaaWcbaGaee4yamgabeaakmaalaaabaGaemizaqMaem4qam0aaSbaaSqaaiabbogaJjabbYcaSiabbgeabjabboeadjabb+gaVjabbgeabbqabaaakeaacqWGKbazcqWG0baDaaGaeyypa0dcciGae8NXdy2aaSbaaSqaaiabbcfaqjabbMfazjabbkfasjabgkziUkabbgeabjabboeadjabb+gaVjabbgeabbqabaGccqGHRaWkcqaI4aaocqWFgpGzdaWgaaWcbaGaeeOrayKaeeyqaeKaee4qamKaeyOKH4QaeeyqaeKaee4qamKaee4Ba8MaeeyqaeeabeaakiabgkHiTiab=z8aMnaaBaaaleaacqqGbbqqcqqGdbWqcqqGVbWBcqqGbbqqcqGHsgIRcqqGdbWqcqqGjbqscqqGubavaeqaaaaa@626B@
Citrate (CIT)	VcdCc,CITdt=φACoA→CIT−φCIT→AKG MathType@MTEF@5@5@+=feaafiart1ev1aaatCvAUfKttLearuWrP9MDH5MBPbIqV92AaeXatLxBI9gBaebbnrfifHhDYfgasaacH8akY=wiFfYdH8Gipec8Eeeu0xXdbba9frFj0=OqFfea0dXdd9vqai=hGuQ8kuc9pgc9s8qqaq=dirpe0xb9q8qiLsFr0=vr0=vr0dc8meaabaqaciaacaGaaeqabaqabeGadaaakeaacqWGwbGvdaWgaaWcbaGaee4yamgabeaakmaalaaabaGaemizaqMaem4qam0aaSbaaSqaaiabbogaJjabbYcaSiabboeadjabbMeajjabbsfaubqabaaakeaacqWGKbazcqWG0baDaaGaeyypa0dcciGae8NXdy2aaSbaaSqaaiabbgeabjabboeadjabb+gaVjabbgeabjabgkziUkabboeadjabbMeajjabbsfaubqabaGccqGHsislcqWFgpGzdaWgaaWcbaGaee4qamKaeeysaKKaeeivaqLaeyOKH4QaeeyqaeKaee4saSKaee4raCeabeaaaaa@5259@
α-Ketoglutarate (AKG)	VcdCc,AKGdt=φCIT→AKG−φAKG→SCoA MathType@MTEF@5@5@+=feaafiart1ev1aaatCvAUfKttLearuWrP9MDH5MBPbIqV92AaeXatLxBI9gBaebbnrfifHhDYfgasaacH8akY=wiFfYdH8Gipec8Eeeu0xXdbba9frFj0=OqFfea0dXdd9vqai=hGuQ8kuc9pgc9s8qqaq=dirpe0xb9q8qiLsFr0=vr0=vr0dc8meaabaqaciaacaGaaeqabaqabeGadaaakeaacqWGwbGvdaWgaaWcbaGaee4yamgabeaakmaalaaabaGaemizaqMaem4qam0aaSbaaSqaaiabbogaJjabbYcaSiabbgeabjabbUealjabbEeahbqabaaakeaacqWGKbazcqWG0baDaaGaeyypa0dcciGae8NXdy2aaSbaaSqaaiabboeadjabbMeajjabbsfaujabgkziUkabbgeabjabbUealjabbEeahbqabaGccqGHsislcqWFgpGzdaWgaaWcbaGaeeyqaeKaee4saSKaee4raCKaeyOKH4Qaee4uamLaee4qamKaee4Ba8Maeeyqaeeabeaaaaa@5249@
Succinyl-CoA (SCoA)	VcdCc,SCoAdt=φAKG→SCoA−φSCoA→SUC MathType@MTEF@5@5@+=feaafiart1ev1aaatCvAUfKttLearuWrP9MDH5MBPbIqV92AaeXatLxBI9gBaebbnrfifHhDYfgasaacH8akY=wiFfYdH8Gipec8Eeeu0xXdbba9frFj0=OqFfea0dXdd9vqai=hGuQ8kuc9pgc9s8qqaq=dirpe0xb9q8qiLsFr0=vr0=vr0dc8meaabaqaciaacaGaaeqabaqabeGadaaakeaacqWGwbGvdaWgaaWcbaGaee4yamgabeaakmaalaaabaGaemizaqMaem4qam0aaSbaaSqaaiabbogaJjabbYcaSiabbofatjabboeadjabb+gaVjabbgeabbqabaaakeaacqWGKbazcqWG0baDaaGaeyypa0dcciGae8NXdy2aaSbaaSqaaiabbgeabjabbUealjabbEeahjabgkziUkabbofatjabboeadjabb+gaVjabbgeabbqabaGccqGHsislcqWFgpGzdaWgaaWcbaGaee4uamLaee4qamKaee4Ba8MaeeyqaeKaeyOKH4Qaee4uamLaeeyvauLaee4qameabeaaaaa@5539@
Succinate (SUC)	VcdCc,SUCdt=φSCoA→SUC−φSUC→MAL MathType@MTEF@5@5@+=feaafiart1ev1aaatCvAUfKttLearuWrP9MDH5MBPbIqV92AaeXatLxBI9gBaebbnrfifHhDYfgasaacH8akY=wiFfYdH8Gipec8Eeeu0xXdbba9frFj0=OqFfea0dXdd9vqai=hGuQ8kuc9pgc9s8qqaq=dirpe0xb9q8qiLsFr0=vr0=vr0dc8meaabaqaciaacaGaaeqabaqabeGadaaakeaacqWGwbGvdaWgaaWcbaGaee4yamgabeaakmaalaaabaGaemizaqMaem4qam0aaSbaaSqaaiabbogaJjabbYcaSiabbofatjabbwfavjabboeadbqabaaakeaacqWGKbazcqWG0baDaaGaeyypa0dcciGae8NXdy2aaSbaaSqaaiabbofatjabboeadjabb+gaVjabbgeabjabgkziUkabbofatjabbwfavjabboeadbqabaGccqGHsislcqWFgpGzdaWgaaWcbaGaee4uamLaeeyvauLaee4qamKaeyOKH4Qaeeyta0KaeeyqaeKaeeitaWeabeaaaaa@52CD@
Malate (MAL)	VcdCc,MALdt=φSUC→MAL−φMAL→OXA MathType@MTEF@5@5@+=feaafiart1ev1aaatCvAUfKttLearuWrP9MDH5MBPbIqV92AaeXatLxBI9gBaebbnrfifHhDYfgasaacH8akY=wiFfYdH8Gipec8Eeeu0xXdbba9frFj0=OqFfea0dXdd9vqai=hGuQ8kuc9pgc9s8qqaq=dirpe0xb9q8qiLsFr0=vr0=vr0dc8meaabaqaciaacaGaaeqabaqabeGadaaakeaacqWGwbGvdaWgaaWcbaGaee4yamgabeaakmaalaaabaGaemizaqMaem4qam0aaSbaaSqaaiabbogaJjabbYcaSiabb2eanjabbgeabjabbYeambqabaaakeaacqWGKbazcqWG0baDaaGaeyypa0dcciGae8NXdy2aaSbaaSqaaiabbofatjabbwfavjabboeadjabgkziUkabb2eanjabbgeabjabbYeambqabaGccqGHsislcqWFgpGzdaWgaaWcbaGaeeyta0KaeeyqaeKaeeitaWKaeyOKH4Qaee4ta8KaeeiwaGLaeeyqaeeabeaaaaa@5146@
Oxaloacetate (OXA)	VcdCc,OXAdt=φMAL→OXA−φACoA→CIT MathType@MTEF@5@5@+=feaafiart1ev1aaatCvAUfKttLearuWrP9MDH5MBPbIqV92AaeXatLxBI9gBaebbnrfifHhDYfgasaacH8akY=wiFfYdH8Gipec8Eeeu0xXdbba9frFj0=OqFfea0dXdd9vqai=hGuQ8kuc9pgc9s8qqaq=dirpe0xb9q8qiLsFr0=vr0=vr0dc8meaabaqaciaacaGaaeqabaqabeGadaaakeaacqWGwbGvdaWgaaWcbaGaee4yamgabeaakmaalaaabaGaemizaqMaem4qam0aaSbaaSqaaiabbogaJjabbYcaSiabb+eapjabbIfayjabbgeabbqabaaakeaacqWGKbazcqWG0baDaaGaeyypa0dcciGae8NXdy2aaSbaaSqaaiabb2eanjabbgeabjabbYeamjabgkziUkabb+eapjabbIfayjabbgeabbqabaGccqGHsislcqWFgpGzdaWgaaWcbaGaeeyqaeKaee4qamKaee4Ba8MaeeyqaeKaeyOKH4Qaee4qamKaeeysaKKaeeivaqfabeaaaaa@5287@
CO2	VcdCc,CO2dt=φPYR→ACoA+φCIT→AKG+φAKG→SCoA+Q(Ca,CO2−σCO2Cc,CO2) MathType@MTEF@5@5@+=feaafiart1ev1aaatCvAUfKttLearuWrP9MDH5MBPbIqV92AaeXatLxBI9gBaebbnrfifHhDYfgasaacH8akY=wiFfYdH8Gipec8Eeeu0xXdbba9frFj0=OqFfea0dXdd9vqai=hGuQ8kuc9pgc9s8qqaq=dirpe0xb9q8qiLsFr0=vr0=vr0dc8meaabaqaciaacaGaaeqabaqabeGadaaakeaacqWGwbGvdaWgaaWcbaGaee4yamgabeaakmaalaaabaGaemizaqMaem4qam0aaSbaaSqaaiabbogaJjabbYcaSiabboeadjabb+eapjabbkdaYaqabaaakeaacqWGKbazcqWG0baDaaGaeyypa0dcciGae8NXdy2aaSbaaSqaaiabbcfaqjabbMfazjabbkfasjabgkziUkabbgeabjabboeadjabb+gaVjabbgeabbqabaGccqGHRaWkcqWFgpGzdaWgaaWcbaGaee4qamKaeeysaKKaeeivaqLaeyOKH4QaeeyqaeKaee4saSKaee4raCeabeaakiabgUcaRGGaaiab+z8aMnaaBaaaleaacqqGbbqqcqqGlbWscqqGhbWrcqGHsgIRcqqGtbWucqqGdbWqcqqGVbWBcqqGbbqqaeqaaOGaey4kaSIaemyuaeLaeiikaGIaem4qam0aaSbaaSqaaiabbggaHjabbYcaSiabboeadjabb+eapjabbkdaYaqabaGccqGHsislcqWFdpWCdaWgaaWcbaGaee4qamKaee4ta8KaeeOmaidabeaakiabdoeadnaaBaaaleaacqqGJbWycqqGSaalcqqGdbWqcqqGpbWtcqqGYaGmaeqaaOGaeiykaKcaaa@75D0@
O2	VcdCc,O2dt=−φO2→H2O+Q(Ca,O2−σO2Cc,O2) MathType@MTEF@5@5@+=feaafiart1ev1aaatCvAUfKttLearuWrP9MDH5MBPbIqV92AaeXatLxBI9gBaebbnrfifHhDYfgasaacH8akY=wiFfYdH8Gipec8Eeeu0xXdbba9frFj0=OqFfea0dXdd9vqai=hGuQ8kuc9pgc9s8qqaq=dirpe0xb9q8qiLsFr0=vr0=vr0dc8meaabaqaciaacaGaaeqabaqabeGadaaakeaacqWGwbGvdaWgaaWcbaGaee4yamgabeaakmaalaaabaGaemizaqMaem4qam0aaSbaaSqaaiabbogaJjabbYcaSiabb+eapjabbkdaYaqabaaakeaacqWGKbazcqWG0baDaaGaeyypa0JaeyOeI0ccciGae8NXdy2aaSbaaSqaaiabb+eapjabbkdaYiabgkziUkabbIeaijabbkdaYiabb+eapbqabaGccqGHRaWkcqWGrbqucqGGOaakcqWGdbWqdaWgaaWcbaGaeeyyaeMaeeilaWIaee4ta8KaeeOmaidabeaakiabgkHiTiab=n8aZnaaBaaaleaacqqGpbWtcqqGYaGmaeqaaOGaem4qam0aaSbaaSqaaiabbogaJjabbYcaSiabb+eapjabbkdaYaqabaGccqGGPaqkaaa@57C3@
PCr	VcdCc,PCRdt=φCR→PCR−φPCR→CR MathType@MTEF@5@5@+=feaafiart1ev1aaatCvAUfKttLearuWrP9MDH5MBPbIqV92AaeXatLxBI9gBaebbnrfifHhDYfgasaacH8akY=wiFfYdH8Gipec8Eeeu0xXdbba9frFj0=OqFfea0dXdd9vqai=hGuQ8kuc9pgc9s8qqaq=dirpe0xb9q8qiLsFr0=vr0=vr0dc8meaabaqaciaacaGaaeqabaqabeGadaaakeaacqWGwbGvdaWgaaWcbaGaee4yamgabeaakmaalaaabaGaemizaqMaem4qam0aaSbaaSqaaiabbogaJjabbYcaSiabbcfaqjabboeadjabbkfasbqabaaakeaacqWGKbazcqWG0baDaaGaeyypa0dcciGae8NXdy2aaSbaaSqaaiabboeadjabbkfasjabgkziUkabbcfaqjabboeadjabbkfasbqabaGccqGHsislcqWFgpGzdaWgaaWcbaGaeeiuaaLaee4qamKaeeOuaiLaeyOKH4Qaee4qamKaeeOuaifabeaaaaa@4F28@
Cr	VcdCc,CRdt=φPCR→CR−φCR→PCR MathType@MTEF@5@5@+=feaafiart1ev1aaatCvAUfKttLearuWrP9MDH5MBPbIqV92AaeXatLxBI9gBaebbnrfifHhDYfgasaacH8akY=wiFfYdH8Gipec8Eeeu0xXdbba9frFj0=OqFfea0dXdd9vqai=hGuQ8kuc9pgc9s8qqaq=dirpe0xb9q8qiLsFr0=vr0=vr0dc8meaabaqaciaacaGaaeqabaqabeGadaaakeaacqWGwbGvdaWgaaWcbaGaee4yamgabeaakmaalaaabaGaemizaqMaem4qam0aaSbaaSqaaiabbogaJjabbYcaSiabboeadjabbkfasbqabaaakeaacqWGKbazcqWG0baDaaGaeyypa0dcciGae8NXdy2aaSbaaSqaaiabbcfaqjabboeadjabbkfasjabgkziUkabboeadjabbkfasbqabaGccqGHsislcqWFgpGzdaWgaaWcbaGaee4qamKaeeOuaiLaeyOKH4QaeeiuaaLaee4qamKaeeOuaifabeaaaaa@4E01@
Pi	VcdCc,Pidt=2φG6P→GLY+7φGLC→TGL+2φFFA→FAC+φATP→ADP−φGLY→G6P−φGA3P→BPG−φSCoA→SUC−5.64φO2→H2O MathType@MTEF@5@5@+=feaafiart1ev1aaatCvAUfKttLearuWrP9MDH5MBPbIqV92AaeXatLxBI9gBaebbnrfifHhDYfgasaacH8akY=wiFfYdH8Gipec8Eeeu0xXdbba9frFj0=OqFfea0dXdd9vqai=hGuQ8kuc9pgc9s8qqaq=dirpe0xb9q8qiLsFr0=vr0=vr0dc8meaabaqaciaacaGaaeqabaqabeGadaaakeaafaqadeGabaaabaGaemOvay1aaSbaaSqaaiabbogaJbqabaGcdaWcaaqaaiabdsgaKjabdoeadnaaBaaaleaacqqGJbWycqqGSaalcqqGqbaucqqGPbqAaeqaaaGcbaGaemizaqMaemiDaqhaaiabg2da9iabikdaYGGaciab=z8aMnaaBaaaleaacqqGhbWrcqqG2aGncqqGqbaucqGHsgIRcqqGhbWrcqqGmbatcqqGzbqwaeqaaOGaey4kaSIaeG4naCJae8NXdy2aaSbaaSqaaiabbEeahjabbYeamjabboeadjabgkziUkabbsfaujabbEeahjabbYeambqabaGccqGHRaWkcqaIYaGmcqWFgpGzdaWgaaWcbaGaeeOrayKaeeOrayKaeeyqaeKaeyOKH4QaeeOrayKaeeyqaeKaee4qameabeaakiabgUcaRiab=z8aMnaaBaaaleaacqqGbbqqcqqGubavcqqGqbaucqGHsgIRcqqGbbqqcqqGebarcqqGqbauaeqaaOGaeyOeI0Iae8NXdy2aaSbaaSqaaiabbEeahjabbYeamjabbMfazjabgkziUkabbEeahjabbAda2iabbcfaqbqabaaakeaacqGHsislcqWFgpGzdaWgaaWcbaGaee4raCKaeeyqaeKaee4mamJaeeiuaaLaeyOKH4QaeeOqaiKaeeiuaaLaee4raCeabeaakiabgkHiTiab=z8aMnaaBaaaleaacqqGtbWucqqGdbWqcqqGVbWBcqqGbbqqcqGHsgIRcqqGtbWucqqGvbqvcqqGdbWqaeqaaOGaeyOeI0IaeGynauJaeiOla4IaeGOnayJaeGinaqJae8NXdy2aaSbaaSqaaiabb+eapjabbkdaYiabgkziUkabbIeaijabbkdaYiabb+eapbqabaaaaaaa@9BE2@
CoA	VcdCc,CoAdt=φACoA→CIT+φSCoA→SUC−φPYR→ACoA−φFFA→FAC−7φFAC→ACoA−φAKG→SCoA MathType@MTEF@5@5@+=feaafiart1ev1aaatCvAUfKttLearuWrP9MDH5MBPbIqV92AaeXatLxBI9gBaebbnrfifHhDYfgasaacH8akY=wiFfYdH8Gipec8Eeeu0xXdbba9frFj0=OqFfea0dXdd9vqai=hGuQ8kuc9pgc9s8qqaq=dirpe0xb9q8qiLsFr0=vr0=vr0dc8meaabaqaciaacaGaaeqabaqabeGadaaakeaafaqadeGabaaabaGaemOvay1aaSbaaSqaaiabbogaJbqabaGcdaWcaaqaaiabdsgaKjabdoeadnaaBaaaleaacqqGJbWycqqGSaalcqqGdbWqcqqGVbWBcqqGbbqqaeqaaaGcbaGaemizaqMaemiDaqhaaiabg2da9GGaciab=z8aMnaaBaaaleaacqqGbbqqcqqGdbWqcqqGVbWBcqqGbbqqcqGHsgIRcqqGdbWqcqqGjbqscqqGubavaeqaaOGaey4kaSIae8NXdy2aaSbaaSqaaiabbofatjabboeadjabb+gaVjabbgeabjabgkziUkabbofatjabbwfavjabboeadbqabaGccqGHsislcqWFgpGzdaWgaaWcbaGaeeiuaaLaeeywaKLaeeOuaiLaeyOKH4QaeeyqaeKaee4qamKaee4Ba8MaeeyqaeeabeaakiabgkHiTiab=z8aMnaaBaaaleaacqqGgbGrcqqGgbGrcqqGbbqqcqGHsgIRcqqGgbGrcqqGbbqqcqqGdbWqaeqaaOGaeyOeI0IaeG4naCJae8NXdy2aaSbaaSqaaiabbAeagjabbgeabjabboeadjabgkziUkabbgeabjabboeadjabb+gaVjabbgeabbqabaaakeaacqGHsislcqWFgpGzdaWgaaWcbaGaeeyqaeKaee4saSKaee4raCKaeyOKH4Qaee4uamLaee4qamKaee4Ba8Maeeyqaeeabeaaaaaaaa@8609@
NADH	VcdCc,NADHdt=φGA3P→BPG+φLAC→PYR+φPYR→ACoA+353φFAC→ACoA+φCIT→AKG+φAKG→SCoA+23φSUC→MAL+φMAL→OXA−φPYR→LAC−1.88φO2→H2O MathType@MTEF@5@5@+=feaafiart1ev1aaatCvAUfKttLearuWrP9MDH5MBPbIqV92AaeXatLxBI9gBaebbnrfifHhDYfgasaacH8akY=wiFfYdH8Gipec8Eeeu0xXdbba9frFj0=OqFfea0dXdd9vqai=hGuQ8kuc9pgc9s8qqaq=dirpe0xb9q8qiLsFr0=vr0=vr0dc8meaabaqaciaacaGaaeqabaqabeGadaaakeaafaqadeGabaaabaGaemOvay1aaSbaaSqaaiabbogaJbqabaGcdaWcaaqaaiabdsgaKjabdoeadnaaBaaaleaacqqGJbWycqqGSaalcqqGobGtcqqGbbqqcqqGebarcqqGibasaeqaaaGcbaGaemizaqMaemiDaqhaaiabg2da9GGaciab=z8aMnaaBaaaleaacqqGhbWrcqqGbbqqcqqGZaWmcqqGqbaucqGHsgIRcqqGcbGqcqqGqbaucqqGhbWraeqaaOGaey4kaSIae8NXdy2aaSbaaSqaaiabbYeamjabbgeabjabboeadjabgkziUkabbcfaqjabbMfazjabbkfasbqabaGccqGHRaWkiiaacqGFgpGzdaWgaaWcbaGaeeiuaaLaeeywaKLaeeOuaiLaeyOKH4QaeeyqaeKaee4qamKaee4Ba8MaeeyqaeeabeaakiabgUcaRmaaleaajeaWbaGaeG4mamJaeGynaudabaGaeG4mamdaaOGae8NXdy2aaSbaaSqaaiabbAeagjabbgeabjabboeadjabgkziUkabbgeabjabboeadjabb+gaVjabbgeabbqabaGccqGHRaWkcqWFgpGzdaWgaaWcbaGaee4qamKaeeysaKKaeeivaqLaeyOKH4QaeeyqaeKaee4saSKaee4raCeabeaaaOqaaiabgUcaRiab=z8aMnaaBaaaleaacqqGbbqqcqqGlbWscqqGhbWrcqGHsgIRcqqGtbWucqqGdbWqcqqGVbWBcqqGbbqqaeqaaOGaey4kaSYaaSqaaKqaahaacqaIYaGmaeaacqaIZaWmaaGccqWFgpGzdaWgaaWcbaGaee4uamLaeeyvauLaee4qamKaeyOKH4Qaeeyta0KaeeyqaeKaeeitaWeabeaakiabgUcaRiab=z8aMnaaBaaaleaacqqGnbqtcqqGbbqqcqqGmbatcqGHsgIRcqqGpbWtcqqGybawcqqGbbqqaeqaaOGaeyOeI0Iae8NXdy2aaSbaaSqaaiabbcfaqjabbMfazjabbkfasjabgkziUkabbYeamjabbgeabjabboeadbqabaGccqGHsislcqaIXaqmcqGGUaGlcqaI4aaocqaI4aaocqWFgpGzdaWgaaWcbaGaee4ta8KaeeOmaiJaeyOKH4QaeeisaGKaeeOmaiJaee4ta8eabeaaaaaaaa@BAF9@
NAD+	VcdCc,NAD+dt=φPYR→LAC+1.88φO2→H2O−φGA3P→BPG−φLAC→PYR−φPYR→ACoA−353φFAC→ACoA−φCIT→AKG−φAKG→SUC−23φSUC→MAL−φMAL→OXA MathType@MTEF@5@5@+=feaafiart1ev1aaatCvAUfKttLearuWrP9MDH5MBPbIqV92AaeXatLxBI9gBaebbnrfifHhDYfgasaacH8akY=wiFfYdH8Gipec8Eeeu0xXdbba9frFj0=OqFfea0dXdd9vqai=hGuQ8kuc9pgc9s8qqaq=dirpe0xb9q8qiLsFr0=vr0=vr0dc8meaabaqaciaacaGaaeqabaqabeGadaaakeaafaqadeGabaaabaGaemOvay1aaSbaaSqaaiabbogaJbqabaGcdaWcaaqaaiabdsgaKjabdoeadnaaBaaaleaacqqGJbWycqqGSaalcqqGobGtcqqGbbqqcqqGebarcqqGRaWkaeqaaaGcbaGaemizaqMaemiDaqhaaiabg2da9GGaciab=z8aMnaaBaaaleaacqqGqbaucqqGzbqwcqqGsbGucqGHsgIRcqqGmbatcqqGbbqqcqqGdbWqaeqaaOGaey4kaSIaeGymaeJaeiOla4IaeGioaGJaeGioaGJae8NXdy2aaSbaaSqaaiabb+eapjabbkdaYiabgkziUkabbIeaijabbkdaYiabb+eapbqabaGccqGHsislcqWFgpGzdaWgaaWcbaGaee4raCKaeeyqaeKaee4mamJaeeiuaaLaeyOKH4QaeeOqaiKaeeiuaaLaee4raCeabeaakiabgkHiTiab=z8aMnaaBaaaleaacqqGmbatcqqGbbqqcqqGdbWqcqGHsgIRcqqGqbaucqqGzbqwcqqGsbGuaeqaaOGaeyOeI0Iae8NXdy2aaSbaaSqaaiabbcfaqjabbMfazjabbkfasjabgkziUkabbgeabjabboeadjabb+gaVjabbgeabbqabaaakeaacqGHsisldaWcbaqcbaCaaiabiodaZiabiwda1aqaaiabiodaZaaakiab=z8aMnaaBaaaleaacqqGgbGrcqqGbbqqcqqGdbWqcqGHsgIRcqqGbbqqcqqGdbWqcqqGVbWBcqqGbbqqaeqaaOGaeyOeI0Iae8NXdy2aaSbaaSqaaiabboeadjabbMeajjabbsfaujabgkziUkabbgeabjabbUealjabbEeahbqabaGccqGHsislcqWFgpGzdaWgaaWcbaGaeeyqaeKaee4saSKaee4raCKaeyOKH4Qaee4uamLaeeyvauLaee4qameabeaakiabgkHiTmaaleaajeaWbaGaeGOmaidabaGaeG4mamdaaOGae8NXdy2aaSbaaSqaaiabbofatjabbwfavjabboeadjabgkziUkabb2eanjabbgeabjabbYeambqabaGccqGHsislcqWFgpGzdaWgaaWcbaGaeeyta0KaeeyqaeKaeeitaWKaeyOKH4Qaee4ta8KaeeiwaGLaeeyqaeeabeaaaaaaaa@B9BB@
ATP	VcdCc,ATPdt=2φBPG→PYR+φSCoA→SUC+5.64φO2→H2O+φPCR→CR+φADP→AMP−φGLU→G6P−φG6P→GLY−φG6P→GA3P−7φGLC→TGL−2φFFA→FAC−φCR→PCR−φATP→ADP−φAMP→ADP MathType@MTEF@5@5@+=feaafiart1ev1aaatCvAUfKttLearuWrP9MDH5MBPbIqV92AaeXatLxBI9gBaebbnrfifHhDYfgasaacH8akY=wiFfYdH8Gipec8Eeeu0xXdbba9frFj0=OqFfea0dXdd9vqai=hGuQ8kuc9pgc9s8qqaq=dirpe0xb9q8qiLsFr0=vr0=vr0dc8meaabaqaciaacaGaaeqabaqabeGadaaakeaafaqadeWabaaabaGaemOvay1aaSbaaSqaaiabbogaJbqabaGcdaWcaaqaaiabdsgaKjabdoeadnaaBaaaleaacqqGJbWycqqGSaalcqqGbbqqcqqGubavcqqGqbauaeqaaaGcbaGaemizaqMaemiDaqhaaiabg2da9iabikdaYGGaciab=z8aMnaaBaaaleaacqqGcbGqcqqGqbaucqqGhbWrcqGHsgIRcqqGqbaucqqGzbqwcqqGsbGuaeqaaOGaey4kaSIae8NXdy2aaSbaaSqaaiabbofatjabboeadjabb+gaVjabbgeabjabgkziUkabbofatjabbwfavjabboeadbqabaGccqGHRaWkcqaI1aqncqGGUaGlcqaI2aGncqaI0aancqWFgpGzdaWgaaWcbaGaee4ta8KaeeOmaiJaeyOKH4QaeeisaGKaeeOmaiJaee4ta8eabeaakiabgUcaRiab=z8aMnaaBaaaleaacqqGqbaucqqGdbWqcqqGsbGucqGHsgIRcqqGdbWqcqqGsbGuaeqaaOGaey4kaSIae8NXdy2aaSbaaSqaaiabbgeabjabbseaejabbcfaqjabgkziUkabbgeabjabb2eanjabbcfaqbqabaaakeaacqGHsislcqWFgpGzdaWgaaWcbaGaee4raCKaeeitaWKaeeyvauLaeyOKH4Qaee4raCKaeeOnayJaeeiuaafabeaakiabgkHiTiab=z8aMnaaBaaaleaacqqGhbWrcqqG2aGncqqGqbaucqGHsgIRcqqGhbWrcqqGmbatcqqGzbqwaeqaaOGaeyOeI0Iae8NXdy2aaSbaaSqaaiabbEeahjabbAda2iabbcfaqjabgkziUkabbEeahjabbgeabjabbodaZiabbcfaqbqabaGccqGHsislcqaI3aWncqWFgpGzdaWgaaWcbaGaee4raCKaeeitaWKaee4qamKaeyOKH4QaeeivaqLaee4raCKaeeitaWeabeaakiabgkHiTiabikdaYiab=z8aMnaaBaaaleaacqqGgbGrcqqGgbGrcqqGbbqqcqGHsgIRcqqGgbGrcqqGbbqqcqqGdbWqaeqaaaGcbaGaeyOeI0Iae8NXdy2aaSbaaSqaaiabboeadjabbkfasjabgkziUkabbcfaqjabboeadjabbkfasbqabaGccqGHsislcqWFgpGzdaWgaaWcbaGaeeyqaeKaeeivaqLaeeiuaaLaeyOKH4QaeeyqaeKaeeiraqKaeeiuaafabeaakiabgkHiTiab=z8aMnaaBaaaleaacqqGbbqqcqqGnbqtcqqGqbaucqGHsgIRcqqGbbqqcqqGebarcqqGqbauaeqaaaaaaaa@D38C@
ADP	VcdCc,ADPdt=φGLU→G6P+φG6P→GLY+φG6P→GA3P+7φGLC→TGL+2φFFA→FAC+φCR→PCR+φATP→ADP+2φAMP→ADP−2φBPG→PYR−φSCoA→SUC−5.64φO2→H2O−φPCR→CR−2φADP→AMP MathType@MTEF@5@5@+=feaafiart1ev1aaatCvAUfKttLearuWrP9MDH5MBPbIqV92AaeXatLxBI9gBaebbnrfifHhDYfgasaacH8akY=wiFfYdH8Gipec8Eeeu0xXdbba9frFj0=OqFfea0dXdd9vqai=hGuQ8kuc9pgc9s8qqaq=dirpe0xb9q8qiLsFr0=vr0=vr0dc8meaabaqaciaacaGaaeqabaqabeGadaaakeaafaqadeWabaaabaGaemOvay1aaSbaaSqaaiabbogaJbqabaGcdaWcaaqaaiabdsgaKjabdoeadnaaBaaaleaacqqGJbWycqqGSaalcqqGbbqqcqqGebarcqqGqbauaeqaaaGcbaGaemizaqMaemiDaqhaaiabg2da9GGaciab=z8aMnaaBaaaleaacqqGhbWrcqqGmbatcqqGvbqvcqGHsgIRcqqGhbWrcqqG2aGncqqGqbauaeqaaOGaey4kaSIae8NXdy2aaSbaaSqaaiabbEeahjabbAda2iabbcfaqjabgkziUkabbEeahjabbYeamjabbMfazbqabaGccqGHRaWkcqWFgpGzdaWgaaWcbaGaee4raCKaeeOnayJaeeiuaaLaeyOKH4Qaee4raCKaeeyqaeKaee4mamJaeeiuaafabeaakiabgUcaRiabiEda3iab=z8aMnaaBaaaleaacqqGhbWrcqqGmbatcqqGdbWqcqGHsgIRcqqGubavcqqGhbWrcqqGmbataeqaaOGaey4kaSIaeGOmaiJae8NXdy2aaSbaaSqaaiabbAeagjabbAeagjabbgeabjabgkziUkabbAeagjabbgeabjabboeadbqabaaakeaacqGHRaWkcqWFgpGzdaWgaaWcbaGaee4qamKaeeOuaiLaeyOKH4QaeeiuaaLaee4qamKaeeOuaifabeaakiabgUcaRiab=z8aMnaaBaaaleaacqqGbbqqcqqGubavcqqGqbaucqGHsgIRcqqGbbqqcqqGebarcqqGqbauaeqaaOGaey4kaSIaeeOmaiJae8NXdy2aaSbaaSqaaiabbgeabjabb2eanjabbcfaqjabgkziUkabbgeabjabbseaejabbcfaqbqabaGccqGHsislcqaIYaGmcqWFgpGzdaWgaaWcbaGaeeOqaiKaeeiuaaLaee4raCKaeyOKH4QaeeiuaaLaeeywaKLaeeOuaifabeaakiabgkHiTiab=z8aMnaaBaaaleaacqqGtbWucqqGdbWqcqqGVbWBcqqGbbqqcqGHsgIRcqqGtbWucqqGvbqvcqqGdbWqaeqaaaGcbaGaeyOeI0IaeGynauJaeiOla4IaeGOnayJaeGinaqJae8NXdy2aaSbaaSqaaiabb+eapjabbkdaYiabgkziUkabbIeaijabbkdaYiabb+eapbqabaGccqGHsislcqWFgpGzdaWgaaWcbaGaeeiuaaLaee4qamKaeeOuaiLaeyOKH4Qaee4qamKaeeOuaifabeaakiabgkHiTiabikdaYiab=z8aMnaaBaaaleaacqqGbbqqcqqGebarcqqGqbaucqGHsgIRcqqGbbqqcqqGnbqtcqqGqbauaeqaaaaaaaa@D528@
AMP	VcdCc,AMPdt=φADP→AMP−φAMP→ADP MathType@MTEF@5@5@+=feaafiart1ev1aaatCvAUfKttLearuWrP9MDH5MBPbIqV92AaeXatLxBI9gBaebbnrfifHhDYfgasaacH8akY=wiFfYdH8Gipec8Eeeu0xXdbba9frFj0=OqFfea0dXdd9vqai=hGuQ8kuc9pgc9s8qqaq=dirpe0xb9q8qiLsFr0=vr0=vr0dc8meaabaqaciaacaGaaeqabaqabeGadaaakeaacqWGwbGvdaWgaaWcbaGaee4yamgabeaakmaalaaabaGaemizaqMaem4qam0aaSbaaSqaaiabbogaJjabbYcaSiabbgeabjabb2eanjabbcfaqbqabaaakeaacqWGKbazcqWG0baDaaGaeyypa0dcciGae8NXdy2aaSbaaSqaaiabbgeabjabbseaejabbcfaqjabgkziUkabbgeabjabb2eanjabbcfaqbqabaGccqGHsislcqWFgpGzdaWgaaWcbaGaeeyqaeKaeeyta0KaeeiuaaLaeyOKH4QaeeyqaeKaeeiraqKaeeiuaafabeaaaaa@510C@

## Appendix B: Metabolic reaction flux expressions

**Table 7:** 

1. Glucose Utilization	GLU + ATP → G6P + ADP
φGLU→G6P=VGLU→G6P(CATPCADPKATPADP+CATPCADP)(CGLUKGLU1+CGLUKGLU+CG6PKG6P) MathType@MTEF@5@5@+=feaafiart1ev1aaatCvAUfKttLearuWrP9MDH5MBPbIqV92AaeXatLxBI9gBaebbnrfifHhDYfgasaacH8akY=wiFfYdH8Gipec8Eeeu0xXdbba9frFj0=OqFfea0dXdd9vqai=hGuQ8kuc9pgc9s8qqaq=dirpe0xb9q8qiLsFr0=vr0=vr0dc8meaabaqaciaacaGaaeqabaqabeGadaaakeaaiiGacqWFgpGzdaWgaaWcbaGaee4raCKaeeitaWKaeeyvauLaeyOKH4Qaee4raCKaeeOnayJaeeiuaafakeqaaiabg2da9iabdAfawnaaBaaaleaacqqGhbWrcqqGmbatcqqGvbqvcqGHsgIRcqqGhbWrcqqG2aGncqqGqbauaOqabaWaaeWaaeaadaWcaaqaamaalaaabaGaem4qam0aaSbaaSqaaiabbgeabjabbsfaujabbcfaqbGcbeaaaeaacqWGdbWqdaWgaaWcbaGaeeyqaeKaeeiraqKaeeiuaafakeqaaaaaaeaacqWGlbWsdaWgaaWcbaWaaSaaaeaacqqGbbqqcqqGubavcqqGqbauaeaacqqGbbqqcqqGebarcqqGqbauaaaakeqaaiabgUcaRmaalaaabaGaem4qam0aaSbaaSqaaiabbgeabjabbsfaujabbcfaqbGcbeaaaeaacqWGdbWqdaWgaaWcbaGaeeyqaeKaeeiraqKaeeiuaafakeqaaaaaaaaacaGLOaGaayzkaaWaaeWaaeaadaWcaaqaamaalaaabaGaem4qam0cdaWgaaqaaiabbEeahjabbYeamjabbwfavbqabaaakeaacqWGlbWsdaWgaaWcbaGaee4raCKaeeitaWKaeeyvaufakeqaaaaaaeaacqqGXaqmcqGHRaWkdaWcaaqaaiabdoeadnaaBaaaleaacqqGhbWrcqqGmbatcqqGvbqvaOqabaaabaGaem4saS0aaSbaaSqaaiabbEeahjabbYeamjabbwfavbGcbeaaaaGaey4kaSYaaSaaaeaacqWGdbWqlmaaBaaabaGaee4raCKaeeOnayJaeeiuaafabeaaaOqaaiabdUealnaaBaaaleaacqqGhbWrcqqG2aGncqqGqbauaOqabaaaaaaaaiaawIcacaGLPaaaaaa@7F39@	
2. Glycogen Synthesis	G6P + ATP → GLY + ADP + 2 Pi
φG6P→GLY=VG6P→GLY(CATPCADPKATPADP+CATPCADP)(CG6PKG6P1+CG6PKG6P+CGLYKGLY+CPiKPi+CGLYKGLY⋅CPiKPi) MathType@MTEF@5@5@+=feaafiart1ev1aaatCvAUfKttLearuWrP9MDH5MBPbIqV92AaeXatLxBI9gBaebbnrfifHhDYfgasaacH8akY=wiFfYdH8Gipec8Eeeu0xXdbba9frFj0=OqFfea0dXdd9vqai=hGuQ8kuc9pgc9s8qqaq=dirpe0xb9q8qiLsFr0=vr0=vr0dc8meaabaqaciaacaGaaeqabaqabeGadaaakeaaiiGacqWFgpGzlmaaBaaabaGaee4raCKaeeOnayJaeeiuaaLaeyOKH4Qaee4raCKaeeitaWKaeeywaKfabeaakiabg2da9iabdAfawnaaBaaaleaacqqGhbWrcqqG2aGncqqGqbaucqGHsgIRcqqGhbWrcqqGmbatcqqGzbqwaOqabaWaaeWaaeaadaWcaaqaamaalaaabaGaem4qam0aaSbaaSqaaiabbgeabjabbsfaujabbcfaqbGcbeaaaeaacqWGdbWqdaWgaaWcbaGaeeyqaeKaeeiraqKaeeiuaafakeqaaaaaaeaacqWGlbWsdaWgaaWcbaWaaSaaaeaacqqGbbqqcqqGubavcqqGqbauaeaacqqGbbqqcqqGebarcqqGqbauaaaakeqaaiabgUcaRmaalaaabaGaem4qam0aaSbaaSqaaiabbgeabjabbsfaujabbcfaqbGcbeaaaeaacqWGdbWqdaWgaaWcbaGaeeyqaeKaeeiraqKaeeiuaafakeqaaaaaaaaacaGLOaGaayzkaaWaaeWaaeaadaWcaaqaamaalaaabaGaem4qam0cdaWgaaqaaiabbEeahjabbAda2iabbcfaqbqabaaakeaacqWGlbWsdaWgaaWcbaGaee4raCKaeeOnayJaeeiuaafakeqaaaaaaeaacqqGXaqmcqGHRaWkdaWcaaqaaiabdoeadTWaaSbaaeaacqqGhbWrcqqG2aGncqqGqbauaeqaaaGcbaGaem4saS0aaSbaaSqaaiabbEeahjabbAda2iabbcfaqbGcbeaaaaGaey4kaSYaaSaaaeaacqWGdbWqlmaaBaaabaGaee4raCKaeeitaWKaeeywaKfabeaaaOqaaiabdUealnaaBaaaleaacqqGhbWrcqqGmbatcqqGzbqwaOqabaaaaiabgUcaRmaalaaabaGaem4qam0cdaWgaaqaaiabbcfaqjabbMgaPbqabaaakeaacqWGlbWsdaWgaaWcbaGaeeiuaaLaeeyAaKgakeqaaaaacqGHRaWkdaWcaaqaaiabdoeadTWaaSbaaeaacqqGhbWrcqqGmbatcqqGzbqwaeqaaaGcbaGaem4saS0aaSbaaSqaaiabbEeahjabbYeamjabbMfazbGcbeaaaaGaeyyXIC9aaSaaaeaacqWGdbWqlmaaBaaabaGaeeiuaaLaeeyAaKgabeaaaOqaaiabdUealnaaBaaaleaacqqGqbaucqqGPbqAaOqabaaaaaaaaiaawIcacaGLPaaaaaa@9BD3@	
This is lumping of 4 reactions G6P ↔ G1P, G1P + UTP → UDP-GLC + 2 Pi, UDP-GLC + GLY_n _→ UDP + GLY_n+1_, and UDP + ATP → UTP + ADP.	
3. Glycogen Utilization	GLY + Pi + G6P
φGLY→G6P=VGLY→G6P(CAMPCATPKAMPATP+CAMPCATP)(CGLYKGLY⋅CPiKPi1+CGLYKGLY+CPiKPi+CGLYKGLY⋅CPiKPi+CG6PKG6P) MathType@MTEF@5@5@+=feaafiart1ev1aaatCvAUfKttLearuWrP9MDH5MBPbIqV92AaeXatLxBI9gBaebbnrfifHhDYfgasaacH8akY=wiFfYdH8Gipec8Eeeu0xXdbba9frFj0=OqFfea0dXdd9vqai=hGuQ8kuc9pgc9s8qqaq=dirpe0xb9q8qiLsFr0=vr0=vr0dc8meaabaqaciaacaGaaeqabaqabeGadaaakeaaiiGacqWFgpGzlmaaBaaabaGaee4raCKaeeitaWKaeeywaKLaeyOKH4Qaee4raCKaeeOnayJaeeiuaafabeaakiabg2da9iabdAfawnaaBaaaleaacqqGhbWrcqqGmbatcqqGzbqwcqGHsgIRcqqGhbWrcqqG2aGncqqGqbauaOqabaWaaeWaaeaadaWcaaqaamaalaaabaGaem4qam0aaSbaaSqaaiabbgeabjabb2eanjabbcfaqbqabaaakeaacqWGdbWqdaWgaaWcbaGaeeyqaeKaeeivaqLaeeiuaafabeaaaaaakeaacqWGlbWsdaWgaaWcbaWaaSaaaeaacqqGbbqqcqqGnbqtcqqGqbauaeaacqqGbbqqcqqGubavcqqGqbauaaaabeaakiabgUcaRmaalaaabaGaem4qam0aaSbaaSqaaiabbgeabjabb2eanjabbcfaqbqabaaakeaacqWGdbWqdaWgaaWcbaGaeeyqaeKaeeivaqLaeeiuaafabeaaaaaaaaGccaGLOaGaayzkaaWaaeWaaeaadaWcaaqaamaalaaabaGaem4qam0cdaWgaaqaaiabbEeahjabbYeamjabbMfazbqabaaakeaacqWGlbWsdaWgaaWcbaGaee4raCKaeeitaWKaeeywaKfakeqaaaaacqGHflY1daWcaaqaaiabdoeadTWaaSbaaeaacqqGqbaucqqGPbqAaeqaaaGcbaGaem4saS0aaSbaaSqaaiabbcfaqjabbMgaPbGcbeaaaaaabaGaeeymaeJaey4kaSYaaSaaaeaacqWGdbWqlmaaBaaabaGaee4raCKaeeitaWKaeeywaKfabeaaaOqaaiabdUealnaaBaaaleaacqqGhbWrcqqGmbatcqqGzbqwaOqabaaaaiabgUcaRmaalaaabaGaem4qam0cdaWgaaqaaiabbcfaqjabbMgaPbqabaaakeaacqWGlbWsdaWgaaWcbaGaeeiuaaLaeeyAaKgakeqaaaaacqGHRaWkdaWcaaqaaiabdoeadTWaaSbaaeaacqqGhbWrcqqGmbatcqqGzbqwaeqaaaGcbaGaem4saS0aaSbaaSqaaiabbEeahjabbYeamjabbMfazbGcbeaaaaGaeyyXIC9aaSaaaeaacqWGdbWqlmaaBaaabaGaeeiuaaLaeeyAaKgabeaaaOqaaiabdUealnaaBaaaleaacqqGqbaucqqGPbqAaOqabaaaaiabgUcaRmaalaaabaGaem4qam0cdaWgaaqaaiabbEeahjabbAda2iabbcfaqbqabaaakeaacqWGlbWsdaWgaaWcbaGaee4raCKaeeOnayJaeeiuaafakeqaaaaaaaaacaGLOaGaayzkaaaaaa@A679@	
This is lumping of 2 reactions GLY + Pi → G1P and G1P ↔ G6P. The activity of the enzyme *glycogen phosphorylase *is regulated by AMP and ATP [51]; AMP acts as a positive effector (activator) and ATP acts a negative effector (inhibitor) by competing with AMP. So the reaction is controlled by C_AMP_/C_ATP _concentration ratio.	
4. Glucose 6-Phosphate Breakdown	G6P + ATP → 2 GA3P + ADP
φG6P→GA3P=VG6P→GA3P(CAMPCATPKAMPATP+CAMPCATP)(CG6PKG6P1+CG6PKG6P+CGA3PKGA3P) MathType@MTEF@5@5@+=feaafiart1ev1aaatCvAUfKttLearuWrP9MDH5MBPbIqV92AaeXatLxBI9gBaebbnrfifHhDYfgasaacH8akY=wiFfYdH8Gipec8Eeeu0xXdbba9frFj0=OqFfea0dXdd9vqai=hGuQ8kuc9pgc9s8qqaq=dirpe0xb9q8qiLsFr0=vr0=vr0dc8meaabaqaciaacaGaaeqabaqabeGadaaakeaaiiGacqWFgpGzlmaaBaaabaGaee4raCKaeeOnayJaeeiuaaLaeyOKH4Qaee4raCKaeeyqaeKaee4mamJaeeiuaafabeaakiabg2da9iabdAfawnaaBaaaleaacqqGhbWrcqqG2aGncqqGqbaucqGHsgIRcqqGhbWrcqqGbbqqcqqGZaWmcqqGqbauaOqabaWaaeWaaeaadaWcaaqaamaalaaabaGaem4qam0aaSbaaSqaaiabbgeabjabb2eanjabbcfaqbGcbeaaaeaacqWGdbWqdaWgaaWcbaGaeeyqaeKaeeivaqLaeeiuaafakeqaaaaaaeaacqWGlbWsdaWgaaWcbaWaaSaaaeaacqqGbbqqcqqGnbqtcqqGqbauaeaacqqGbbqqcqqGubavcqqGqbauaaaakeqaaiabgUcaRmaalaaabaGaem4qam0aaSbaaSqaaiabbgeabjabb2eanjabbcfaqbGcbeaaaeaacqWGdbWqdaWgaaWcbaGaeeyqaeKaeeivaqLaeeiuaafakeqaaaaaaaaacaGLOaGaayzkaaWaaeWaaeaadaWcaaqaamaalaaabaGaem4qam0cdaWgaaqaaiabbEeahjabbAda2iabbcfaqbqabaaakeaacqWGlbWsdaWgaaWcbaGaee4raCKaeeOnayJaeeiuaafakeqaaaaaaeaacqqGXaqmcqGHRaWkdaWcaaqaaiabdoeadTWaaSbaaeaacqqGhbWrcqqG2aGncqqGqbauaeqaaaGcbaGaem4saS0aaSbaaSqaaiabbEeahjabbAda2iabbcfaqbGcbeaaaaGaey4kaSYaaSaaaeaacqWGdbWqdaWgaaWcbaGaee4raCKaeeyqaeKaee4mamJaeeiuaafabeaaaOqaaiabdUealnaaBaaaleaacqqGhbWrcqqGbbqqcqqGZaWmcqqGqbauaeqaaaaaaaaakiaawIcacaGLPaaaaaa@8237@	
This is lumping of 4 reactions G6P ↔ F6P, F6P + ATP → F16BP + ADP, F16BP ↔ DHAP + GA3P, and DHAP ↔ GA3P. The activity of the enzyme *phosphofructo kinase *in this reaction is assumed to be regulated by the energy metabolite concentration ratio C_AMP_/C_ATP_.	
5. Glyceraldehyde 3-Phosphate Breakdown	GA3P + Pi + NAD^+ ^→ BPG + NADH
φGA3P→BPG=VGA3P→BPG(CNAD+CNADHKNAD+NADH+CNAD+CNADH)(CGA3PKGA3P⋅CPiKPi1+CGA3PKGA3P+CPiKPi+CGA3PKGA3P⋅CPiKPi+CBPGKBPG) MathType@MTEF@5@5@+=feaafiart1ev1aaatCvAUfKttLearuWrP9MDH5MBPbIqV92AaeXatLxBI9gBaebbnrfifHhDYfgasaacH8akY=wiFfYdH8Gipec8Eeeu0xXdbba9frFj0=OqFfea0dXdd9vqai=hGuQ8kuc9pgc9s8qqaq=dirpe0xb9q8qiLsFr0=vr0=vr0dc8meaabaqaciaacaGaaeqabaqabeGadaaakeaaiiGacqWFgpGzlmaaBaaabaGaee4raCKaeeyqaeKaee4mamJaeeiuaaLaeyOKH4QaeeOqaiKaeeiuaaLaee4raCeabeaakiabg2da9iabdAfawnaaBaaaleaacqqGhbWrcqqGbbqqcqqGZaWmcqqGqbaucqGHsgIRcqqGcbGqcqqGqbaucqqGhbWraOqabaWaaeWaaeaadaWcaaqaamaalaaabaGaem4qam0aaSbaaSqaaiabb6eaojabbgeabjabbseaenaaCaaabeqaaiabgUcaRaaaaOqabaaabaGaem4qam0aaSbaaSqaaiabb6eaojabbgeabjabbseaejabbIeaibGcbeaaaaaabaGaem4saS0aaSbaaSqaamaalaaabaGaeeOta4KaeeyqaeKaeeiraq0aaWbaaWqabeaacqqGRaWkaaaaleaacqqGobGtcqqGbbqqcqqGebarcqqGibasaaaakeqaaiabgUcaRmaalaaabaGaem4qam0aaSbaaSqaaiabb6eaojabbgeabjabbseaenaaCaaabeqaaiabgUcaRaaaaOqabaaabaGaem4qam0aaSbaaSqaaiabb6eaojabbgeabjabbseaejabbIeaibGcbeaaaaaaaaGaayjkaiaawMcaamaabmaabaWaaSaaaeaadaWcaaqaaiabdoeadnaaBaaaleaacqqGhbWrcqqGbbqqcqqGZaWmcqqGqbauaeqaaaGcbaGaem4saS0aaSbaaSqaaiabbEeahjabbgeabjabbodaZiabbcfaqbqabaaaaOGaeyyXIC9aaSaaaeaacqWGdbWqlmaaBaaabaGaeeiuaaLaeeyAaKgabeaaaOqaaiabdUealnaaBaaaleaacqqGqbaucqqGPbqAaOqabaaaaaqaaiabbgdaXiabgUcaRmaalaaabaGaem4qam0aaSbaaSqaaiabbEeahjabbgeabjabbodaZiabbcfaqbqabaaakeaacqWGlbWsdaWgaaWcbaGaee4raCKaeeyqaeKaee4mamJaeeiuaafabeaaaaGccqGHRaWkdaWcaaqaaiabdoeadTWaaSbaaeaacqqGqbaucqqGPbqAaeqaaaGcbaGaem4saS0aaSbaaSqaaiabbcfaqjabbMgaPbGcbeaaaaGaey4kaSYaaSaaaeaacqWGdbWqdaWgaaWcbaGaee4raCKaeeyqaeKaee4mamJaeeiuaafabeaaaOqaaiabdUealnaaBaaaleaacqqGhbWrcqqGbbqqcqqGZaWmcqqGqbauaeqaaaaakiabgwSixpaalaaabaGaem4qam0cdaWgaaqaaiabbcfaqjabbMgaPbqabaaakeaacqWGlbWsdaWgaaWcbaGaeeiuaaLaeeyAaKgakeqaaaaacqGHRaWkdaWcaaqaaiabdoeadTWaaSbaaeaacqqGcbGqcqqGqbaucqqGhbWraeqaaaGcbaGaem4saS0aaSbaaSqaaiabbkeacjabbcfaqjabbEeahbGcbeaaaaaaaaGaayjkaiaawMcaaaaa@B2B6@	
6. Pyruvate Production	BPG + 2 ADP → PYR + 2 ATP
φBPG→PYR=VBPG→PYR(CADPCATPKADPATP+CADPCATP)(CBPGKBPG1+CBPGKBPG+CPYRKPYR) MathType@MTEF@5@5@+=feaafiart1ev1aaatCvAUfKttLearuWrP9MDH5MBPbIqV92AaeXatLxBI9gBaebbnrfifHhDYfgasaacH8akY=wiFfYdH8Gipec8Eeeu0xXdbba9frFj0=OqFfea0dXdd9vqai=hGuQ8kuc9pgc9s8qqaq=dirpe0xb9q8qiLsFr0=vr0=vr0dc8meaabaqaciaacaGaaeqabaqabeGadaaakeaaiiGacqWFgpGzlmaaBaaabaGaeeOqaiKaeeiuaaLaee4raCKaeyOKH4QaeeiuaaLaeeywaKLaeeOuaifabeaakiabg2da9iabdAfawnaaBaaaleaacqqGcbGqcqqGqbaucqqGhbWrcqGHsgIRcqqGqbaucqqGzbqwcqqGsbGuaOqabaWaaeWaaeaadaWcaaqaamaalaaabaGaem4qam0aaSbaaSqaaiabbgeabjabbseaejabbcfaqbGcbeaaaeaacqWGdbWqdaWgaaWcbaGaeeyqaeKaeeivaqLaeeiuaafakeqaaaaaaeaacqWGlbWsdaWgaaWcbaWaaSaaaeaacqqGbbqqcqqGebarcqqGqbauaeaacqqGbbqqcqqGubavcqqGqbauaaaakeqaaiabgUcaRmaalaaabaGaem4qam0aaSbaaSqaaiabbgeabjabbseaejabbcfaqbGcbeaaaeaacqWGdbWqdaWgaaWcbaGaeeyqaeKaeeivaqLaeeiuaafakeqaaaaaaaaacaGLOaGaayzkaaWaaeWaaeaadaWcaaqaamaalaaabaGaem4qam0cdaWgaaqaaiabbkeacjabbcfaqjabbEeahbqabaaakeaacqWGlbWsdaWgaaWcbaGaeeOqaiKaeeiuaaLaee4raCeakeqaaaaaaeaacqqGXaqmcqGHRaWkdaWcaaqaaiabdoeadTWaaSbaaeaacqqGcbGqcqqGqbaucqqGhbWraeqaaaGcbaGaem4saS0aaSbaaSqaaiabbkeacjabbcfaqjabbEeahbGcbeaaaaGaey4kaSYaaSaaaeaacqWGdbWqlmaaBaaabaGaeeiuaaLaeeywaKLaeeOuaifabeaaaOqaaiabdUealnaaBaaaleaacqqGqbaucqqGzbqwcqqGsbGuaOqabaaaaaaaaiaawIcacaGLPaaaaaa@7FF5@	
This is lumping of 4 reactions 13BPG + ADP ↔ 3PG + ATP, 3PG ↔ 2PG, 2PG ↔ PEP, and PEP+ADP → PYR + ATP.	
7. Pyruvate Reduction	PYR + NADH → LAC + NAD^+^
φPYR→LAC=VPYR→LAC(CNADHCNAD+KNADHNAD++CNADHCNAD+)(CPYRKPYR1+CPYRKPYR+CLACKLAC) MathType@MTEF@5@5@+=feaafiart1ev1aaatCvAUfKttLearuWrP9MDH5MBPbIqV92AaeXatLxBI9gBaebbnrfifHhDYfgasaacH8akY=wiFfYdH8Gipec8Eeeu0xXdbba9frFj0=OqFfea0dXdd9vqai=hGuQ8kuc9pgc9s8qqaq=dirpe0xb9q8qiLsFr0=vr0=vr0dc8meaabaqaciaacaGaaeqabaqabeGadaaakeaaiiGacqWFgpGzlmaaBaaabaGaeeiuaaLaeeywaKLaeeOuaiLaeyOKH4QaeeitaWKaeeyqaeKaee4qameabeaakiabg2da9iabdAfawnaaBaaaleaacqqGqbaucqqGzbqwcqqGsbGucqGHsgIRcqqGmbatcqqGbbqqcqqGdbWqaOqabaWaaeWaaeaadaWcaaqaamaalaaabaGaem4qam0aaSbaaSqaaiabb6eaojabbgeabjabbseaejabbIeaibGcbeaaaeaacqWGdbWqdaWgaaWcbaGaeeOta4KaeeyqaeKaeeiraq0aaWbaaWqabeaacqqGRaWkaaaakeqaaaaaaeaacqWGlbWsdaWgaaWcbaWaaSaaaeaacqqGobGtcqqGbbqqcqqGebarcqqGibasaeaacqqGobGtcqqGbbqqcqqGebardaahaaadbeqaaiabbUcaRaaaaaaakeqaaiabgUcaRmaalaaabaGaem4qam0aaSbaaSqaaiabb6eaojabbgeabjabbseaejabbIeaibGcbeaaaeaacqWGdbWqdaWgaaWcbaGaeeOta4KaeeyqaeKaeeiraq0aaWbaaWqabeaacqqGRaWkaaaakeqaaaaaaaaacaGLOaGaayzkaaWaaeWaaeaadaWcaaqaamaalaaabaGaem4qam0cdaWgaaqaaiabbcfaqjabbMfazjabbkfasbqabaaakeaacqWGlbWsdaWgaaWcbaGaeeiuaaLaeeywaKLaeeOuaifakeqaaaaaaeaacqqGXaqmcqGHRaWkdaWcaaqaaiabdoeadTWaaSbaaeaacqqGqbaucqqGzbqwcqqGsbGuaeqaaaGcbaGaem4saS0aaSbaaSqaaiabbcfaqjabbMfazjabbkfasbGcbeaaaaGaey4kaSYaaSaaaeaacqWGdbWqlmaaBaaabaGaeeitaWKaeeyqaeKaee4qameabeaaaOqaaiabdUealnaaBaaaleaacqqGmbatcqqGbbqqcqqGdbWqaOqabaaaaaaaaiaawIcacaGLPaaaaaa@8623@	
8. Lactate Oxidation	LAC + NAD^+ ^+ PYR + NADH
φLAC→PYR=VLAC→PYR(CNAD+CNADHKNAD+NADH+CNAD+CNADH)(CLACKLAC1+CLACKLAC+CPYRKPYR) MathType@MTEF@5@5@+=feaafiart1ev1aaatCvAUfKttLearuWrP9MDH5MBPbIqV92AaeXatLxBI9gBaebbnrfifHhDYfgasaacH8akY=wiFfYdH8Gipec8Eeeu0xXdbba9frFj0=OqFfea0dXdd9vqai=hGuQ8kuc9pgc9s8qqaq=dirpe0xb9q8qiLsFr0=vr0=vr0dc8meaabaqaciaacaGaaeqabaqabeGadaaakeaaiiGacqWFgpGzlmaaBaaabaGaeeitaWKaeeyqaeKaee4qamKaeyOKH4QaeeiuaaLaeeywaKLaeeOuaifabeaakiabg2da9iabdAfawnaaBaaaleaacqqGmbatcqqGbbqqcqqGdbWqcqGHsgIRcqqGqbaucqqGzbqwcqqGsbGuaOqabaWaaeWaaeaadaWcaaqaamaalaaabaGaem4qam0aaSbaaSqaaiabb6eaojabbgeabjabbseaenaaCaaabeqaaiabgUcaRaaaaOqabaaabaGaem4qam0aaSbaaSqaaiabb6eaojabbgeabjabbseaejabbIeaibGcbeaaaaaabaGaem4saS0aaSbaaSqaamaalaaabaGaeeOta4KaeeyqaeKaeeiraq0aaWbaaWqabeaacqqGRaWkaaaaleaacqqGobGtcqqGbbqqcqqGebarcqqGibasaaaakeqaaiabgUcaRmaalaaabaGaem4qam0aaSbaaSqaaiabb6eaojabbgeabjabbseaenaaCaaabeqaaiabgUcaRaaaaOqabaaabaGaem4qam0aaSbaaSqaaiabb6eaojabbgeabjabbseaejabbIeaibGcbeaaaaaaaaGaayjkaiaawMcaamaabmaabaWaaSaaaeaadaWcaaqaaiabdoeadTWaaSbaaeaacqqGmbatcqqGbbqqcqqGdbWqaeqaaaGcbaGaem4saS0aaSbaaSqaaiabbYeamjabbgeabjabboeadbGcbeaaaaaabaGaeeymaeJaey4kaSYaaSaaaeaacqWGdbWqlmaaBaaabaGaeeitaWKaeeyqaeKaee4qameabeaaaOqaaiabdUealnaaBaaaleaacqqGmbatcqqGbbqqcqqGdbWqaOqabaaaaiabgUcaRmaalaaabaGaem4qam0cdaWgaaqaaiabbcfaqjabbMfazjabbkfasbqabaaakeaacqWGlbWsdaWgaaWcbaGaeeiuaaLaeeywaKLaeeOuaifakeqaaaaaaaaacaGLOaGaayzkaaaaaa@8574@	
9. Alanine Production	PYR → ALA
φPYR→ALA=VPYR→ALA(CPYRKPYR1+CPYRKPYR+CALAKALA) MathType@MTEF@5@5@+=feaafiart1ev1aaatCvAUfKttLearuWrP9MDH5MBPbIqV92AaeXatLxBI9gBaebbnrfifHhDYfgasaacH8akY=wiFfYdH8Gipec8Eeeu0xXdbba9frFj0=OqFfea0dXdd9vqai=hGuQ8kuc9pgc9s8qqaq=dirpe0xb9q8qiLsFr0=vr0=vr0dc8meaabaqaciaacaGaaeqabaqabeGadaaakeaaiiGacqWFgpGzlmaaBaaabaGaeeiuaaLaeeywaKLaeeOuaiLaeyOKH4QaeeyqaeKaeeitaWKaeeyqaeeabeaakiabg2da9iabdAfawnaaBaaaleaacqqGqbaucqqGzbqwcqqGsbGucqGHsgIRcqqGbbqqcqqGmbatcqqGbbqqaOqabaWaaeWaaeaadaWcaaqaamaalaaabaGaem4qam0cdaWgaaqaaiabbcfaqjabbMfazjabbkfasbqabaaakeaacqWGlbWsdaWgaaWcbaGaeeiuaaLaeeywaKLaeeOuaifakeqaaaaaaeaacqqGXaqmcqGHRaWkdaWcaaqaaiabdoeadTWaaSbaaeaacqqGqbaucqqGzbqwcqqGsbGuaeqaaaGcbaGaem4saS0aaSbaaSqaaiabbcfaqjabbMfazjabbkfasbGcbeaaaaGaey4kaSYaaSaaaeaacqWGdbWqlmaaBaaabaGaeeyqaeKaeeitaWKaeeyqaeeabeaaaOqaaiabdUealnaaBaaaleaacqqGbbqqcqqGmbatcqqGbbqqaOqabaaaaaaaaiaawIcacaGLPaaaaaa@6337@	
10. Pyruvate Oxidation	PYR + CoA + NAD^+ ^→ ACoA + NADH + CO_2_
φPYR→ACoA=VPYR→ACoA(CNAD+CNADHKNAD+NADH+CNAD+CNADH)(CPYRKPYR⋅CCoAKCoA1+CPYRKPYR+CCoAKCoA+CPYRKPYR⋅CCoAKCoA+CACoAKACoA+CCO2KCO2+CACoAKACoA⋅CCO2KCO2) MathType@MTEF@5@5@+=feaafiart1ev1aaatCvAUfKttLearuWrP9MDH5MBPbIqV92AaeXatLxBI9gBaebbnrfifHhDYfgasaacH8akY=wiFfYdH8Gipec8Eeeu0xXdbba9frFj0=OqFfea0dXdd9vqai=hGuQ8kuc9pgc9s8qqaq=dirpe0xb9q8qiLsFr0=vr0=vr0dc8meaabaqaciaacaGaaeqabaqabeGadaaakeaaiiGacqWFgpGzlmaaBaaabaGaeeiuaaLaeeywaKLaeeOuaiLaeyOKH4QaeeyqaeKaee4qamKaee4Ba8Maeeyqaeeabeaakiabg2da9iabdAfawnaaBaaaleaacqqGqbaucqqGzbqwcqqGsbGucqGHsgIRcqqGbbqqcqqGdbWqcqqGVbWBcqqGbbqqaOqabaWaaeWaaeaadaWcaaqaamaalaaabaGaem4qam0aaSbaaSqaaiabb6eaojabbgeabjabbseaenaaCaaabeqaaiabgUcaRaaaaOqabaaabaGaem4qam0aaSbaaSqaaiabb6eaojabbgeabjabbseaejabbIeaibGcbeaaaaaabaGaem4saS0aaSbaaSqaamaalaaabaGaeeOta4KaeeyqaeKaeeiraq0aaWbaaWqabeaacqqGRaWkaaaaleaacqqGobGtcqqGbbqqcqqGebarcqqGibasaaaakeqaaiabgUcaRmaalaaabaGaem4qam0aaSbaaSqaaiabb6eaojabbgeabjabbseaenaaCaaabeqaaiabgUcaRaaaaOqabaaabaGaem4qam0aaSbaaSqaaiabb6eaojabbgeabjabbseaejabbIeaibGcbeaaaaaaaaGaayjkaiaawMcaamaabmaabaWaaSaaaeaadaWcaaqaaiabdoeadnaaBaaaleaacqqGqbaucqqGzbqwcqqGsbGuaOqabaaabaGaem4saS0aaSbaaSqaaiabbcfaqjabbMfazjabbkfasbGcbeaaaaGaeyyXIC9aaSaaaeaacqWGdbWqdaWgaaWcbaGaee4qamKaee4Ba8MaeeyqaeeakeqaaaqaaiabdUealnaaBaaaleaacqqGdbWqcqqGVbWBcqqGbbqqaOqabaaaaaqaaiabbgdaXiabgUcaRmaalaaabaGaem4qam0aaSbaaSqaaiabbcfaqjabbMfazjabbkfasbGcbeaaaeaacqWGlbWsdaWgaaWcbaGaeeiuaaLaeeywaKLaeeOuaifakeqaaaaacqGHRaWkdaWcaaqaaiabdoeadnaaBaaaleaacqqGdbWqcqqGVbWBcqqGbbqqaOqabaaabaGaem4saS0aaSbaaSqaaiabboeadjabb+gaVjabbgeabbGcbeaaaaGaey4kaSYaaSaaaeaacqWGdbWqdaWgaaWcbaGaeeiuaaLaeeywaKLaeeOuaifakeqaaaqaaiabdUealnaaBaaaleaacqqGqbaucqqGzbqwcqqGsbGuaOqabaaaaiabgwSixpaalaaabaGaem4qam0aaSbaaSqaaiabboeadjabb+gaVjabbgeabbGcbeaaaeaacqWGlbWsdaWgaaWcbaGaee4qamKaee4Ba8MaeeyqaeeakeqaaaaacqGHRaWkdaWcaaqaaiabdoeadnaaBaaaleaacqqGbbqqcqqGdbWqcqqGVbWBcqqGbbqqaOqabaaabaGaem4saS0aaSbaaSqaaiabbgeabjabboeadjabb+gaVjabbgeabbGcbeaaaaGaey4kaSYaaSaaaeaacqWGdbWqdaWgaaWcbaGaee4qamKaee4ta8KaeeOmaidakeqaaaqaaiabdUealnaaBaaaleaacqqGdbWqcqqGpbWtcqqGYaGmaOqabaaaaiabgUcaRmaalaaabaGaem4qam0aaSbaaSqaaiabbgeabjabboeadjabb+gaVjabbgeabbGcbeaaaeaacqWGlbWsdaWgaaWcbaGaeeyqaeKaee4qamKaee4Ba8MaeeyqaeeakeqaaaaacqGHflY1daWcaaqaaiabdoeadnaaBaaaleaacqqGdbWqcqqGpbWtcqqGYaGmaOqabaaabaGaem4saS0aaSbaaSqaaiabboeadjabb+eapjabbkdaYaGcbeaaaaaaaaGaayjkaiaawMcaaaaa@D9DC@	
This reaction links between glycolysis and TCA cycle inside the mitochondrial matrix and contributes to ACoA formation from the carbohydrates.	
11. Lipolysis	TGL → GLC + 3 FFA
φTGL→GLC=VTGL→GLC(CTGLKTGL1+CTGLKTGL+CFFAKFFA+CGLCKGLC+CFFAKFFA⋅CGLCKGLC) MathType@MTEF@5@5@+=feaafiart1ev1aaatCvAUfKttLearuWrP9MDH5MBPbIqV92AaeXatLxBI9gBaebbnrfifHhDYfgasaacH8akY=wiFfYdH8Gipec8Eeeu0xXdbba9frFj0=OqFfea0dXdd9vqai=hGuQ8kuc9pgc9s8qqaq=dirpe0xb9q8qiLsFr0=vr0=vr0dc8meaabaqaciaacaGaaeqabaqabeGadaaakeaaiiGacqWFgpGzlmaaBaaabaGaeeivaqLaee4raCKaeeitaWKaeyOKH4Qaee4raCKaeeitaWKaee4qameabeaakiabg2da9iabdAfawnaaBaaaleaacqqGubavcqqGhbWrcqqGmbatcqGHsgIRcqqGhbWrcqqGmbatcqqGdbWqaOqabaWaaeWaaeaadaWcaaqaamaalaaabaGaem4qam0aaSbaaSqaaiabbsfaujabbEeahjabbYeambGcbeaaaeaacqWGlbWsdaWgaaWcbaGaeeivaqLaee4raCKaeeitaWeakeqaaaaaaeaacqqGXaqmcqGHRaWkdaWcaaqaaiabdoeadnaaBaaaleaacqqGubavcqqGhbWrcqqGmbataOqabaaabaGaem4saS0aaSbaaSqaaiabbsfaujabbEeahjabbYeambGcbeaaaaGaey4kaSYaaSaaaeaacqWGdbWqdaWgaaWcbaGaeeOrayKaeeOrayKaeeyqaeeakeqaaaqaaiabdUealnaaBaaaleaacqqGgbGrcqqGgbGrcqqGbbqqaOqabaaaaiabgUcaRmaalaaabaGaem4qam0aaSbaaSqaaiabbEeahjabbYeamjabboeadbGcbeaaaeaacqWGlbWsdaWgaaWcbaGaee4raCKaeeitaWKaee4qameakeqaaaaacqGHRaWkdaWcaaqaaiabdoeadnaaBaaaleaacqqGgbGrcqqGgbGrcqqGbbqqaOqabaaabaGaem4saS0aaSbaaSqaaiabbAeagjabbAeagjabbgeabbGcbeaaaaGaeyyXIC9aaSaaaeaacqWGdbWqdaWgaaWcbaGaee4raCKaeeitaWKaee4qameakeqaaaqaaiabdUealnaaBaaaleaacqqGhbWrcqqGmbatcqqGdbWqaOqabaaaaaaaaiaawIcacaGLPaaaaaa@81D1@	
12. Triglyceride Synthesis	GLC + 3 FFA + 7 ATP → TGL + 7 ADP + 7 Pi
φGLC→TGL=VGLC→TGL(CATPCADPKATPADP+CATPCADP)(CFFAKFFA⋅CGLCKGLC1+CFFAKFFA+CGLCKGLC+CFFAKFFA⋅CGLCKGLC+CTGLKTGL+CPiKPi+CTGLKTGL⋅CPiKPi) MathType@MTEF@5@5@+=feaafiart1ev1aaatCvAUfKttLearuWrP9MDH5MBPbIqV92AaeXatLxBI9gBaebbnrfifHhDYfgasaacH8akY=wiFfYdH8Gipec8Eeeu0xXdbba9frFj0=OqFfea0dXdd9vqai=hGuQ8kuc9pgc9s8qqaq=dirpe0xb9q8qiLsFr0=vr0=vr0dc8meaabaqaciaacaGaaeqabaqabeGadaaakeaaiiGacqWFgpGzlmaaBaaabaGaee4raCKaeeitaWKaee4qamKaeyOKH4QaeeivaqLaee4raCKaeeitaWeabeaakiabg2da9iabdAfawnaaBaaaleaacqqGhbWrcqqGmbatcqqGdbWqcqGHsgIRcqqGubavcqqGhbWrcqqGmbataOqabaWaaeWaaeaadaWcaaqaamaalaaabaGaem4qam0aaSbaaSqaaiabbgeabjabbsfaujabbcfaqbGcbeaaaeaacqWGdbWqdaWgaaWcbaGaeeyqaeKaeeiraqKaeeiuaafakeqaaaaaaeaacqWGlbWsdaWgaaWcbaWaaSaaaeaacqqGbbqqcqqGubavcqqGqbauaeaacqqGbbqqcqqGebarcqqGqbauaaaakeqaaiabgUcaRmaalaaabaGaem4qam0aaSbaaSqaaiabbgeabjabbsfaujabbcfaqbGcbeaaaeaacqWGdbWqdaWgaaWcbaGaeeyqaeKaeeiraqKaeeiuaafakeqaaaaaaaaacaGLOaGaayzkaaWaaeWaaeaadaWcaaqaamaalaaabaGaem4qam0aaSbaaSqaaiabbAeagjabbAeagjabbgeabbGcbeaaaeaacqWGlbWsdaWgaaWcbaGaeeOrayKaeeOrayKaeeyqaeeakeqaaaaacqGHflY1daWcaaqaaiabdoeadnaaBaaaleaacqqGhbWrcqqGmbatcqqGdbWqaOqabaaabaGaem4saS0aaSbaaSqaaiabbEeahjabbYeamjabboeadbGcbeaaaaaabaGaeeymaeJaey4kaSYaaSaaaeaacqWGdbWqdaWgaaWcbaGaeeOrayKaeeOrayKaeeyqaeeakeqaaaqaaiabdUealnaaBaaaleaacqqGgbGrcqqGgbGrcqqGbbqqaOqabaaaaiabgUcaRmaalaaabaGaem4qam0aaSbaaSqaaiabbEeahjabbYeamjabboeadbGcbeaaaeaacqWGlbWsdaWgaaWcbaGaee4raCKaeeitaWKaee4qameakeqaaaaacqGHRaWkdaWcaaqaaiabdoeadnaaBaaaleaacqqGgbGrcqqGgbGrcqqGbbqqaOqabaaabaGaem4saS0aaSbaaSqaaiabbAeagjabbAeagjabbgeabbGcbeaaaaGaeyyXIC9aaSaaaeaacqWGdbWqdaWgaaWcbaGaee4raCKaeeitaWKaee4qameakeqaaaqaaiabdUealnaaBaaaleaacqqGhbWrcqqGmbatcqqGdbWqaOqabaaaaiabgUcaRmaalaaabaGaem4qam0aaSbaaSqaaiabbsfaujabbEeahjabbYeambGcbeaaaeaacqWGlbWsdaWgaaWcbaGaeeivaqLaee4raCKaeeitaWeakeqaaaaacqGHRaWkdaWcaaqaaiabdoeadnaaBaaaleaacqqGqbaucqqGPbqAaOqabaaabaGaem4saS0aaSbaaSqaaiabbcfaqjabbMgaPbGcbeaaaaGaey4kaSYaaSaaaeaacqWGdbWqdaWgaaWcbaGaeeivaqLaee4raCKaeeitaWeakeqaaaqaaiabdUealnaaBaaaleaacqqGubavcqqGhbWrcqqGmbataOqabaaaaiabgwSixpaalaaabaGaem4qam0aaSbaaSqaaiabbcfaqjabbMgaPbGcbeaaaeaacqWGlbWsdaWgaaWcbaGaeeiuaaLaeeyAaKgakeqaaaaaaaaacaGLOaGaayzkaaaaaa@C69F@	
This is lumping of 3 reactions GLC + ATP → G3P + ADP, G3P + 3FAC → TGL + 3CoA + Pi, 3FFA + 3CoA + 6ATP → 3FAC + 6ATP + 6Pi. For simplicity, TGL synthesis from DHAP or GA3P (glycolysis) and FAC has been ignored.	
13. Free Fatty Acid Activation	FFA + CoA + 2 ATP → FAC + 2 ADP + 2 Pi
φFFA→FAC=VFFA→FAC(CATPCADPKATPADP+CATPCADP)(CFFAKFFA⋅CCoAKCoA1+CFFAKFFA+CCoAKCoA+CFFAKFFA⋅CCoAKCoA+CFACKFAC+CPiKPi+CFACKFAC⋅CPiKPi) MathType@MTEF@5@5@+=feaafiart1ev1aaatCvAUfKttLearuWrP9MDH5MBPbIqV92AaeXatLxBI9gBaebbnrfifHhDYfgasaacH8akY=wiFfYdH8Gipec8Eeeu0xXdbba9frFj0=OqFfea0dXdd9vqai=hGuQ8kuc9pgc9s8qqaq=dirpe0xb9q8qiLsFr0=vr0=vr0dc8meaabaqaciaacaGaaeqabaqabeGadaaakeaaiiGacqWFgpGzlmaaBaaabaGaeeOrayKaeeOrayKaeeyqaeKaeyOKH4QaeeOrayKaeeyqaeKaee4qameabeaakiabg2da9iabdAfawnaaBaaaleaacqqGgbGrcqqGgbGrcqqGbbqqcqGHsgIRcqqGgbGrcqqGbbqqcqqGdbWqaOqabaWaaeWaaeaadaWcaaqaamaalaaabaGaem4qam0aaSbaaSqaaiabbgeabjabbsfaujabbcfaqbGcbeaaaeaacqWGdbWqdaWgaaWcbaGaeeyqaeKaeeiraqKaeeiuaafakeqaaaaaaeaacqWGlbWsdaWgaaWcbaWaaSaaaeaacqqGbbqqcqqGubavcqqGqbauaeaacqqGbbqqcqqGebarcqqGqbauaaaakeqaaiabgUcaRmaalaaabaGaem4qam0aaSbaaSqaaiabbgeabjabbsfaujabbcfaqbGcbeaaaeaacqWGdbWqdaWgaaWcbaGaeeyqaeKaeeiraqKaeeiuaafakeqaaaaaaaaacaGLOaGaayzkaaWaaeWaaeaadaWcaaqaamaalaaabaGaem4qam0aaSbaaSqaaiabbAeagjabbAeagjabbgeabbGcbeaaaeaacqWGlbWsdaWgaaWcbaGaeeOrayKaeeOrayKaeeyqaeeakeqaaaaacqGHflY1daWcaaqaaiabdoeadnaaBaaaleaacqqGdbWqcqqGVbWBcqqGbbqqaOqabaaabaGaem4saS0aaSbaaSqaaiabboeadjabb+gaVjabbgeabbGcbeaaaaaabaGaeeymaeJaey4kaSYaaSaaaeaacqWGdbWqdaWgaaWcbaGaeeOrayKaeeOrayKaeeyqaeeakeqaaaqaaiabdUealnaaBaaaleaacqqGgbGrcqqGgbGrcqqGbbqqaOqabaaaaiabgUcaRmaalaaabaGaem4qam0aaSbaaSqaaiabboeadjabb+gaVjabbgeabbGcbeaaaeaacqWGlbWsdaWgaaWcbaGaee4qamKaee4Ba8MaeeyqaeeakeqaaaaacqGHRaWkdaWcaaqaaiabdoeadnaaBaaaleaacqqGgbGrcqqGgbGrcqqGbbqqaOqabaaabaGaem4saS0aaSbaaSqaaiabbAeagjabbAeagjabbgeabbGcbeaaaaGaeyyXIC9aaSaaaeaacqWGdbWqdaWgaaWcbaGaee4qamKaee4Ba8MaeeyqaeeakeqaaaqaaiabdUealnaaBaaaleaacqqGdbWqcqqGVbWBcqqGbbqqaOqabaaaaiabgUcaRmaalaaabaGaem4qam0aaSbaaSqaaiabbAeagjabbgeabjabboeadbGcbeaaaeaacqWGlbWsdaWgaaWcbaGaeeOrayKaeeyqaeKaee4qameakeqaaaaacqGHRaWkdaWcaaqaaiabdoeadnaaBaaaleaacqqGqbaucqqGPbqAaOqabaaabaGaem4saS0aaSbaaSqaaiabbcfaqjabbMgaPbGcbeaaaaGaey4kaSYaaSaaaeaacqWGdbWqdaWgaaWcbaGaeeOrayKaeeyqaeKaee4qameakeqaaaqaaiabdUealnaaBaaaleaacqqGgbGrcqqGbbqqcqqGdbWqaOqabaaaaiabgwSixpaalaaabaGaem4qam0aaSbaaSqaaiabbcfaqjabbMgaPbGcbeaaaeaacqWGlbWsdaWgaaWcbaGaeeiuaaLaeeyAaKgakeqaaaaaaaaacaGLOaGaayzkaaaaaa@C67B@	
This is lumping of 2 reactions FFA + CoA + ATP → FAC + AMP + 2Pi and AMP + ATP ↔ 2 ADP.	
14. Fatty Acyl-CoA Oxidation	FAC + 7 CoA + (35/3) NAD^+ ^→ 8 ACoA + (35/3) NADH
φFAC→ACoA=VFAC→ACoA(CNAD+CNADHKNAD+NADH+CNAD+CNADH)(CFACKFAC⋅CCoAKCoA1+CFACKFAC+CCoAKCoA+CFACKFAC⋅CCoAKCoA+CACoAKACoA) MathType@MTEF@5@5@+=feaafiart1ev1aaatCvAUfKttLearuWrP9MDH5MBPbIqV92AaeXatLxBI9gBaebbnrfifHhDYfgasaacH8akY=wiFfYdH8Gipec8Eeeu0xXdbba9frFj0=OqFfea0dXdd9vqai=hGuQ8kuc9pgc9s8qqaq=dirpe0xb9q8qiLsFr0=vr0=vr0dc8meaabaqaciaacaGaaeqabaqabeGadaaakeaaiiGacqWFgpGzlmaaBaaabaGaeeOrayKaeeyqaeKaee4qamKaeyOKH4QaeeyqaeKaee4qamKaee4Ba8Maeeyqaeeabeaakiabg2da9iabdAfawnaaBaaaleaacqqGgbGrcqqGbbqqcqqGdbWqcqGHsgIRcqqGbbqqcqqGdbWqcqqGVbWBcqqGbbqqaOqabaWaaeWaaeaadaWcaaqaamaalaaabaGaem4qam0aaSbaaSqaaiabb6eaojabbgeabjabbseaenaaCaaabeqaaiabgUcaRaaaaOqabaaabaGaem4qam0aaSbaaSqaaiabb6eaojabbgeabjabbseaejabbIeaibGcbeaaaaaabaGaem4saS0aaSbaaSqaamaalaaabaGaeeOta4KaeeyqaeKaeeiraq0aaWbaaWqabeaacqGHRaWkaaaaleaacqqGobGtcqqGbbqqcqqGebarcqqGibasaaaakeqaaiabgUcaRmaalaaabaGaem4qam0aaSbaaSqaaiabb6eaojabbgeabjabbseaenaaCaaabeqaaiabgUcaRaaaaOqabaaabaGaem4qam0aaSbaaSqaaiabb6eaojabbgeabjabbseaejabbIeaibGcbeaaaaaaaaGaayjkaiaawMcaamaabmaabaWaaSaaaeaadaWcaaqaaiabdoeadnaaBaaaleaacqqGgbGrcqqGbbqqcqqGdbWqaOqabaaabaGaem4saS0aaSbaaSqaaiabbAeagjabbgeabjabboeadbGcbeaaaaGaeyyXIC9aaSaaaeaacqWGdbWqdaWgaaWcbaGaee4qamKaee4Ba8MaeeyqaeeakeqaaaqaaiabdUealnaaBaaaleaacqqGdbWqcqqGVbWBcqqGbbqqaOqabaaaaaqaaiabbgdaXiabgUcaRmaalaaabaGaem4qam0aaSbaaSqaaiabbAeagjabbgeabjabboeadbGcbeaaaeaacqWGlbWsdaWgaaWcbaGaeeOrayKaeeyqaeKaee4qameakeqaaaaacqGHRaWkdaWcaaqaaiabdoeadnaaBaaaleaacqqGdbWqcqqGVbWBcqqGbbqqaOqabaaabaGaem4saS0aaSbaaSqaaiabboeadjabb+gaVjabbgeabbGcbeaaaaGaey4kaSYaaSaaaeaacqWGdbWqdaWgaaWcbaGaeeOrayKaeeyqaeKaee4qameakeqaaaqaaiabdUealnaaBaaaleaacqqGgbGrcqqGbbqqcqqGdbWqaOqabaaaaiabgwSixpaalaaabaGaem4qam0aaSbaaSqaaiabboeadjabb+gaVjabbgeabbGcbeaaaeaacqWGlbWsdaWgaaWcbaGaee4qamKaee4Ba8MaeeyqaeeakeqaaaaacqGHRaWkdaWcaaqaaiabdoeadnaaBaaaleaacqqGbbqqcqqGdbWqcqqGVbWBcqqGbbqqaOqabaaabaGaem4saS0aaSbaaSqaaiabbgeabjabboeadjabb+gaVjabbgeabbGcbeaaaaaaaaGaayjkaiaawMcaaaaa@B549@	
This reaction producing ACoA from the activated fatty acid inside the mitochondrial matrix is highly complex. It is the result of combining 7 cycles of reactions in which each cycle consists of 4 enzymatic reactions. For simplicity, FAD and FADH_2 _are considered equivalent to 2/3 NAD^+ ^and 2/3 NADH in terms of the amount of ATP production (though they consume equal amount of O_2_).	
15. Citrate Production	ACoA + OXA → CIT + CoA
φACoA→CIT=VACoA→CIT(CACoAKACoA⋅COXAKOXA1+CACoAKACoA+COXAKOXA+CACoAKACoA⋅COXAKOXA+CCITKCIT+CCoAKCoA+CCITKCIT⋅CCoAKCoA) MathType@MTEF@5@5@+=feaafiart1ev1aaatCvAUfKttLearuWrP9MDH5MBPbIqV92AaeXatLxBI9gBaebbnrfifHhDYfgasaacH8akY=wiFfYdH8Gipec8Eeeu0xXdbba9frFj0=OqFfea0dXdd9vqai=hGuQ8kuc9pgc9s8qqaq=dirpe0xb9q8qiLsFr0=vr0=vr0dc8meaabaqaciaacaGaaeqabaqabeGadaaakeaaiiGacqWFgpGzlmaaBaaabaGaeeyqaeKaee4qamKaee4Ba8MaeeyqaeKaeyOKH4Qaee4qamKaeeysaKKaeeivaqfabeaakiabg2da9iabdAfawnaaBaaaleaacqqGbbqqcqqGdbWqcqqGVbWBcqqGbbqqcqGHsgIRcqqGdbWqcqqGjbqscqqGubavaOqabaWaaeWaaeaadaWcaaqaamaalaaabaGaem4qam0aaSbaaSqaaiabbgeabjabboeadjabb+gaVjabbgeabbGcbeaaaeaacqWGlbWsdaWgaaWcbaGaeeyqaeKaee4qamKaee4Ba8MaeeyqaeeakeqaaaaacqGHflY1daWcaaqaaiabdoeadnaaBaaaleaacqqGpbWtcqqGybawcqqGbbqqaOqabaaabaGaem4saS0aaSbaaSqaaiabb+eapjabbIfayjabbgeabbGcbeaaaaaabaGaeeymaeJaey4kaSYaaSaaaeaacqWGdbWqdaWgaaWcbaGaeeyqaeKaee4qamKaee4Ba8MaeeyqaeeakeqaaaqaaiabdUealnaaBaaaleaacqqGbbqqcqqGdbWqcqqGVbWBcqqGbbqqaOqabaaaaiabgUcaRmaalaaabaGaem4qam0aaSbaaSqaaiabb+eapjabbIfayjabbgeabbGcbeaaaeaacqWGlbWsdaWgaaWcbaGaee4ta8KaeeiwaGLaeeyqaeeakeqaaaaacqGHRaWkdaWcaaqaaiabdoeadnaaBaaaleaacqqGbbqqcqqGdbWqcqqGVbWBcqqGbbqqaOqabaaabaGaem4saS0aaSbaaSqaaiabbgeabjabboeadjabb+gaVjabbgeabbGcbeaaaaGaeyyXIC9aaSaaaeaacqWGdbWqdaWgaaWcbaGaee4ta8KaeeiwaGLaeeyqaeeakeqaaaqaaiabdUealnaaBaaaleaacqqGpbWtcqqGybawcqqGbbqqaOqabaaaaiabgUcaRmaalaaabaGaem4qam0aaSbaaSqaaiabboeadjabbMeajjabbsfaubGcbeaaaeaacqWGlbWsdaWgaaWcbaGaee4qamKaeeysaKKaeeivaqfakeqaaaaacqGHRaWkdaWcaaqaaiabdoeadnaaBaaaleaacqqGdbWqcqqGVbWBcqqGbbqqaOqabaaabaGaem4saS0aaSbaaSqaaiabboeadjabb+gaVjabbgeabbGcbeaaaaGaey4kaSYaaSaaaeaacqWGdbWqdaWgaaWcbaGaee4qamKaeeysaKKaeeivaqfakeqaaaqaaiabdUealnaaBaaaleaacqqGdbWqcqqGjbqscqqGubavaOqabaaaaiabgwSixpaalaaabaGaem4qam0aaSbaaSqaaiabboeadjabb+gaVjabbgeabbGcbeaaaeaacqWGlbWsdaWgaaWcbaGaee4qamKaee4Ba8MaeeyqaeeakeqaaaaaaaaacaGLOaGaayzkaaaaaa@B8A5@	
16. α-Ketoglutarate Production	CIT + NAD^+ ^→ AKG + NADH + CO_2_
φCIT→AKG=VCIT→AKG(CNAD+CNADHKNAD+NADH+CNAD+CNADH)(CCITKCIT1+CCITKCIT+CAKGKAKG+CCO2KCO2+CAKGKAKG⋅CCO2KCO2) MathType@MTEF@5@5@+=feaafiart1ev1aaatCvAUfKttLearuWrP9MDH5MBPbIqV92AaeXatLxBI9gBaebbnrfifHhDYfgasaacH8akY=wiFfYdH8Gipec8Eeeu0xXdbba9frFj0=OqFfea0dXdd9vqai=hGuQ8kuc9pgc9s8qqaq=dirpe0xb9q8qiLsFr0=vr0=vr0dc8meaabaqaciaacaGaaeqabaqabeGadaaakeaaiiGacqWFgpGzlmaaBaaabaGaee4qamKaeeysaKKaeeivaqLaeyOKH4QaeeyqaeKaee4saSKaee4raCeabeaakiabg2da9iabdAfawnaaBaaaleaacqqGdbWqcqqGjbqscqqGubavcqGHsgIRcqqGbbqqcqqGlbWscqqGhbWraOqabaWaaeWaaeaadaWcaaqaamaalaaabaGaem4qam0aaSbaaSqaaiabb6eaojabbgeabjabbseaenaaCaaabeqaaiabgUcaRaaaaOqabaaabaGaem4qam0aaSbaaSqaaiabb6eaojabbgeabjabbseaejabbIeaibGcbeaaaaaabaGaem4saS0aaSbaaSqaamaalaaabaGaeeOta4KaeeyqaeKaeeiraq0aaWbaaWqabeaacqqGRaWkaaaaleaacqqGobGtcqqGbbqqcqqGebarcqqGibasaaaakeqaaiabgUcaRmaalaaabaGaem4qam0aaSbaaSqaaiabb6eaojabbgeabjabbseaenaaCaaabeqaaiabgUcaRaaaaOqabaaabaGaem4qam0aaSbaaSqaaiabb6eaojabbgeabjabbseaejabbIeaibGcbeaaaaaaaaGaayjkaiaawMcaamaabmaabaWaaSaaaeaadaWcaaqaaiabdoeadnaaBaaaleaacqqGdbWqcqqGjbqscqqGubavaOqabaaabaGaem4saS0aaSbaaSqaaiabboeadjabbMeajjabbsfaubGcbeaaaaaabaGaeeymaeJaey4kaSYaaSaaaeaacqWGdbWqdaWgaaWcbaGaee4qamKaeeysaKKaeeivaqfakeqaaaqaaiabdUealnaaBaaaleaacqqGdbWqcqqGjbqscqqGubavaOqabaaaaiabgUcaRmaalaaabaGaem4qam0aaSbaaSqaaiabbgeabjabbUealjabbEeahbGcbeaaaeaacqWGlbWsdaWgaaWcbaGaeeyqaeKaee4saSKaee4raCeakeqaaaaacqGHRaWkdaWcaaqaaiabdoeadnaaBaaaleaacqqGdbWqcqqGpbWtcqqGYaGmaOqabaaabaGaem4saS0aaSbaaSqaaiabboeadjabb+eapjabbkdaYaGcbeaaaaGaey4kaSYaaSaaaeaacqWGdbWqdaWgaaWcbaGaeeyqaeKaee4saSKaee4raCeakeqaaaqaaiabdUealnaaBaaaleaacqqGbbqqcqqGlbWscqqGhbWraOqabaaaaiabgwSixpaalaaabaGaem4qam0aaSbaaSqaaiabboeadjabb+eapjabbkdaYaGcbeaaaeaacqWGlbWsdaWgaaWcbaGaee4qamKaee4ta8KaeeOmaidakeqaaaaaaaaacaGLOaGaayzkaaaaaa@A3EA@	
This is lumping of 2 enzymatic reactions CIT ↔ ICIT and ICIT + NAD^+ ^→ AKG + CO_2 _+ NADH.	
17. Succinyl-CoA Production	AKG + CoA + NAD^+ ^→ SCoA + NADH + CO_2_
φAKG→SCoA=VAKG→SCoA(CNAD+CNADHKNAD+NADH+CNAD+CNADH)(CAKGKAKG⋅CCoAKCoA1+CAKGKAKG+CCoAKCoA+CAKGKAKG⋅CCoAKCoA+CSCoAKSCoA+CCO2KCO2+CSCoAKSCoA⋅CCO2KCO2) MathType@MTEF@5@5@+=feaafiart1ev1aaatCvAUfKttLearuWrP9MDH5MBPbIqV92AaeXatLxBI9gBaebbnrfifHhDYfgasaacH8akY=wiFfYdH8Gipec8Eeeu0xXdbba9frFj0=OqFfea0dXdd9vqai=hGuQ8kuc9pgc9s8qqaq=dirpe0xb9q8qiLsFr0=vr0=vr0dc8meaabaqaciaacaGaaeqabaqabeGadaaakeaaiiGacqWFgpGzlmaaBaaabaGaeeyqaeKaee4saSKaee4raCKaeyOKH4Qaee4uamLaee4qamKaee4Ba8Maeeyqaeeabeaakiabg2da9iabdAfawnaaBaaaleaacqqGbbqqcqqGlbWscqqGhbWrcqGHsgIRcqqGtbWucqqGdbWqcqqGVbWBcqqGbbqqaOqabaWaaeWaaeaadaWcaaqaamaalaaabaGaem4qam0aaSbaaSqaaiabb6eaojabbgeabjabbseaenaaCaaabeqaaiabgUcaRaaaaOqabaaabaGaem4qam0aaSbaaSqaaiabb6eaojabbgeabjabbseaejabbIeaibGcbeaaaaaabaGaem4saS0aaSbaaSqaamaalaaabaGaeeOta4KaeeyqaeKaeeiraq0aaWbaaWqabeaacqqGRaWkaaaaleaacqqGobGtcqqGbbqqcqqGebarcqqGibasaaaakeqaaiabgUcaRmaalaaabaGaem4qam0aaSbaaSqaaiabb6eaojabbgeabjabbseaenaaCaaabeqaaiabgUcaRaaaaOqabaaabaGaem4qam0aaSbaaSqaaiabb6eaojabbgeabjabbseaejabbIeaibGcbeaaaaaaaaGaayjkaiaawMcaamaabmaabaWaaSaaaeaadaWcaaqaaiabdoeadnaaBaaaleaacqqGbbqqcqqGlbWscqqGhbWraOqabaaabaGaem4saS0aaSbaaSqaaiabbgeabjabbUealjabbEeahbGcbeaaaaGaeyyXIC9aaSaaaeaacqWGdbWqdaWgaaWcbaGaee4qamKaee4Ba8MaeeyqaeeakeqaaaqaaiabdUealnaaBaaaleaacqqGdbWqcqqGVbWBcqqGbbqqaOqabaaaaaqaaiabbgdaXiabgUcaRmaalaaabaGaem4qam0aaSbaaSqaaiabbgeabjabbUealjabbEeahbGcbeaaaeaacqWGlbWsdaWgaaWcbaGaeeyqaeKaee4saSKaee4raCeakeqaaaaacqGHRaWkdaWcaaqaaiabdoeadnaaBaaaleaacqqGdbWqcqqGVbWBcqqGbbqqaOqabaaabaGaem4saS0aaSbaaSqaaiabboeadjabb+gaVjabbgeabbGcbeaaaaGaey4kaSYaaSaaaeaacqWGdbWqdaWgaaWcbaGaeeyqaeKaee4saSKaee4raCeakeqaaaqaaiabdUealnaaBaaaleaacqqGbbqqcqqGlbWscqqGhbWraOqabaaaaiabgwSixpaalaaabaGaem4qam0aaSbaaSqaaiabboeadjabb+gaVjabbgeabbGcbeaaaeaacqWGlbWsdaWgaaWcbaGaee4qamKaee4Ba8MaeeyqaeeakeqaaaaacqGHRaWkdaWcaaqaaiabdoeadnaaBaaaleaacqqGtbWucqqGdbWqcqqGVbWBcqqGbbqqaOqabaaabaGaem4saS0aaSbaaSqaaiabbofatjabboeadjabb+gaVjabbgeabbGcbeaaaaGaey4kaSYaaSaaaeaacqWGdbWqdaWgaaWcbaGaee4qamKaee4ta8KaeeOmaidakeqaaaqaaiabdUealnaaBaaaleaacqqGdbWqcqqGpbWtcqqGYaGmaOqabaaaaiabgUcaRmaalaaabaGaem4qam0aaSbaaSqaaiabbofatjabboeadjabb+gaVjabbgeabbGcbeaaaeaacqWGlbWsdaWgaaWcbaGaee4uamLaee4qamKaee4Ba8MaeeyqaeeakeqaaaaacqGHflY1daWcaaqaaiabdoeadnaaBaaaleaacqqGdbWqcqqGpbWtcqqGYaGmaOqabaaabaGaem4saS0aaSbaaSqaaiabboeadjabb+eapjabbkdaYaGcbeaaaaaaaaGaayjkaiaawMcaaaaa@D834@	
18. Succinate Production	SCoA + ADP + Pi → SUC + CoA + ATP
φSCoA→SUC=VSCoA→SUC(CADPCATPKADPATP+CADPCATP)(CSCoAKSCoA⋅CPiKPi1+CSCoAKSCoA+CPiKPi+CSCoAKSCoA⋅CPiKPi+CSUCKSUC+CCoAKCoA+CSUCKSUC⋅CCoAKCoA) MathType@MTEF@5@5@+=feaafiart1ev1aaatCvAUfKttLearuWrP9MDH5MBPbIqV92AaeXatLxBI9gBaebbnrfifHhDYfgasaacH8akY=wiFfYdH8Gipec8Eeeu0xXdbba9frFj0=OqFfea0dXdd9vqai=hGuQ8kuc9pgc9s8qqaq=dirpe0xb9q8qiLsFr0=vr0=vr0dc8meaabaqaciaacaGaaeqabaqabeGadaaakeaaiiGacqWFgpGzlmaaBaaabaGaee4uamLaee4qamKaee4Ba8MaeeyqaeKaeyOKH4Qaee4uamLaeeyvauLaee4qameabeaakiabg2da9iabdAfawnaaBaaaleaacqqGtbWucqqGdbWqcqqGVbWBcqqGbbqqcqGHsgIRcqqGtbWucqqGvbqvcqqGdbWqaOqabaWaaeWaaeaadaWcaaqaamaalaaabaGaem4qam0aaSbaaSqaaiabbgeabjabbseaejabbcfaqbGcbeaaaeaacqWGdbWqdaWgaaWcbaGaeeyqaeKaeeivaqLaeeiuaafakeqaaaaaaeaacqWGlbWsdaWgaaWcbaWaaSaaaeaacqqGbbqqcqqGebarcqqGqbauaeaacqqGbbqqcqqGubavcqqGqbauaaaakeqaaiabgUcaRmaalaaabaGaem4qam0aaSbaaSqaaiabbgeabjabbseaejabbcfaqbGcbeaaaeaacqWGdbWqdaWgaaWcbaGaeeyqaeKaeeivaqLaeeiuaafakeqaaaaaaaaacaGLOaGaayzkaaWaaeWaaeaadaWcaaqaamaalaaabaGaem4qam0aaSbaaSqaaiabbofatjabboeadjabb+gaVjabbgeabbGcbeaaaeaacqWGlbWsdaWgaaWcbaGaee4uamLaee4qamKaee4Ba8MaeeyqaeeakeqaaaaacqGHflY1daWcaaqaaiabdoeadnaaBaaaleaacqqGqbaucqqGPbqAaOqabaaabaGaem4saS0aaSbaaSqaaiabbcfaqjabbMgaPbGcbeaaaaaabaGaeeymaeJaey4kaSYaaSaaaeaacqWGdbWqdaWgaaWcbaGaee4uamLaee4qamKaee4Ba8MaeeyqaeeakeqaaaqaaiabdUealnaaBaaaleaacqqGtbWucqqGdbWqcqqGVbWBcqqGbbqqaOqabaaaaiabgUcaRmaalaaabaGaem4qam0aaSbaaSqaaiabbcfaqjabbMgaPbGcbeaaaeaacqWGlbWsdaWgaaWcbaGaeeiuaaLaeeyAaKgakeqaaaaacqGHRaWkdaWcaaqaaiabdoeadnaaBaaaleaacqqGtbWucqqGdbWqcqqGVbWBcqqGbbqqaOqabaaabaGaem4saS0aaSbaaSqaaiabbofatjabboeadjabb+gaVjabbgeabbGcbeaaaaGaeyyXIC9aaSaaaeaacqWGdbWqdaWgaaWcbaGaeeiuaaLaeeyAaKgakeqaaaqaaiabdUealnaaBaaaleaacqqGqbaucqqGPbqAaOqabaaaaiabgUcaRmaalaaabaGaem4qam0aaSbaaSqaaiabbofatjabbwfavjabboeadbGcbeaaaeaacqWGlbWsdaWgaaWcbaGaee4uamLaeeyvauLaee4qameakeqaaaaacqGHRaWkdaWcaaqaaiabdoeadnaaBaaaleaacqqGdbWqcqqGVbWBcqqGbbqqaOqabaaabaGaem4saS0aaSbaaSqaaiabboeadjabb+gaVjabbgeabbGcbeaaaaGaey4kaSYaaSaaaeaacqWGdbWqdaWgaaWcbaGaee4uamLaeeyvauLaee4qameakeqaaaqaaiabdUealnaaBaaaleaacqqGtbWucqqGvbqvcqqGdbWqaOqabaaaaiabgwSixpaalaaabaGaem4qam0aaSbaaSqaaiabboeadjabb+gaVjabbgeabbGcbeaaaeaacqWGlbWsdaWgaaWcbaGaee4qamKaee4Ba8MaeeyqaeeakeqaaaaaaaaacaGLOaGaayzkaaaaaa@D1D9@	
Because the reaction GTP + ADP ↔ GDP + ATP is in fast equilibrium, we assume the GTP/GDP ratio proportional to the ATP/ADP ratio.	
19. Malate Production	SUC + (2/3) NAD^+ ^→ MAL + (2/3) NADH
φSUC→MAL=VSUC→MAL(CNAD+CNADHKNAD+NADH+CNAD+CNADH)(CSUCKSUC1+CSUCKSUC+CMALKMAL) MathType@MTEF@5@5@+=feaafiart1ev1aaatCvAUfKttLearuWrP9MDH5MBPbIqV92AaeXatLxBI9gBaebbnrfifHhDYfgasaacH8akY=wiFfYdH8Gipec8Eeeu0xXdbba9frFj0=OqFfea0dXdd9vqai=hGuQ8kuc9pgc9s8qqaq=dirpe0xb9q8qiLsFr0=vr0=vr0dc8meaabaqaciaacaGaaeqabaqabeGadaaakeaaiiGacqWFgpGzlmaaBaaabaGaee4uamLaeeyvauLaee4qamKaeyOKH4Qaeeyta0KaeeyqaeKaeeitaWeabeaakiabg2da9iabdAfawnaaBaaaleaacqqGtbWucqqGvbqvcqqGdbWqcqGHsgIRcqqGnbqtcqqGbbqqcqqGmbataOqabaWaaeWaaeaadaWcaaqaamaalaaabaGaem4qam0aaSbaaSqaaiabb6eaojabbgeabjabbseaenaaCaaabeqaaiabgUcaRaaaaOqabaaabaGaem4qam0aaSbaaSqaaiabb6eaojabbgeabjabbseaejabbIeaibGcbeaaaaaabaGaem4saS0aaSbaaSqaamaalaaabaGaeeOta4KaeeyqaeKaeeiraq0aaWbaaWqabeaacqqGRaWkaaaaleaacqqGobGtcqqGbbqqcqqGebarcqqGibasaaaakeqaaiabgUcaRmaalaaabaGaem4qam0aaSbaaSqaaiabb6eaojabbgeabjabbseaenaaCaaabeqaaiabgUcaRaaaaOqabaaabaGaem4qam0aaSbaaSqaaiabb6eaojabbgeabjabbseaejabbIeaibGcbeaaaaaaaaGaayjkaiaawMcaamaabmaabaWaaSaaaeaadaWcaaqaaiabdoeadTWaaSbaaeaacqqGtbWucqqGvbqvcqqGdbWqaeqaaaGcbaGaem4saS0aaSbaaSqaaiabbofatjabbwfavjabboeadbGcbeaaaaaabaGaeeymaeJaey4kaSYaaSaaaeaacqWGdbWqlmaaBaaabaGaee4uamLaeeyvauLaee4qameabeaaaOqaaiabdUealnaaBaaaleaacqqGtbWucqqGvbqvcqqGdbWqaOqabaaaaiabgUcaRmaalaaabaGaem4qam0cdaWgaaqaaiabb2eanjabbgeabjabbYeambqabaaakeaacqWGlbWsdaWgaaWcbaGaeeyta0KaeeyqaeKaeeitaWeakeqaaaaaaaaacaGLOaGaayzkaaaaaa@85B0@	
This is lumping of 2 reactions SUC + FAD → FUM + FADH_2 _and FUM ↔ MAL. FAD and FADH_2 _are considered equivalent to 2/3 NAD^+ ^and 2/3 NADH.	
20. Oxaloacetate Production	MAL + NAD^+ ^→ OXA + NADH
φMAL→OXA=VMAL→OXA(CNAD+CNADHKNAD+NADH+CNAD+CNADH)(CMALKMAL1+CMALKMAL+COXAKOXA) MathType@MTEF@5@5@+=feaafiart1ev1aaatCvAUfKttLearuWrP9MDH5MBPbIqV92AaeXatLxBI9gBaebbnrfifHhDYfgasaacH8akY=wiFfYdH8Gipec8Eeeu0xXdbba9frFj0=OqFfea0dXdd9vqai=hGuQ8kuc9pgc9s8qqaq=dirpe0xb9q8qiLsFr0=vr0=vr0dc8meaabaqaciaacaGaaeqabaqabeGadaaakeaaiiGacqWFgpGzlmaaBaaabaGaeeyta0KaeeyqaeKaeeitaWKaeyOKH4Qaee4ta8KaeeiwaGLaeeyqaeeabeaakiabg2da9iabdAfawnaaBaaaleaacqqGnbqtcqqGbbqqcqqGmbatcqGHsgIRcqqGpbWtcqqGybawcqqGbbqqaOqabaWaaeWaaeaadaWcaaqaamaalaaabaGaem4qam0aaSbaaSqaaiabb6eaojabbgeabjabbseaenaaCaaabeqaaiabgUcaRaaaaOqabaaabaGaem4qam0aaSbaaSqaaiabb6eaojabbgeabjabbseaejabbIeaibGcbeaaaaaabaGaem4saS0aaSbaaSqaamaalaaabaGaeeOta4KaeeyqaeKaeeiraq0aaWbaaWqabeaacqqGRaWkaaaaleaacqqGobGtcqqGbbqqcqqGebarcqqGibasaaaakeqaaiabgUcaRmaalaaabaGaem4qam0aaSbaaSqaaiabb6eaojabbgeabjabbseaenaaCaaabeqaaiabgUcaRaaaaOqabaaabaGaem4qam0aaSbaaSqaaiabb6eaojabbgeabjabbseaejabbIeaibGcbeaaaaaaaaGaayjkaiaawMcaamaabmaabaWaaSaaaeaadaWcaaqaaiabdoeadTWaaSbaaeaacqqGnbqtcqqGbbqqcqqGmbataeqaaaGcbaGaem4saS0aaSbaaSqaaiabb2eanjabbgeabjabbYeambGcbeaaaaaabaGaeeymaeJaey4kaSYaaSaaaeaacqWGdbWqlmaaBaaabaGaeeyta0KaeeyqaeKaeeitaWeabeaaaOqaaiabdUealnaaBaaaleaacqqGnbqtcqqGbbqqcqqGmbataOqabaaaaiabgUcaRmaalaaabaGaem4qam0cdaWgaaqaaiabb+eapjabbIfayjabbgeabbqabaaakeaacqWGlbWsdaWgaaWcbaGaee4ta8KaeeiwaGLaeeyqaeeakeqaaaaaaaaacaGLOaGaayzkaaaaaa@8554@	
21. Oxygen Utilization	O_2 _+ 5.64 ADP + 5.64 Pi + 1.88 NADH → 2 H_2_O + 5.64 ATP + 1.88 NAD^+^
φO2→H2O=VO2→H2O(CADPCATPKADPATP+CADPCATP)(CO2KO2⋅CPiKPi⋅CNADHKNADH1+CO2KO2+CPiKPi+CNADHKNADH+CO2KO2⋅CPiKPi⋅CNADHKNADH+CNAD+KNAD+) MathType@MTEF@5@5@+=feaafiart1ev1aaatCvAUfKttLearuWrP9MDH5MBPbIqV92AaeXatLxBI9gBaebbnrfifHhDYfgasaacH8akY=wiFfYdH8Gipec8Eeeu0xXdbba9frFj0=OqFfea0dXdd9vqai=hGuQ8kuc9pgc9s8qqaq=dirpe0xb9q8qiLsFr0=vr0=vr0dc8meaabaqaciaacaGaaeqabaqabeGadaaakeaaiiGacqWFgpGzlmaaBaaabaGaee4ta8KaeeOmaiJaeyOKH4QaeeisaGKaeeOmaiJaee4ta8eabeaakiabg2da9iabdAfawnaaBaaaleaacqqGpbWtcqqGYaGmcqGHsgIRcqqGibascqqGYaGmcqqGpbWtaOqabaWaaeWaaeaadaWcaaqaamaalaaabaGaem4qam0aaSbaaSqaaiabbgeabjabbseaejabbcfaqbGcbeaaaeaacqWGdbWqdaWgaaWcbaGaeeyqaeKaeeivaqLaeeiuaafakeqaaaaaaeaacqWGlbWsdaWgaaWcbaWaaSaaaeaacqqGbbqqcqqGebarcqqGqbauaeaacqqGbbqqcqqGubavcqqGqbauaaaakeqaaiabgUcaRmaalaaabaGaem4qam0aaSbaaSqaaiabbgeabjabbseaejabbcfaqbGcbeaaaeaacqWGdbWqdaWgaaWcbaGaeeyqaeKaeeivaqLaeeiuaafakeqaaaaaaaaacaGLOaGaayzkaaWaaeWaaeaadaWcaaqaamaalaaabaGaem4qam0aaSbaaSqaaiabb+eapjabbkdaYaGcbeaaaeaacqWGlbWsdaWgaaWcbaGaee4ta8KaeeOmaidakeqaaaaacqGHflY1daWcaaqaaiabdoeadnaaBaaaleaacqqGqbaucqqGPbqAaOqabaaabaGaem4saS0aaSbaaSqaaiabbcfaqjabbMgaPbGcbeaaaaGaeyyXIC9aaSaaaeaacqWGdbWqdaWgaaWcbaGaeeOta4KaeeyqaeKaeeiraqKaeeisaGeakeqaaaqaaiabdUealnaaBaaaleaacqqGobGtcqqGbbqqcqqGebarcqqGibasaOqabaaaaaqaaiabbgdaXiabgUcaRmaalaaabaGaem4qam0aaSbaaSqaaiabb+eapjabbkdaYaGcbeaaaeaacqWGlbWsdaWgaaWcbaGaee4ta8KaeeOmaidakeqaaaaacqGHRaWkdaWcaaqaaiabdoeadnaaBaaaleaacqqGqbaucqqGPbqAaOqabaaabaGaem4saS0aaSbaaSqaaiabbcfaqjabbMgaPbGcbeaaaaGaey4kaSYaaSaaaeaacqWGdbWqdaWgaaWcbaGaeeOta4KaeeyqaeKaeeiraqKaeeisaGeakeqaaaqaaiabdUealnaaBaaaleaacqqGobGtcqqGbbqqcqqGebarcqqGibasaOqabaaaaiabgUcaRmaalaaabaGaem4qam0aaSbaaSqaaiabb+eapjabbkdaYaGcbeaaaeaacqWGlbWsdaWgaaWcbaGaee4ta8KaeeOmaidakeqaaaaacqGHflY1daWcaaqaaiabdoeadnaaBaaaleaacqqGqbaucqqGPbqAaOqabaaabaGaem4saS0aaSbaaSqaaiabbcfaqjabbMgaPbGcbeaaaaGaeyyXIC9aaSaaaeaacqWGdbWqdaWgaaWcbaGaeeOta4KaeeyqaeKaeeiraqKaeeisaGeakeqaaaqaaiabdUealnaaBaaaleaacqqGobGtcqqGbbqqcqqGebarcqqGibasaOqabaaaaiabgUcaRmaalaaabaGaem4qam0aaSbaaSqaaiabb6eaojabbgeabjabbseaenaaCaaameqabaGaee4kaScaaaGcbeaaaeaacqWGlbWsdaWgaaWcbaGaeeOta4KaeeyqaeKaeeiraq0aaWbaaWqabeaacqqGRaWkaaaakeqaaaaaaaaacaGLOaGaayzkaaaaaa@C4D9@	
This is a sum of several enzymatic reactions at complex I – V that constitute the electron transport chain and oxidative phosphorylation in the mitochondrial matrix. FAD and FADH_2 _are considered equivalent to 2/3 NAD^+ ^and 2/3 NADH. Unlike other reactions, here NADH and NAD^+ ^are treated as substrates rather than controllers. Furthermore, 1 NADH is assumed to produce 3 ATP (i.e., P/O ratio = 3 for substrate NADH). To account for the proton leak, the stoichiometries for NADH and NAD^+ ^are adjusted to 1.88 and that for ATP, ADP and Pi are accordingly adjusted to 3*1.88 = 5.64.	
22. Phosphocreatine Breakdown	PCR + ADP → CR + ATP
φPCR→CR=VPCR→CR(CADPCATPKADPATP+CADPCATP)(CPCRKPCR1+CPCRKPCR+CCRKCR) MathType@MTEF@5@5@+=feaafiart1ev1aaatCvAUfKttLearuWrP9MDH5MBPbIqV92AaeXatLxBI9gBaebbnrfifHhDYfgasaacH8akY=wiFfYdH8Gipec8Eeeu0xXdbba9frFj0=OqFfea0dXdd9vqai=hGuQ8kuc9pgc9s8qqaq=dirpe0xb9q8qiLsFr0=vr0=vr0dc8meaabaqaciaacaGaaeqabaqabeGadaaakeaaiiGacqWFgpGzlmaaBaaabaGaeeiuaaLaee4qamKaeeOuaiLaeyOKH4Qaee4qamKaeeOuaifabeaakiabg2da9iabdAfawnaaBaaaleaacqqGqbaucqqGdbWqcqqGsbGucqGHsgIRcqqGdbWqcqqGsbGuaOqabaWaaeWaaeaadaWcaaqaamaalaaabaGaem4qam0aaSbaaSqaaiabbgeabjabbseaejabbcfaqbGcbeaaaeaacqWGdbWqdaWgaaWcbaGaeeyqaeKaeeivaqLaeeiuaafakeqaaaaaaeaacqWGlbWsdaWgaaWcbaWaaSaaaeaacqqGbbqqcqqGebarcqqGqbauaeaacqqGbbqqcqqGubavcqqGqbauaaaakeqaaiabgUcaRmaalaaabaGaem4qam0aaSbaaSqaaiabbgeabjabbseaejabbcfaqbGcbeaaaeaacqWGdbWqdaWgaaWcbaGaeeyqaeKaeeivaqLaeeiuaafakeqaaaaaaaaacaGLOaGaayzkaaWaaeWaaeaadaWcaaqaamaalaaabaGaem4qam0cdaWgaaqaaiabbcfaqjabboeadjabbkfasbqabaaakeaacqWGlbWsdaWgaaWcbaGaeeiuaaLaee4qamKaeeOuaifakeqaaaaaaeaacqqGXaqmcqGHRaWkdaWcaaqaaiabdoeadTWaaSbaaeaacqqGqbaucqqGdbWqcqqGsbGuaeqaaaGcbaGaem4saS0aaSbaaSqaaiabbcfaqjabboeadjabbkfasbGcbeaaaaGaey4kaSYaaSaaaeaacqWGdbWqlmaaBaaabaGaee4qamKaeeOuaifabeaaaOqaaiabdUealnaaBaaaleaacqqGdbWqcqqGsbGuaOqabaaaaaaaaiaawIcacaGLPaaaaaa@7B39@	
23. Phosphocreatine Synthesis	CR + ATP → PCR + ADP
φCR→PCR=VCR→PCR(CATPCADPKATPADP+CATPCADP)(CCRKCR1+CCRKCR+CPCRKPCR) MathType@MTEF@5@5@+=feaafiart1ev1aaatCvAUfKttLearuWrP9MDH5MBPbIqV92AaeXatLxBI9gBaebbnrfifHhDYfgasaacH8akY=wiFfYdH8Gipec8Eeeu0xXdbba9frFj0=OqFfea0dXdd9vqai=hGuQ8kuc9pgc9s8qqaq=dirpe0xb9q8qiLsFr0=vr0=vr0dc8meaabaqaciaacaGaaeqabaqabeGadaaakeaaiiGacqWFgpGzlmaaBaaabaGaee4qamKaeeOuaiLaeyOKH4QaemiuaaLaee4qamKaeeOuaifabeaakiabg2da9iabdAfawnaaBaaaleaacqqGdbWqcqqGsbGucqGHsgIRcqWGqbaucqqGdbWqcqqGsbGuaOqabaWaaeWaaeaadaWcaaqaamaalaaabaGaem4qam0aaSbaaSqaaiabbgeabjabbsfaujabbcfaqbGcbeaaaeaacqWGdbWqdaWgaaWcbaGaeeyqaeKaeeiraqKaeeiuaafakeqaaaaaaeaacqWGlbWsdaWgaaWcbaWaaSaaaeaacqqGbbqqcqqGubavcqqGqbauaeaacqqGbbqqcqqGebarcqqGqbauaaaakeqaaiabgUcaRmaalaaabaGaem4qam0aaSbaaSqaaiabbgeabjabbsfaujabbcfaqbGcbeaaaeaacqWGdbWqdaWgaaWcbaGaeeyqaeKaeeiraqKaeeiuaafakeqaaaaaaaaacaGLOaGaayzkaaWaaeWaaeaadaWcaaqaamaalaaabaGaem4qam0cdaWgaaqaaiabboeadjabbkfasbqabaaakeaacqWGlbWsdaWgaaWcbaGaee4qamKaeeOuaifakeqaaaaaaeaacqqGXaqmcqGHRaWkdaWcaaqaaiabdoeadTWaaSbaaeaacqqGdbWqcqqGsbGuaeqaaaGcbaGaem4saS0aaSbaaSqaaiabboeadjabbkfasbGcbeaaaaGaey4kaSYaaSaaaeaacqWGdbWqlmaaBaaabaGaeeiuaaLaee4qamKaeeOuaifabeaaaOqaaiabdUealnaaBaaaleaacqqGqbaucqqGdbWqcqqGsbGuaOqabaaaaaaaaiaawIcacaGLPaaaaaa@78EF@	
24. ATP Hydrolysis	ATP → ADP + Pi
φATP→ADP=VATP→ADP(CATPKATP1+CATPKATP) MathType@MTEF@5@5@+=feaafiart1ev1aaatCvAUfKttLearuWrP9MDH5MBPbIqV92AaeXatLxBI9gBaebbnrfifHhDYfgasaacH8akY=wiFfYdH8Gipec8Eeeu0xXdbba9frFj0=OqFfea0dXdd9vqai=hGuQ8kuc9pgc9s8qqaq=dirpe0xb9q8qiLsFr0=vr0=vr0dc8meaabaqaciaacaGaaeqabaqabeGadaaakeaaiiGacqWFgpGzdaWgaaWcbaGaeeyqaeKaeeivaqLaeeiuaaLaeyOKH4QaeeyqaeKaeeiraqKaeeiuaafabeaakiabg2da9iabdAfawnaaBaaaleaacqqGbbqqcqqGubavcqqGqbaucqGHsgIRcqqGbbqqcqqGebarcqqGqbauaeqaaOWaaeWaaeaadaWcaaqaamaalaaabaGaem4qam0aaSbaaSqaaiabbgeabjabbsfaujabbcfaqbqabaaakeaacqWGlbWsdaWgaaWcbaGaeeyqaeKaeeivaqLaeeiuaafabeaaaaaakeaacqaIXaqmcqGHRaWkdaWcaaqaaiabdoeadnaaBaaaleaacqqGbbqqcqqGubavcqqGqbauaeqaaaGcbaGaem4saS0aaSbaaSqaaiabbgeabjabbsfaujabbcfaqbqabaaaaaaaaOGaayjkaiaawMcaaaaa@5864@	
25. AMP Utilization	AMP + ATP → 2 ADP
φAMP→ADP=VAMP→ADP(CAMPKAMP⋅CATPKATP1+CAMPKAMP+CATPKATP+CAMPKAMP⋅CATPKATP+CADPKADP) MathType@MTEF@5@5@+=feaafiart1ev1aaatCvAUfKttLearuWrP9MDH5MBPbIqV92AaeXatLxBI9gBaebbnrfifHhDYfgasaacH8akY=wiFfYdH8Gipec8Eeeu0xXdbba9frFj0=OqFfea0dXdd9vqai=hGuQ8kuc9pgc9s8qqaq=dirpe0xb9q8qiLsFr0=vr0=vr0dc8meaabaqaciaacaGaaeqabaqabeGadaaakeaaiiGacqWFgpGzlmaaBaaabaGaeeyqaeKaeeyta0KaeeiuaaLaeyOKH4QaeeyqaeKaeeiraqKaeeiuaafabeaakiabg2da9iabdAfawnaaBaaaleaacqqGbbqqcqqGnbqtcqqGqbaucqGHsgIRcqqGbbqqcqqGebarcqqGqbauaOqabaWaaeWaaeaadaWcaaqaamaalaaabaGaem4qam0aaSbaaSqaaiabbgeabjabb2eanjabbcfaqbqabaaakeaacqWGlbWsdaWgaaWcbaGaeeyqaeKaeeyta0KaeeiuaafabeaaaaGccqGHflY1daWcaaqaaiabdoeadnaaBaaaleaacqqGbbqqcqqGubavcqqGqbauaOqabaaabaGaem4saS0aaSbaaSqaaiabbgeabjabbsfaujabbcfaqbGcbeaaaaaabaGaeeymaeJaey4kaSYaaSaaaeaacqWGdbWqdaWgaaWcbaGaeeyqaeKaeeyta0KaeeiuaafabeaaaOqaaiabdUealnaaBaaaleaacqqGbbqqcqqGnbqtcqqGqbauaeqaaaaakiabgUcaRmaalaaabaGaem4qam0aaSbaaSqaaiabbgeabjabbsfaujabbcfaqbGcbeaaaeaacqWGlbWsdaWgaaWcbaGaeeyqaeKaeeivaqLaeeiuaafakeqaaaaacqGHRaWkdaWcaaqaaiabdoeadnaaBaaaleaacqqGbbqqcqqGnbqtcqqGqbauaeqaaaGcbaGaem4saS0aaSbaaSqaaiabbgeabjabb2eanjabbcfaqbqabaaaaOGaeyyXIC9aaSaaaeaacqWGdbWqdaWgaaWcbaGaeeyqaeKaeeivaqLaeeiuaafakeqaaaqaaiabdUealnaaBaaaleaacqqGbbqqcqqGubavcqqGqbauaOqabaaaaiabgUcaRmaalaaabaGaem4qam0aaSbaaSqaaiabbgeabjabbseaejabbcfaqbqabaaakeaacqWGlbWsdaWgaaWcbaGaeeyqaeKaeeiraqKaeeiuaafabeaaaaaaaaGccaGLOaGaayzkaaaaaa@8DEF@	
26. AMP Production	2 ADP → AMP + ATP
φADP→AMP=VADP→AMP(CADPKADP1+CADPKADP+CAMPKAMP+CATPKATP+CAMPKAMP⋅CATPKATP) MathType@MTEF@5@5@+=feaafiart1ev1aaatCvAUfKttLearuWrP9MDH5MBPbIqV92AaeXatLxBI9gBaebbnrfifHhDYfgasaacH8akY=wiFfYdH8Gipec8Eeeu0xXdbba9frFj0=OqFfea0dXdd9vqai=hGuQ8kuc9pgc9s8qqaq=dirpe0xb9q8qiLsFr0=vr0=vr0dc8meaabaqaciaacaGaaeqabaqabeGadaaakeaaiiGacqWFgpGzdaWgaaWcbaGaeeyqaeKaeeiraqKaeeiuaaLaeyOKH4QaeeyqaeKaeeyta0Kaeeiuaafabeaakiabg2da9iabdAfawnaaBaaaleaacqqGbbqqcqqGebarcqqGqbaucqGHsgIRcqqGbbqqcqqGnbqtcqqGqbauaeqaaOWaaeWaaeaadaWcaaqaamaalaaabaGaem4qam0aaSbaaSqaaiabbgeabjabbseaejabbcfaqbqabaaakeaacqWGlbWsdaWgaaWcbaGaeeyqaeKaeeiraqKaeeiuaafabeaaaaaakeaacqqGXaqmcqGHRaWkdaWcaaqaaiabdoeadnaaBaaaleaacqqGbbqqcqqGebarcqqGqbauaeqaaaGcbaGaem4saS0aaSbaaSqaaiabbgeabjabbseaejabbcfaqbqabaaaaOGaey4kaSYaaSaaaeaacqWGdbWqdaWgaaWcbaGaeeyqaeKaeeyta0KaeeiuaafabeaaaOqaaiabdUealnaaBaaaleaacqqGbbqqcqqGnbqtcqqGqbauaeqaaaaakiabgUcaRmaalaaabaGaem4qam0aaSbaaSqaaiabbgeabjabbsfaujabbcfaqbGcbeaaaeaacqWGlbWsdaWgaaWcbaGaeeyqaeKaeeivaqLaeeiuaafakeqaaaaacqGHRaWkdaWcaaqaaiabdoeadnaaBaaaleaacqqGbbqqcqqGnbqtcqqGqbauaeqaaaGcbaGaem4saS0aaSbaaSqaaiabbgeabjabb2eanjabbcfaqbqabaaaaOGaeyyXIC9aaSaaaeaacqWGdbWqdaWgaaWcbaGaeeyqaeKaeeivaqLaeeiuaafakeqaaaqaaiabdUealnaaBaaaleaacqqGbbqqcqqGubavcqqGqbauaOqabaaaaaaaaiaawIcacaGLPaaaaaa@8219@	
